# Physical inactivity linked to depressive symptoms in people with and without diabetes: A longitudinal network analysis of health risk behaviours and individual depressive symptoms from the Survey of Health, Ageing and Retirement in Europe

**DOI:** 10.1177/13591053251334444

**Published:** 2025-04-25

**Authors:** Amy M McInerney, Sonya S Deschênes

**Affiliations:** 1 University College Dublin, Ireland; 2 Universität Tübingen, Germany

**Keywords:** depressive symptoms, diabetes, health-risk behaviours, network analysis

## Abstract

This study examined cross-sectional (Ising) and longitudinal (cross-lagged panel) networks of depressive symptoms and health risk behaviours in people with diabetes, compared to those without. Participants (*N* = 22,510) from the Survey of Health, Ageing and Retirement in Europe with (*n* = 1781) and without (*n* = 20,729) diabetes with full data on smoking, high alcohol consumption, low fruit and vegetable consumption, physical inactivity and depressive symptoms (EURO-D) were included. Network models indicated that physical inactivity was by far the most closely connected behaviour to depressive symptoms for both groups. Networks were more densely connected for people without diabetes. Differences in whether sleep problems and physical inactivity activated or were activated by depressive symptoms were observed between people with and without diabetes. Identifying structural, symptom-level differences between health risk behaviours and depressive symptoms associated with the presence of diabetes holds potential for informing precision medicine.

## Introduction

Diabetes mellitus (hereafter referred to as diabetes) refers to a collection of metabolic conditions marked by elevated blood glucose levels (Cho et al., 2018). People living with type 1 diabetes (T1D) or type 2 diabetes (T2D), two distinct types of diabetes related to pancreatic β-cell dysfunction, are two to three times more likely to experience depression than those without diabetes (Anderson et al., 2001; Farooqi et al., 2022; Roy and Lloyd, 2012). Depression tends to be highly persistent and recurrent in people with diabetes, even after initial treatment success (Andreoulakis et al., 2012). Depression in people with diabetes is significantly and consistently associated with diabetes complications (Nouwen et al., 2019), as well as increased mortality, higher healthcare costs, decreased quality of life, and difficulties in maintaining treatment recommendations (Egede and Ellis, 2010).

Health behaviours are the cornerstones of managing diabetes, a largely self-managed condition (i.e. through behaviours such as maintenance or increase in physical activity levels and diabetes-specific behaviours such as self-monitoring of blood glucose), and can influence mental health outcomes. Behavioural recommendations for people with both T1D and T2D include ceasing smoking, eating healthily, drinking alcohol in moderation, and engaging in physical activity (ElSayed et al., 2022). Engaging in the risky side of these health behaviours (smoking, nutrition, alcohol and physical activity) is among the leading causes of global morbidity and mortality worldwide (Lim et al., 2012) and may be particularly relevant when considering depression in people with diabetes. Health risk behaviours are associated with an increased risk of future depression in the general population (Cabello et al., 2017; Collins et al., 2023). Furthermore, a meta-analysis published in 2023 provided evidence that health-promoting interventions focussed on a combination of physical activity and following a healthy diet reduced depressive symptom summary scores in people with T2D (Koning et al., 2023). However, less is known about how health risk behaviours impact depression in people with diabetes. Some cross-sectional evidence suggests that depression is associated with smoking (Collins et al., 2009; Nanayakkara et al., 2018), and heavy alcohol consumption (Collins et al., 2009) in people with T1D and T2D, and that depressive symptoms are correlated with sedentary behaviour in women with T2D, but not men (Indelicato et al., 2018). Therefore, despite the importance placed on reducing these health risk behaviours in the management of diabetes, the nuanced effects of these behaviours on depression require further investigation.

Furthermore, the relationship between health behaviours and mental health could have additional complexity for people with diabetes. Many people with diabetes report feeling ‘burned-out’, stressed about managing weight, and anxious about the responsibility of their self-care (Alberti, 2002). Feelings of guilt are associated with lapses in health behaviour upkeep (Sebire et al., 2018) and progression to insulin therapy can be viewed by some people with T2D as a personal failure (Polonsky et al., 2005). Therefore, engaging in health risk behaviours may be associated with increased negative feelings, which in turn could increase vulnerability to depression. Understanding the interplay between health risk behaviours and individual depressive symptoms, and how these connections may evolve over time, could help us to better understand the pathogenesis of depressive symptoms in people with diabetes.

A complex dynamic systems approach (Cramer et al., 2016; van der Maas, 2024) may provide new insights into the relationships between health behaviours and depression in people with diabetes. This theoretical and statistical approach to depression is based on the notion that psychological symptoms impact and are impacted by other symptoms, and it is these symptom-level connections that hold the key to understanding psychopathology (Cramer et al., 2016). Network analysis allows us to model these individual symptoms as an interactive network, examining which individual symptoms exert the most influence on the network and which are most strongly connected to other symptoms, as well as identifying symptoms that may act as bridges between clusters or groups (Epskamp and Fried, 2018; Jones et al., 2021), such as between depressive symptoms and health risk behaviours. Previous research has used longitudinal network analysis to examine interconnections between individual depressive symptoms (Savelieva et al., 2021) and between individual health behaviours (van Allen and Presseau, 2024) over time. In examining the relationship between health risk behaviours and depressive symptoms, Wang et al. (2024) used a cross-sectional design and found the obesity-related eating behaviours of ‘eat at all different times’ and ‘when buying food, I am not content unless I buy more than necessary’ to be the most strongly connected to depression and anxiety severity, respectively.

To the best of our knowledge, networks of health behaviours and depressive symptoms have not been examined in the context of diabetes. Previously, we used psychological network modelling techniques to examine the connections between diabetes distress, depression, and anxiety symptoms in a group of individuals with T2D (McInerney et al., 2022). This approach uncovered new insights, such as the centrality of regimen- and physician-related diabetes distress problems and how feelings of failure and worry might act as bridges between mental health conditions in people with diabetes. However, this analysis was cross-sectional, as are more recent network analyses examining depressive symptoms in people with diabetes (Daneshmand et al., 2024; Zhang et al., 2024), limiting our ability to theorise about the direction of these connections. Moreover, we solely focussed on psychological items, omitting other factors such as behaviours, that might be relevant to developmental symptom-pathways (McInerney et al., 2022). Longitudinal symptom-level connections remain under-researched in the field of network psychometric analysis, despite their potential to reveal the temporal dynamics at play (Guloksuz et al., 2017). Given the gaps in understanding the longitudinal association between health risk behaviours and depressive symptoms in people with diabetes, longitudinal network analysis could allow us to identify the health risk behaviours that might activate specific depressive symptoms for people with diabetes, and by comparing to those without diabetes, identify symptoms-level pathways unique to diabetes.

The present study aimed to examine the interconnections between individual depressive symptoms and health risk behaviours among people with diabetes. A network approach to examining both cross-sectional and longitudinal associations between health risk behaviours and individual depressive symptoms in people with diabetes, compared to those without, was applied using data from the Survey of Health, Ageing, and Retirement in Europe (SHARE; Börsch-Supan et al., 2008). The longitudinal analysis used cross-lagged panel models to explore temporal interactions between individual depressive symptoms and, separately, depressive symptoms and health behaviours, over 2-years. The timeframes used in this study were determined by the structure of the dataset. However, similar 2-year follow-up periods have been shown to be adequate for investigating changes in depression among older adults (Harris et al., 2005; Schaakxs et al., 2018), as well as in studies applying cross-lagged panel models to investigate depressive symptom network trajectories within a 2-year period (Schlechter et al., 2023). In the additional cross-sectional analysis, we examined clusters of individual depressive symptoms and behaviours, bridges between symptoms and behaviours, and compared these between groups. We hypothesised that health risk behaviours and depressive symptoms would cluster in their respective groups (i.e. depressive symptoms or health risk behaviours) with bridging links between these clusters, and that there would be differences in the connectivity between health risk behaviours and depressive symptoms for people with and without diabetes. Specifically, given the importance of health behaviour engagement to diabetes management, we expected that the network would be denser and more strongly connected for people with diabetes than those without.

## Method

### Population

Data came from the fourth (2011) and fifth (2013) wave of the Survey of Health, Ageing, and Retirement in Europe (SHARE; Börsch-Supan et al., 2008). SHARE is a multinational cohort study drawing from representative samples of adults aged over 50 years in 17 (wave 4) and 15 (wave 5) European countries (and Israel), including Austria, Belgium, the Czech Republic, Denmark, Estonia, France, Germany, Hungary, Israel, Italy, Luxembourg, the Netherlands, Poland, Portugal, Slovenia, Spain, Sweden, Switzerland. The questions included in the survey are aimed at being multidisciplinary, longitudinal, and cross-national, covering areas related to health, socioeconomics, and social networks, and are harmonised with other large population-based cohort studies (Abduladze et al., 2013). Data collection interviews were conducted in the local language for each country (or where more than one language is spoken, several languages were offered, e.g. Arabic, Hebrew, and Russian in Israel, and German, French, and Italian in Switzerland) and were collected using computer-assisted personal interviews (CAPI; Börsch-Supan et al., 2008). SHARE was ethically reviewed and approved by the Ethics Committee of the University of Mannheim (waves 1–4) and the Ethics Council of the Max Planck Society (from wave 4 onwards).

**Figure 1.**
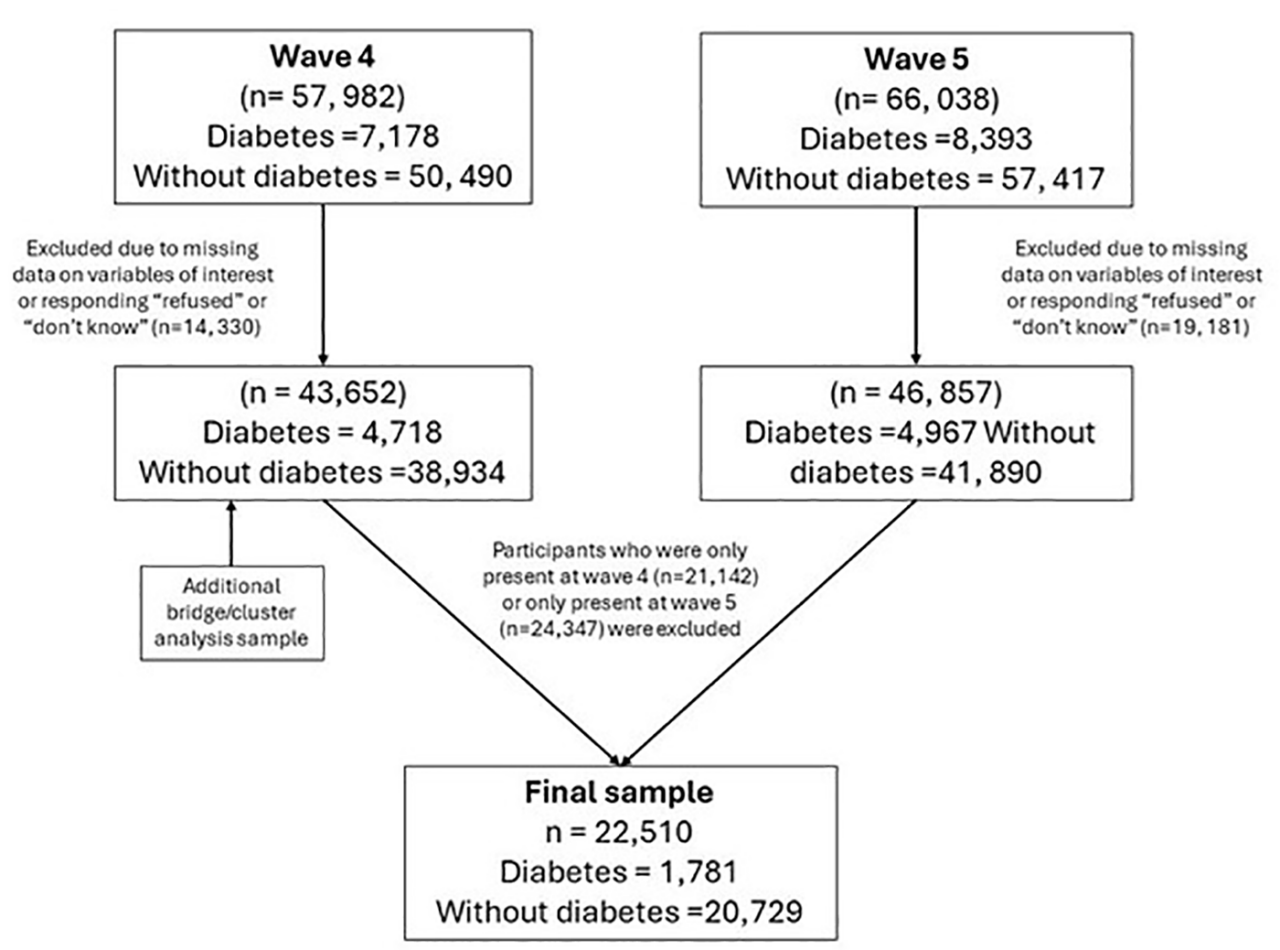
Participant selection flowchart.

The wave 4 original sample consisted of *n* = 57,982 and the wave 5 original sample consisted of *n* = 66,038 participants. Those with missing data on the diabetes question, or on the depression symptoms or health risk behaviour variables, were excluded. After excluding those with missing data and those who were only present at one wave (including *n* = 3186 people with diabetes who were either not present at wave 4 or were present at wave 4 but had new-onset diabetes at wave 5), the final analytical sample was *n* = 22,510 (*n* = 1781 with diabetes and *n* = 20,729 without diabetes). The wave 4 data from this sample was used for the cross-sectional analysis (described below). Figure 1 presents the participant selection flowchart.

### Measures

#### Diabetes

Diabetes was determined by a positive response to the question ‘Has a doctor ever told you that you had/Do you currently have: Diabetes or high blood sugar?’. Self-reported diagnosis of diabetes has high validity and is a reliable proxy for medical records in research (Comino et al., 2013; Jackson et al., 2014). This question did not differentiate between diabetes types (i.e., T1D, T2D or gestational). However, the International Diabetes Federation reports that approximately 95% of diabetes cases in Europe are people with T2D, and therefore our sample is likely to include people with all types of diabetes but be predominately people with T2D (International Diabetes Federation, 2023).

#### Depression

Depressive symptoms were assessed using the EURO-D 12 item scale (Prince et al., 1999). The EURO-D items have binary responses which indicate the presence (1) or absence (0) of a symptom. The EURO-D assesses depressive feelings of depression, pessimism, suicidality, guilt, sleep, (lack of) interest, irritability, (loss of) appetite, fatigue, (lack of) concentration, (lack of) enjoyment, and tearfulness. In the study sample, internal consistency of the EURO-D measure was questionable (α = 0.61 for Wave 4 and α = 0.65 for Wave 5). However, as this study does not use the EURO-D summary scale as a latent construct but focuses on the individual items themselves, of more relevance to the current study is whether any items measure the same construct (see goldbricker test in Data Analysis section).

#### Health risk behaviours

Current smoking status was determined by a positive response to the question ‘Do you smoke at the present time?’ (with ‘yes’ responses coded as 1). A negative response to this question or to the question ‘Have you ever smoked. .?’ was coded as not being a current smoker (coded as 0). For nutrition, we used a participant’s response to ‘In a regular week, how often do you consume a serving of fruits or vegetables?’ Participants were coded as daily eaters of fruit/vegetables (0) or less than daily eaters of fruit and vegetables (1). Heavy episodic alcohol drinking was determined by the question ‘In the last three months, how often did you have six or more drinks on one occasion?’, with those who did so at least once in the last 3 months coded as 1 and those who did not or who indicated they never drank alcohol coded as 0. Physical inactivity was determined by a generated variable based on responses to physical activity questions about moderate and physical activity and was coded as ‘never vigorous nor moderate physical activity’ (1) and other (0).

#### Additional variables for sample description

Additional variables used to describe the sample included wave 4 age, sex, marital status, years in education, number of limitations of activities of daily living, BMI, and self-perceived health.

### Data analysis

We used several network modelling techniques to estimate networks of behaviour and depressive symptoms cross-sectionally and longitudinally. Analysis was performed in R Version 4.3.1.

First, we used the goldbricker function in the R package ‘networktools’ (Jones and Jones, 2018) to test for overlapping pairs of items amongst the health risk behaviours and depressive symptoms in each network. Items were deemed redundant (i.e. likely to measure the same underlying construct) if less than 25% of their correlations (with the other items in the network) were significantly different from another item’s correlations (with the other items in the network).

Networks were visualised using the R package ‘qgraph’ (Epskamp et al., 2012), and consist of ‘nodes’, which represent the respective variables or items of interest (i.e. health behaviours and depressive symptoms), and ‘edges’, the conditional dependencies between nodes (Epskamp and Fried, 2018). An edge’s thickness represents the strength of association between two nodes, with blue edges denoting positive associations and red edges denoting negative associations. In visualising the networks, the ‘spring’ layout argument in the r package ‘qgraph’ (Epskamp et al., 2012) was applied, which positions nodes using the Fruchterman-Reingold algorithm (Fruchterman and Reingold, 1991). This algorithm uses force-directed placement which pulls connected nodes close to each other.

For the longitudinal model, we used cross-lagged panel network models (CLPN) to analyse how depressive symptoms and health risk behaviours (and separately, depressive symptoms alone, presented in Supplemental Material, section a) related to one another over time (Wysocki et al., 2022). We used the ‘glmnet’ R package (Friedman et al., 2010) to regress each node at wave 5 on all other nodes at wave 4 (cross-lagged effects) and itself (autoregressive effects). This method uses least absolute shrinkage and selection operator (LASSO) regularisation to shrink small regression paths to 0 (Wysocki et al., 2022).

Ising models were fit for the cross-sectional models reported in Supplemental Material (van Borkulo et al., 2014). This method estimates two sets of parameters: node threshold parameters, which represents the ‘preference’ of a node to be 0 or 1; and pairwise association parameters, which represent the logistic regression between nodes, with nodes regressed on all others in the network (van Borkulo et al., 2014). To limit spurious edges, the eLasso procedure was implemented in the IsingFit R package (van Borkulo et al., 2016). This procedure imposes a penalty on regression coefficients, favouring solutions that are more parsimonious, and is based on the extended Bayesian Information Criterion (EBIC; Chen and Chen, 2008). To examine clusters in the combined health risk behaviours and depressive networks we used the walktrap algorithm (Pons and Latapy, 2005) implemented in the EGAnet R package (Golino et al., 2020) which captures the community structure in a network through random walks. The EGA.plot function was used to plot the clusters which leverage ggplot2 (Wickham et al., 2016). Bridge symptoms or behaviours were examined to identify depressive symptoms that had very strong connections with health risk behaviours and health risk behaviours that had very strong connections with depressive symptoms. We investigated bridge strength, the total absolute sum of the edge weights linking a node in one group (e.g. depression) to nodes in the other (e.g. health risk behaviours), and bridge expected influence, the total non-absolute sum of all edges connecting a node to nodes of another group (Jones et al., 2021).

We computed the strength, reflecting the sum of absolute edge weights connecting a node to others (Epskamp et al., 2018), and expected influence, reflecting the sum of edges connecting a node to other nodes where positive edges can outweigh negative edges (Robinaugh et al., 2016). In the directed longitudinal CLPNs, we examined in- and out-expected influence and in- and out-strength, where we can differentiate a nodes’ role as recipient (in) or source (out) of activation of other nodes (Robinaugh et al., 2016). To assess the stability and accuracy of network indices, a post-hoc bootstrapping framework was used (Epskamp et al., 2018). The analysis was not pre-registered.

## Results

Sample characteristics are presented and compared in Table 1, along with depressive symptom scores and health behaviours at wave 4 and wave 5. People with and without diabetes differed significantly on most characteristics investigated. Those included in the sample differed significantly on most characteristics investigated to those excluded (see tables in Supplemental Material).

**Table 1.** Sample characteristics.

	With diabetes	Without diabetes	Difference test	Whole sample
Characteristic	*N* (%)/*M* (SD)	*N* (%)/*M* (SD)		*N* (%)/*M* (SD)
*N*	1781 (7.9%)	20,729 (92.1%)		22,510
Age in 2011	67.1 (8.8)	64.2 (9.4)	<0.001	64.4 (9.3)
Male or female				
Male	1094 (61.4%)	10,018 (48.3%)	<0.001	11,112 (49.4%)
Female	687 (38.6%)	10,711 (51.7%)		11,398 (50.6%)
Marital status				
Married and living together with spouse	714 (66.8%)	8709 (68.3%)	0.027	9423 (68.2%)
Registered partnership	17 (1.6%)	219 (1.7%)		236 (1.7%)
Married, living separated from spouse	21 (2.0%)	177 (1.4%)		198 (1.4%)
Never married	64 (6.0%)	807 (6.3%)		871 (6.3%)
Divorced	103 (9.6%)	1430 (11.2%)		1533 (11.1%)
Widowed	150 (14.0%)	1415 (11.1%)		1565 (11.3%)
Years of education	10.7 (4.3)	11.3 (4.4)	<0.001	11.2 (4.4)
Number of limitations with activities of daily living				
0	1571 (88.2%)	19,424 (93.7%)	<0.001	20,995 (93.3%)
1	131 (7.4%)	904 (4.4%)		1035 (4.6%)
2	47 (2.6%)	207 (1.0%)		254 (1.1%)
3	21 (1.2%)	111 (0.5%)		132 (0.6%)
4	4 (0.2%)	29 (0.1%)		33 (0.1%)
5	5 (0.3%)	24 (0.1%)		29 (0.1%)
6	2 (0.1%)	26 (0.1%)		28 (0.1%)
BMI	29.6 (4.9)	26.2 (4.2)	<0.001	26.5 (4.4)
Self-perceived health – US version				
Excellent	46 (2.6%)	2215 (10.7%)	<0.001	2261 (10.0%)
Very good	178 (10.0%)	4826 (23.3%)		5004 (22.2%)
Good	638 (35.8%)	8230 (39.7%)		8868 (39.4%)
Fair	675 (37.9%)	4523 (21.8%)		5198 (23.1%)
Poor	244 (13.7%)	930 (4.5%)		1174 (5.2%)
EURO-D caseness (wave 4)				
No	1315 (73.8%)	16,373 (79.0%)	<0.001	17,688 (78.6%)
Yes	466 (26.2%)	4356 (21.0%)		4822 (21.4%)
Smoking status (wave 4)				
Not present smoker	1476 (82.9%)	16,512 (79.7%)	0.001	17,988 (79.9%)
Present smoker	305 (17.1%)	4217 (20.3%)		4522 (20.1%)
Physical inactivity (wave 4)				
Other	1622 (91.1%)	19,725 (95.2%)	<0.001	21,347 (94.8%)
Never vigorous nor moderate physical activity	159 (8.9%)	1004 (4.8%)		1163 (5.2%)
How often serving of fruits or vegetables (wave 4)				
Eats daily	1400 (78.6%)	16,316 (78.7%)	0.918	17,716 (78.7%)
Eats less than every day	381 (21.4%)	4413 (21.3%)		4794 (21.3%)
Drink 6 or more alcoholic drinks in one sitting in last 3 months (wave 4)				
Never drinks more than 6 drinks	1346 (75.6%)	15,345 (74.0%)	0.152	16,691 (74.1%)
Drank 6 or more drinks at least once in last 3 months	435 (24.4%)	5384 (26.0%)		5819 (25.9%)
EURO-D caseness (wave 5)				
No	1304 (73.2%)	16,488 (79.5%)	<0.001	17,792 (79.0%)
Yes	477 (26.8%)	4241 (20.5%)		4718 (21.0%)
Smoking status (wave 5)				
No	1450 (81.4%)	16,552 (79.8%)	0.113	18,002 (80.0%)
Yes	331 (18.6%)	4177 (20.2%)		4508 (20.0%)
Physical inactivity (wave 5)				
Other	1585 (89.0%)	19,601 (94.6%)	<0.001	21,186 (94.1%)
Never vigorous nor moderate physical activity	196 (11.0%)	1128 (5.4%)		1324 (5.9%)
How often serving of fruits or vegetables (wave 5)				
Eats daily	1432 (80.4%)	16,890 (81.5%)	0.263	18,322 (81.4%)
Eats less than every day	349 (19.6%)	3839 (18.5%)		4188 (18.6%)
Drink 6 or more alcoholic drinks in one sitting in last 3 months (wave 5)				
Never drinks more than six drinks	1321 (74.2%)	14,636 (70.6%)	0.001	15,957 (70.9%)
Drank 6 or more drinks at least once in last 3 months	460 (25.8%)	6093 (29.4%)	<0.001	6553 (29.1%)

Equality between groups was tested using Pearson χ^2^ for categorical variables and linear regression for continuous variables.

BMI: body mass index; EURO-D: Euro depression scale.

The goldbricker test indicated no suggested reduction (i.e. less than 25% of correlations were significantly different for no pairs in the model and therefore no items were deemed redundant), and consequently all depressive items and health risk behaviours were included in the network analysis models.

### Cross-sectional networks

Cross-sectional networks for health-risk behaviours alone, depressive symptoms alone, and health-risk behaviours and depressive symptoms combined are presented separately in Supplemental Material. Additionally, bridge and cluster analyses are presented in Supplemental Material.

### Longitudinal depressive symptom networks

**Figure 2.**
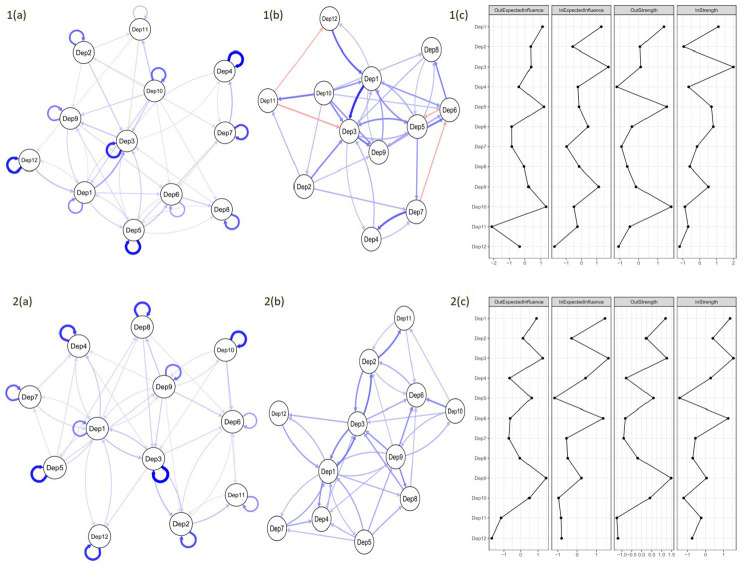
Cross-lagged panel network models of depressive symptoms between wave 4 and wave 5 for people with (top row) and people without (bottom row) diabetes. Networks are modelled with (left) and without (right) the autoregressive effects, so that the magnitude of the cross-lagged effects can be more easily interpreted. Network edges are limited to only those >0.24 in edge weight. Networks with all edges are provided in Supplemental Material. Blue edges denote positive prediction and red denote negative prediction. Thicker edges represent stronger associations. Autoregressive edges are looped arrows from one node to itself. Cross-lagged directed edges are arrows pointing to the outcome node (wave 5) from the node at wave 4. Dep1 = Depression; Dep2 = Pessimism; Dep3 = Suicidality; Dep4 = Guilt; Dep5 = Sleep; Dep6 = Interest; Dep7 = Irritability; Dep8 = Appetite; Dep9 = Fatigue; Dep10 = Concentration; Dep11 = Enjoyment; Dep12 = Tearfulness.

The CLPN for depressive symptoms for people with and without diabetes is presented in Figure 2. The strongest autoregressive effect (i.e. a node’s longitudinal relationship with itself at follow-up) for people with diabetes in the depressive network were those for Guilt (Dep4), with an edge weight (EW) of 2.09, Sleep (Dep5; EW = 1.94), and Tearfulness (Dep12; EW = 1.85) while for people without diabetes it was Suicidality (Dep3; EW = 1.99), Sleep (Dep5; EW = 1.78), and Concentration (Dep10; EW = 1.68). The strongest cross-lagged effect (i.e. a node’s longitudinal relationship with another item at follow-up) for people with diabetes was Depression (Dep1) →Suicidality (Dep3; EW = 0.81), Irritability (Dep7) → Guilt (Dep4, EW = 0.62), and Tearfulness (Dep12) → Depression (Dep1; EW = 0.61), while for people without diabetes they were Suicidality (Dep3) → Pessimism (Dep2; EW = 0.56), Pessimism (Dep2) →Enjoyment (Dep11; 0.55), and Depression (Dep1) → Suicidality (Dep3; 0.53). However, bootstrapped CIs overlapped, meaning the order of strength of these relationships is not certain.

Regarding strength and expected influence, the nodes highest in Out-EI and Out-Strength were Concentration (Dep10) and Sleep (Dep5) for people with diabetes and Fatigue (Dep9) and Suicidality (Dep3) for people without diabetes, indicating that these were the nodes that exerted the most influence on others in the network. The nodes highest in In-EI and In-Strength were Suicidality (Dep3) and Depression (Dep1) for both groups, which suggests that these were the nodes that were most influenced by others. Nodes are positioned according to the strength of their connections, which is why the network is more tightly knit after the removal of autoregressive effects.

### Longitudinal health risk behaviour and depressive symptom networks

**Figure 3.**
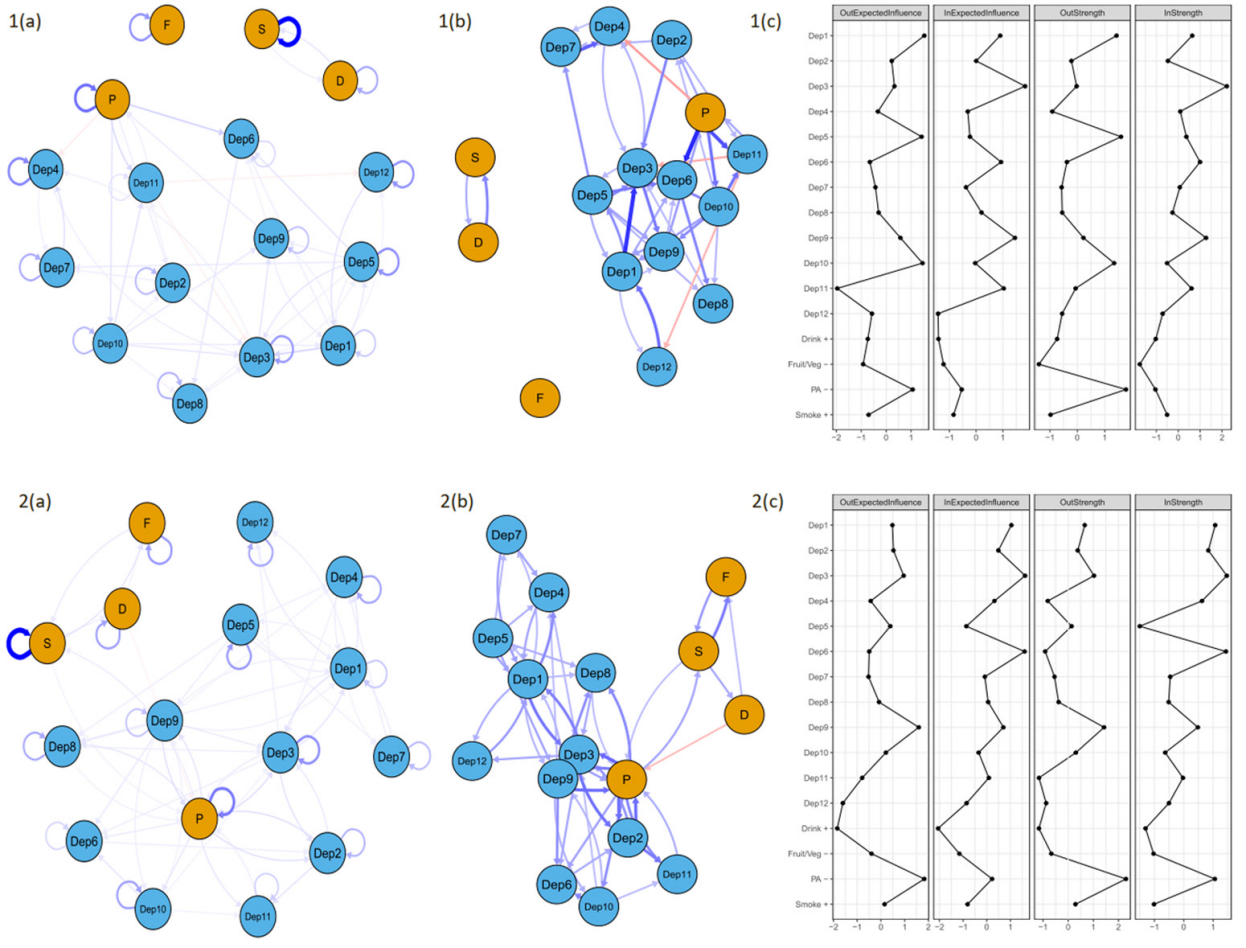
Cross-lagged panel network models of depressive symptoms and health behaviours between wave 4 and wave 5 for people with (top row) and people without (bottom row) diabetes. Presents networks with (a) and without (b) autoregressive effects and centrality indices (c). Network edges are limited to only those .0.24 in edge weight. Networks with all edges are provided in Supplemental Material. Blue edges denote positive prediction and red denote negative prediction. Thicker edges represent stronger associations. Autoregressive edges are looped arrows from one node to itself. Cross-lagged directed edges are arrows pointing to the outcome node (wave 5) from the node at wave 4. Depression symptoms are represented by blue nodes and Health risk behaviours are represented b blue nodes. Dep1 = Depression; Dep2 = Pessimism; Dep3 = Suicidality; Dep4 = Guilt; Dep5 = Sleep; Dep6 = Interest; Dep7 = Irritability; Dep8 = Appetite; Dep9 = Fatigue; Dep10 = Concentration; Dep11 = Enjoyment; Dep12 = Tearfulness; S = present smoker; P physical inactivity; F =low fruit/veg consumption; D = drank heavily at least once in last 3 months.

Figure 3 presents the CPLN for the combined network.

In the combined temporal network, there were 56.6% non-zero edges for people with diabetes and 92.2% non-zero edges for people without diabetes. For people with diabetes, the strongest autoregressive effects were for Smoking, with an EW of 4.56, Physical inactivity (EW = 2.51), and Guilt (Dep4; EW = 2.08). The strongest cross-lagged edges were Physical Inactivity → Interest (Dep6; EW = 0.83), Depression (Dep1) → Suicidality (Dep3; EW = 0.8), and Physical Inactivity →Enjoyment (Dep11; EW = 0.68). For people without diabetes, the strongest autoregressive effects were for Smoking (EW = 4.67), Physical Inactivity (EW = 2.39), and Suicidality (Dep3; EW = 1.98). The strongest cross-lagged effects were Physical Inactivity →Suicidality (Dep3; EW = 0.58) and Physical Inactivity → Pessimism (Dep2), Pessimism (Dep2) → Physical Inactivity and Fatigue Dep 9 > Physical Inactivity (all 0.56) indicating a strong reinforcing loop between Physical Inactivity and Pessimism (Dep2).

In the combined network, the nodes highest in Out-EI were Depression (Dep1) and Concentration (Dep10) and highest in Out-Strength were Physical Inactivity and Sleep (Dep5) for people with diabetes while Physical Inactivity and Fatigue (Dep9) were highest in Out-EI and Out-Strength for people without diabetes. The nodes highest in In-EI and In-Strength were Suicidality (Dep3) and Fatigue (Dep9) for people with diabetes and Interest (Dep6) and Suicidality (Dep3) for people without.

#### Network stability

CS-coefficients for centrality statistics for longitudinal combined networks were above the 0.5 cut-off to be highly stable (Epskamp et al., 2018), except for out-strength in both longitudinal networks for people with diabetes and in-strength in the depressive symptom only longitudinal network for people with diabetes (which were 0.36). While above a 0.25 cut-off to be interpretable, these statistics should nevertheless be interpreted with some caution.

## Discussion

We aimed to examine cross-sectional and longitudinal networks of depressive symptom and health risk behaviour connections in people with diabetes, compared to those without. Overall, we found that neither group showed strong connections between depressive symptoms and the health risk behaviours of smoking, low fruit/vegetable consumption, and heavy alcohol use. Physical inactivity, however, had such strong connections with depressive symptoms that it was embedded in depressive symptom clusters, rather than with the other health risk behaviours, in both groups. Contrary to our expectations, people without diabetes had more densely connected networks in both longitudinal and cross-sectional networks, with more non-zero edges. Our findings also suggest symptom-level structural differences between people with and without diabetes in terms of depressive symptom and behaviour networks. In the longitudinal networks, we observed differences between people with and without diabetes in the nodes that most strongly activate and are activated by others in the network, such as physical inactivity being less likely to be activated by other nodes in the network for people with diabetes. In other words, there were differences between groups in the role some nodes played in the network, with physical inactivity being more a driver than recipient of activation for people with diabetes. In cross-sectional networks, this included differences in clustering of nodes and in the order of importance of bridges between depressive symptoms and health behaviours. The use of several different network modelling techniques highlights the potential of a network approach to provide a ‘new way’ of examining symptom-level interactions, and in doing so, open new theoretical and practical pathways in the field of mental health and diabetes comorbidities.

We found physical inactivity was more strongly connected to depressive symptoms than other health risk behaviours in the models. Smoking, nutrition, alcohol, and physical activity related health risk behaviours tend to cluster (Noble et al., 2015), and thus we expected to see stronger connections for these behaviours with one another, with potential bridges linking to depression. Instead, we found that the physical inactivity node was pulled towards depressive symptoms due to its stronger connections with these items. The cluster analysis placed physical inactivity in depressive symptom clusters for both groups (in Supplemental Material); it was the strongest bridge in all cross-sectional networks (in Supplemental Material); and was a consistent driver of activation in longitudinal networks. There is strong evidence that physical activity is protective against depression in older adults (Cunningham et al., 2020). Interestingly, in the longitudinal CLPN for people with diabetes, we found that the ‘in’ indices, representing the extent to which a node is predicted by others, were low for physical inactivity for people with diabetes compared to the ‘out’ indices and compared to the ‘in’ indices for those without diabetes. This could suggest that physical inactivity is more likely to lead to depressive symptoms, rather than result from the presence of depressive symptoms, among people diabetes. If so, this could have important implications for clinical recommendations and understanding the presentation of depressive symptoms in people with diabetes. While there is evidence the relationship between depressive symptoms and physical activity is bidirectional, the predictive effect of depression on activity may be strongest in young adults and diminish with age (Pinto Pereira et al., 2014). Our sample with diabetes was significantly older than those without, and this may help explain why we saw physical inactivity to be less affected by other nodes in the network for this group.

Smoking, heavy episodic alcohol consumption, and less than daily consumption of fruit and vegetables had few and weak connections with depressive symptoms in all models. There may be a social element to smoking (Martin and Sayette, 2018) and drinking alcohol (Nicholson et al., 2017) which could complicate their relationship with depressive symptoms. While diet has been shown to impact depressive symptoms longitudinally for people with (Koning et al., 2023) and without (Firth et al., 2019) diabetes, it is possible that a nutrition measure focussed on fruit and vegetable consumption is too narrow to capture the scope of dietary influence (e.g. calorific content, nutrients, processed food consumption) on depressive symptoms. Sleep patterns, stress, and social factors could be considered in future dynamic systems research to better understand the interactions between health risk behaviours and depressive symptoms in people with diabetes.

This paper adopted a dynamic systems approach to investigating depression and its longitudinal connections with health behaviours and, in doing so, identified several directed relationships that may be missed using traditional research methods. The symptom of ‘feelings of depression’, unsurprisingly, had many longitudinal bidirectional relationships with other nodes for both groups. In the longitudinal depressive network, different nodes appeared to be having the most predictive effect on others for people with and without diabetes, with concentration and sleep drivers for people with diabetes while fatigue and suicidality were drivers for those without (see out-indices). For people with diabetes, sleep also had several relatively strong bidirectional relationships, whereas sleep was very low in ‘in’ indices (more than one standard deviation below the mean) for people without diabetes, implying very little activation by other nodes. This could be interpreted as sleep not being greatly impacted by the other depressive symptoms and behaviours in the network for people without diabetes, whereas for people with diabetes, it both affected and was affected by the other nodes. In the combined longitudinal network, for people with diabetes, physical inactivity strongly influenced interest and enjoyment, and for people without diabetes, it strongly influenced suicidality and engaged in a relatively strong feedback loop with pessimism. The strongest negative influence in the combined network for people with diabetes was physical inactivity on guilt (EW = −0.38), implying that physical inactivity at wave 4 was associated with reduced feelings of guilt at wave 5. A similar effect was observed in those without diabetes, albeit to a lesser extent (EW = −0.22). This finding is particularly intriguing given the relevance of guilt and perceived failure in relation to health behaviours in people with diabetes (Polonsky et al., 2005; Sebire et al., 2018). It may be that guilt surrounding physical inactivity is more prevalent in those with greater ability to engage in activity or with specific physical activity goals. Emotion regulation (Gross, 2015) and cognitive reappraisal (Clark, 2022), reflecting how individuals may cope with and process emotion and thoughts, may be important to investigate in relation to the association between physical inactivity and guilt in people with diabetes.

We hypothesised that, given the centrality of health behaviour engagement to diabetes self-care, the network would be denser and more interconnected for people with diabetes than those without. However, our findings revealed the opposite: people without diabetes had considerably more non-zero edges in the cross-sectional (35.8% vs 73.3%) and longitudinal (56.6% vs 92.2%) networks than those with diabetes. According to network theory, networks with more connections are more easily activated and reinforced by the other nodes in the network, with more strongly connected networks often indicative of mental disorder (Borsboom, 2017). This contrasts with the finding that a higher percentage of our sample with diabetes met the cut-off for likely depression according to the Euro-D scale (26.2% vs 21% for people with and without diabetes, respectively; *p* = 0.001). This discrepancy may reflect the depressive measure capturing something distinct in people with diabetes, possibly due to the high prevalence of diabetes-specific emotional issues, such as diabetes distress, in this population (Fisher et al., 2016; Perrin et al., 2017). Future research should attempt to replicate this difference in the connectivity of depressive symptoms in people with versus without diabetes.

Cluster analysis of the depressive symptom and health risk behaviour network (in Supplemental Material) confirmed the two-factor structure of the Euro-D scale identified in previous research using Factor Analysis: ‘affective suffering’ (depression, guilt, sleep, irritability, tearfulness) and ‘motivation’ (pessimism, interest, concentration, enjoyment), with suicidality, appetite, and fatigue having considerable cross-loadings (Maskileyson et al., 2021). We found the symptoms of suicidality, appetite, and fatigue varied in cluster membership between individuals with and without diabetes, aligning with affective suffering for the former and motivation for the latter. This could reflect a different pattern in how somatic symptoms (appetite and fatigue) manifest and trigger one another in people with diabetes, potentially related to the physical and emotional burden of diabetes management. Given that psychometric network theory suggests that symptoms that cluster together interact and reinforce one another (Borsboom, 2017), somatic symptoms, of potential increased importance for people with chronic physical conditions, could be important in the development and maintenance of depression for people with diabetes. Similarly, physical inactivity clustered with motivation symptoms of the Euro-D in both groups, identifying a potential bridge for physical activity and depression to activate one another (through aspects of motivation).

### Strengths and limitations

This study had several strengths. A large multinational representative sample of older adults was used. We used several state-of-the-art modelling techniques that are relatively new in mental health research and applied them to better understand depression comorbidity in diabetes. While the majority of research using a network psychometric approach utilises cross-sectional data (Guloksuz et al., 2017), we modelled longitudinal networks, which allows for theorising about temporal effects and potential causal pathways, and may provide insights into underlying mechanisms.

However, this study also had several limitations. First, we were constrained by the waves we could use from SHARE due to health behaviour questions being introduced or changed over waves. Future research should aim to model depressive networks over multiple waves in other similar ageing datasets, such as the Health and Retirement Study (HRS; Juster and Suzman, 1995) or the English Longitudinal Study of Ageing (ELSA; Steptoe et al., 2012). Second, data come from 2011 to 2013. Changes to treatment and technology options, including advances in and increased use of continuous glucose monitors, insulin delivery systems, and digital health apps, may mean this study’s sample had different treatment options to those available now. Despite these clinical advancements, we do not have a strong theoretical reason to expect there to be a major change in the strength of connections between health risk behaviours and depressive symptoms during this period, though this can be an area for future investigations. Third, the interval between waves was also constrained by the dataset and not determined by the authors. Therefore, the approximate 2-year time period between waves may be too lengthy to optimally capture the dynamic interplay between depressive symptoms and health risk behaviours over time. Changes that occur at a more micro-level would not be captured in this study and require further examination, ideally with research conducted with shorter intervals between waves. Furthermore, the timeframe does not allow clinically relevant interpretations to be drawn. As network analysis in people with diabetes continues to expand, future studies may enable the examination of networks with the specific goal of informing clinical depression management. Fourth, while the measure for heavy alcohol consumption used in the present study (≥6 drinks on one occasion) is in line with the WHO cut-off for heavy episodic drinking (60 g on one occasion; World Health Organization, 2018), some criteria for heavy episodic drinking provide different cut-offs for men and women (such as the National Institute on Alcoholism and Alcohol Abuse cut-off of 70 g vs 56 g for men and women, respectively; Rolland et al., 2017). Therefore drinking ≥6 alcoholic drinks on one occasion could be considered heavier drinking for women than for men. Fifth, while the diabetes longitudinal networks were stable enough to be interpreted, they were not highly stable on in- and out-strength for the depressive network or on out-strength for the combined network (CS-coefficient = 0.361). Therefore, these indices should be interpreted with some caution. Sixth, a major limitation of SHARE and other similar datasets is the lack of distinction between types of diabetes. Therefore, the proportion of our sample with each type of diabetes is unknown. As T1D, T2D, and gestational diabetes differ significantly in terms of clinical presentation, treatment, and progression, this lack of distinction complicates the inferences that can be made. Given that T2D makes up approximately 95% of European diabetes cases (International Diabetes Federation, 2023) we can assume the vast majority of our sample are people with T2D, and that therefore, the findings are likely most applicable to T2D. However, this is only an assumption, underscoring the need for more detailed information on people with diabetes in large-scale studies to enhance the accuracy of research findings. Future research should examine and compare networks between people with different types of diabetes. Finally, the samples with and without diabetes were significantly different from each other in terms of sample characteristics. Factors not modelled in the network, such as disability or other chronic conditions, may influence the results (e.g. by co-occurring with depressive symptoms and physical inactivity).

### Conclusions

This paper provides insights into cross-sectional and temporal dynamics regarding the influence of individual depressive symptoms and health risk behaviours on themselves and each other. Our findings suggest that physical inactivity is by far the most strongly connected health behaviour to depressive symptoms for both people with and without diabetes but may be more a driver than a consequence of depressed mood for people with diabetes. A dynamics system approach suggests there may be differing manifestations of depressive symptom relationships for people with and without diabetes. Large datasets with psychosocial variables measured in people with diabetes are needed to replicate and expand these findings.

## Supplemental Material

sj-docx-1-hpq-10.1177_13591053251334444 – Supplemental material for Physical inactivity linked to depressive symptoms in people with and without diabetes: A longitudinal network analysis of health risk behaviours and individual depressive symptoms from the Survey of Health, Ageing and Retirement in Europe Supplemental material, sj-docx-1-hpq-10.1177_13591053251334444 for Physical inactivity linked to depressive symptoms in people with and without diabetes: A longitudinal network analysis of health risk behaviours and individual depressive symptoms from the Survey of Health, Ageing and Retirement in Europe by Amy M McInerney and Sonya S Deschênes in Journal of Health Psychology
