# Supplementary material for: Physical inactivity linked to depressive symptoms in people with and without diabetes: A longitudinal network analysis of health risk behaviours and individual depressive symptoms from the Survey of Health, Ageing and Retirement in Europe
Source: J Health Psychol. 2025 Apr 25;30(14):4415–33. doi: 10.1177/13591053251334444 (PMC12678659; doi:10.1177/13591053251334444)
Supplement: sj-docx-1-hpq-10.1177_13591053251334444 – Supplemental material for Physical inactivity linked to depressive symptoms in people with and without diabetes: A longitudinal network analysis of health risk behaviours and individual depressive symptoms from the Survey of Health, Ageing and Retirement in  [file sj-docx-1-hpq-10.1177_13591053251334444.docx]

**Supplementary material**

**Section A: Cross-sectional network main results**

- Figure S1: Cross-sectional networks of depressive symptoms and health behaviours and strength and expected influence indices influence
- Figure S2: Cluster analysis and bridge statistics from the larger sample analysis.

**Section B; Sample characteristics and comparisons between included and excluded sample**

- Table S1: Sample characteristics between the included sample and those excluded from wave 4
- Table S2: Sample characteristics between the included sample and those excluded from wave 5

**Section C: Longitudinal networks additional results**

Depressive symptom network

- Figure S3: Depressive symptom networks with all edges (i.e., threshold not set at >.24 in edge weight)
- Figure S4: Edge weight confidence intervals for longitudinal depressive symptom network
- Figure S5: Case-dropping bootstrap procedure for longitudinal depressive symptom network

Combined depressive symptoms and health-risk behaviour network

- Table S3: Table of edge weights for people with diabetes
- Table S4: Table of edge weights for people without diabetes
- Figure S6: Depressive symptom and health-risk behaviour longitudinal network with all edges (i.e., not limited to only those >.24 in edge weight)
- Figure S7: Edge weight confidence intervals for longitudinal depressive symptom and health-risk behaviour network
- Figure S8: Case-dropping bootstrap procedure for longitudinal depressive symptom and health-risk behaviour network
- Table S5: Stability indices table

**Section D: Cross-sectional networks additional results**

Health behaviour networks

- Figure S9: Cross-sectional health-risk behaviour network (threshold not set at >.24 in edge weight)
- Figure S10: Strength and expected influence for cross-sectional health-risk behaviour network
- Figure S11: Edge weight confidence intervals for cross-sectional health-risk behaviour network
- Figure S12: Case-dropping bootstrap procedure for cross-sectional health-risk behaviour network

Depressive symptom networks

- Figure S13: Cross-sectional networks of depressive symptoms (threshold set at >.24 in edge weight)
- Figure S14: Cross-sectional depressive symptom networks with all edges (i.e., threshold not set at >.24 in edge weight)
- Figure S15: Strength and expected influence for cross-sectional depressive symptom network
- Figure S16: Edge weight confidence intervals for cross-sectional depressive symptom network
- Figure S17: Case-dropping bootstrap procedure for cross-sectional depressive symptom network

Combined

- Figure S18: Cross-sectional networks of depressive symptoms and health-risk behaviour with all edges (i.e., threshold not set at >.24 in edge weight)
- Figure S19: Edge weight confidence intervals for cross-sectional depressive symptom and health-risk behaviour network
- Figure S20: Case-dropping bootstrap procedure for cross-sectional depressive symptom and health-risk behaviour network
- Figure S21: Bridge indices for cross-sectional depressive symptom and health-risk behaviour network (for unstable, original sample)

Larger additional sample for bridge indices and cluster analysis

- Table S6: Sample characteristics for larger additional sample

**Section A: Cross-sectional network main results**

In the combined network, there were 35.8% non-zero nodes for people with diabetes and 73.3% non-zero edges for people without diabetes, indicating an unexpectedly more densely connected network for people without diabetes. There were few connections between current smoking, eating fruit/vegetables, or heavy episodic drinking and the depressive symptoms within each group. However, physical inactivity was strongly connected to depressive symptoms in both groups. The strongest edges in the network for both groups were those between Depression (Dep1)-Tearfulness (Dep12) and Depression (Dep1)-Suicidality (Dep3), though the CIs for Depression (Dep1)-Suicidality (Dep3) overlapped with two others for people without diabetes, and both edges’ CIs overlapped with several other edges for people with diabetes. Nodes had a similar pattern of order in terms of strength and expected influence for both groups. Figure S1 presents the cross-sectional networks, combining depressive symptoms with health risk behaviours, and centrality indices.

Figure S1: Cross-sectional networks of depressive symptoms and health behaviours (1a and 2a) and strength and expected influence indices (1b and 2b) influence for people with (top row) and people without (bottom row) diabetes. Network edges are limited to only those >.24 in edge weight.


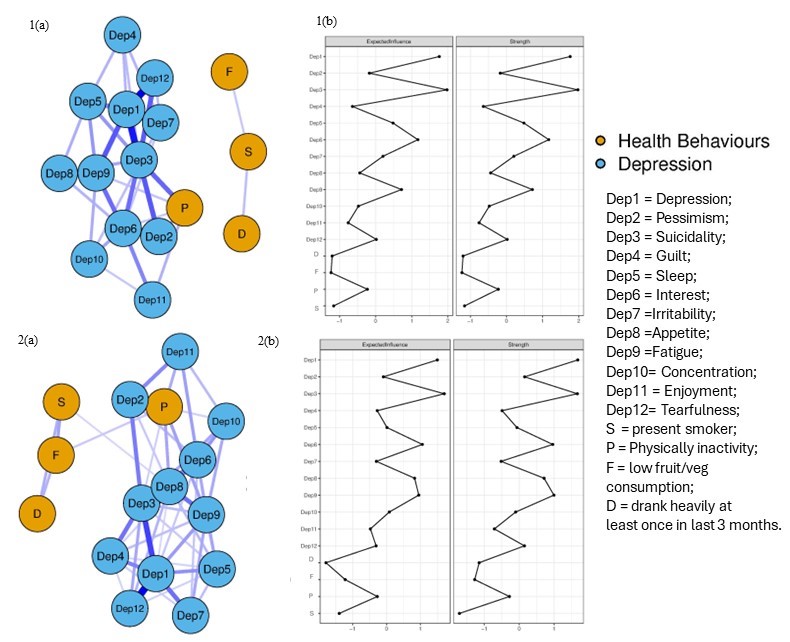


Cross-sectional bridge indices for the combined network were not stable enough to be interpreted for people with diabetes. To compare bridge indices between people with and without diabetes, we conducted bridge analysis with a more stable (larger) wave 4 sample (before the removal of those not present in both waves). Bridge statistics were highly stable (both ≥ 0.67) in the larger analysis sample. Physical inactivity remained the highest bridge as in the original, unstable analysis, but other nodes changed in order of strength and expected influence. Sample characteristics for this larger wave 4 sample are presented in Table S6.

The only difference between the original sample and larger cross-sectional analysis sample in clustering was the position of Concentration (Dep10) for people with diabetes in the combined network. Therefore, we interpreted the clustering from the more stable, larger sample. In the cluster analysis of health behaviours and depressive symptoms, there were three distinct clusters, two depressive symptom clusters and one with heavy episodic drinking, current smoking, and low fruit/vegetables consumption. Two similar, distinct clusters of depressive symptoms for people with and without diabetes were revealed. However, Suicidality (Dep3), Appetite (Dep8), and Fatigue (Dep9) were in different clusters for people with and without diabetes. The health risk behaviour of physical inactivity joined the group with Pessimism (Dep2), Interest (Dep6), Concentration (Dep10) and Enjoyment (Dep11), though for people without diabetes this cluster also contained Suicidality (Dep3), Appetite (Dep8) and Fatigue (Dep9).

Figure S2 presents the cluster analysis and bridge statistics from the larger sample analysis.

Figure S2: Cluster analysis (a) and bridge statistics (b) for people with (top row) and without (bottom row) diabetes from the larger sample analysis.
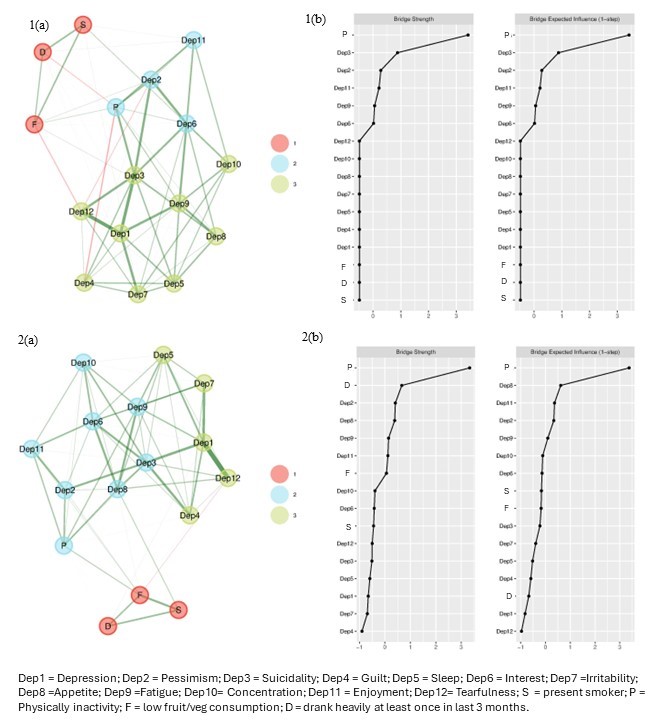


**Section B; Sample characteristics and comparisons between included and excluded sample**

Table S1: Sample characteristics between the included sample and those excluded from wave 4

|  | Wave 4 excluded sample  N (%)/M (SD) | Included sample  N (%)/M (SD) | Test |
| --- | --- | --- | --- |
| N | 35,472 (61.2%) | 22,510 (38.8%) |  |
| Diabetes |  |  |  |
| Not selected | 29,761 (84.6%) | 20,729 (92.1%) | <0.001 |
| Selected | 5,397 (15.4%) | 1,781 (7.9%) |  |
| Age in 2011 | 66.9 (10.9) | 64.4 (9.3) | <0.001 |
| Male or female |  |  |  |
| Male | 13,966 (39.4%) | 11,112 (49.4%) | <0.001 |
| Female | 21,506 (60.6%) | 11,398 (50.6%) |  |
| Marital status |  |  |  |
| Married and living together with spouse | 15,869 (64.9%) | 9,423 (68.2%) | <0.001 |
| Registered partnership | 397 (1.6%) | 236 (1.7%) |  |
| Married, living separated from spouse | 298 (1.2%) | 198 (1.4%) |  |
| Never married | 1,382 (5.7%) | 871 (6.3%) |  |
| Divorced | 2,160 (8.8%) | 1,533 (11.1%) |  |
| Widowed | 4,327 (17.7%) | 1,565 (11.3%) |  |
| Years of education | 10.035 (4.189) | 11.211 (4.409) | <0.001 |
| Number of limitations with activities of daily living |  |  |  |
| 0 | 29,644 (84.3%) | 20,995 (93.3%) | <0.001 |
| 1 | 2,530 (7.2%) | 1,035 (4.6%) |  |
| 2 | 1,159 (3.3%) | 254 (1.1%) |  |
| 3 | 636 (1.8%) | 132 (0.6%) |  |
| 4 | 399 (1.1%) | 33 (0.1%) |  |
| 5 | 350 (1.0%) | 29 (0.1%) |  |
| 6 | 442 (1.3%) | 28 (0.1%) |  |
| BMI | 27.1 (4.9) | 26.5 (4.4) | <0.001 |
| Self-perceived health - US version |  |  |  |
| Excellent | 1,619 (4.6%) | 2,261 (10.0%) | <0.001 |
| Very good | 4,330 (12.3%) | 5,004 (22.2%) |  |
| Good | 11,272 (32.0%) | 8,868 (39.4%) |  |
| Fair | 11,790 (33.5%) | 5,198 (23.1%) |  |
| Poor | 6,166 (17.5%) | 1,174 (5.2%) |  |
| EURO-D caseness |  |  |  |
| No | 21,871 (65.2%) | 17,688 (78.6%) | <0.001 |
| Yes | 11,684 (34.8%) | 4,822 (21.4%) |  |
| Present smoker |  |  |  |
| No | 28,536 (82.1%) | 17,988 (79.9%) | <0.001 |
| Yes | 6,227 (17.9%) | 4,522 (20.1%) |  |
| Physical inactivity |  |  |  |
| Other | 28,794 (82.9%) | 21,347 (94.8%) | <0.001 |
| Never vigorous nor moderate physical activity | 5,939 (17.1%) | 1,163 (5.2%) |  |
| How often serving of fruit and vegetables |  |  |  |
| Eats daily | 25,628 (73.7%) | 17,716 (78.7%) | <0.001 |
| Eats less than daily | 9,129 (26.3%) | 4,794 (21.3%) |  |
| Drink 6 or more alcoholic drinks in one sitting in last 3 months |  |  |  |
| Never drinks more than 6 drinks | 17,967 (81.1%) | 16,691 (74.1%) | <0.001 |
| Drank 6 or more drinks at least once in the last 3 months | 4,195 (18.9%) | 5,819 (25.9%) |  |
| Notes. Excluded participants are those excluded for having missing data on the variables of interest and participants who were only present at wave 4 (and not wave 5). Included and excluded participants are compared on their characteristics at wave 4. BMI =Body mass index; EURO-D = Euro depression scale; Equality between groups was tested using Pearson χ2 for categorical variables and linear regression for continuous variables. | | | |

Table S2: Sample characteristics between the included sample and those excluded from wave 5

|  | Wave 5 excluded sample  N (%)/M (SD) | Included sample  N (%)/M (SD) | Test |
| --- | --- | --- | --- |
| N | 43,528 | 22,510 |  |
| Diabetes |  |  |  |
| Not selected | 36,688 (84.7%) | 20,729 (92.1%) | <0.001 |
| Selected | 6,612 (15.3%) | 1,781 (7.9%) |  |
| Age in 2013 | 67.1 (10.8) | 66.4 (9.3) | <0.001 |
| Male or female |  |  |  |
| Male | 17,989 (41.3%) | 11,112 (49.4%) | <0.001 |
| Female | 25,539 (58.7%) | 11,398 (50.6%) |  |
| Marital status |  |  |  |
| Married and living together with spouse | 17,370 (71.7%) | 632 (52.3%) | <0.001 |
| Registered partnership | 375 (1.5%) | 23 (1.9%) |  |
| Married, living separated from spouse | 292 (1.2%) | 28 (2.3%) |  |
| Never married | 1,303 (5.4%) | 44 (3.6%) |  |
| Divorced | 1,898 (7.8%) | 108 (8.9%) |  |
| Widowed | 3,001 (12.4%) | 374 (30.9%) |  |
| Years of education | 11.6 (4.2) | 11.1 (4.6) | 0.195 |
| Number of limitations with activities of daily living |  |  |  |
| 0 | 37,382 (86.2%) | 20,756 (92.2%) | <0.001 |
| 1 | 2,566 (5.9%) | 1,134 (5.0%) |  |
| 2 | 1,180 (2.7%) | 334 (1.5%) |  |
| 3 | 703 (1.6%) | 140 (0.6%) |  |
| 4 | 480 (1.1%) | 64 (0.3%) |  |
| 5 | 398 (0.9%) | 43 (0.2%) |  |
| 6 | 636 (1.5%) | 38 (0.2%) |  |
| BMI | 26.9 (4.9) | 26.6 (4.5) | <0.001 |
| Self-perceived health - US version |  |  |  |
| Excellent | 3,385 (7.8%) | 2,068 (9.2%) | <0.001 |
| Very good | 6,840 (15.8%) | 4,719 (21.0%) |  |
| Good | 14,983 (34.6%) | 9,110 (40.5%) |  |
| Fair | 12,323 (28.4%) | 5,350 (23.8%) |  |
| Poor | 5,818 (13.4%) | 1,258 (5.6%) |  |
| EURO-D caseness |  |  |  |
| No | 29,292 (71.0%) | 17,792 (79.0%) | <0.001 |
| Yes | 11,971 (29.0%) | 4,718 (21.0%) |  |
| Present smoker |  |  |  |
| No | 7,124 (16.4%) | 4,508 (20.0%) | <0.001 |
| Yes | 36,241 (83.3%) | 18,002 (80.0%) |  |
| Physical inactivity |  |  |  |
| Other | 36,828 (85.0%) | 21,186 (94.1%) | <0.001 |
| Never vigorous nor moderate physical activity | 6,491 (15.0%) | 1,324 (5.9%) |  |
| How often serving of fruit and vegetables |  |  |  |
| Eats daily | 33,585 (77.5%) | 18,322 (81.4%) | <0.001 |
| Eats less than daily | 9,727 (22.5%) | 4,188 (18.6%) |  |
| Drink 6 or more alcoholic drinks in one sitting in last 3 months |  |  |  |
| Never drinks more than 6 drinks | 19,122 (75.1%) | 15,957 (70.9%) | <0.001 |
| Drank 6 or more drinks at least once in the last 3 months | 6,344 (24.9%) | 6,553 (29.1%) |  |
| Excluded participants are those excluded for having missing data on the variables of interest and participants who were only present at wave 5 (and not wave 4). Included and excluded participants are compared on their characteristics at wave 5. BMI =Body mass index; EURO-D = Euro depression scale; Equality between groups was tested using Pearson χ2 for categorical variables and linear regression for continuous variables. | | | |

**Section C: Longitudinal Networks additional results**

Figure S3: Depressive symptom longitudinal network with all edges (i.e., not limited to only those >.24 in edge weight) for people with (a) and without (a) diabetes. Dep1 = Depression; Dep2 = Pessimism; Dep3 = Suicidality; Dep4 = Guilt; Dep5 = Sleep; Dep6 = Interest; Dep7 =Irritability; Dep8 =Appetite; Dep9 =Fatigue; Dep10= Concentration; Dep11 = Enjoyment; Dep12= Tearfulness

1. People with diabetes


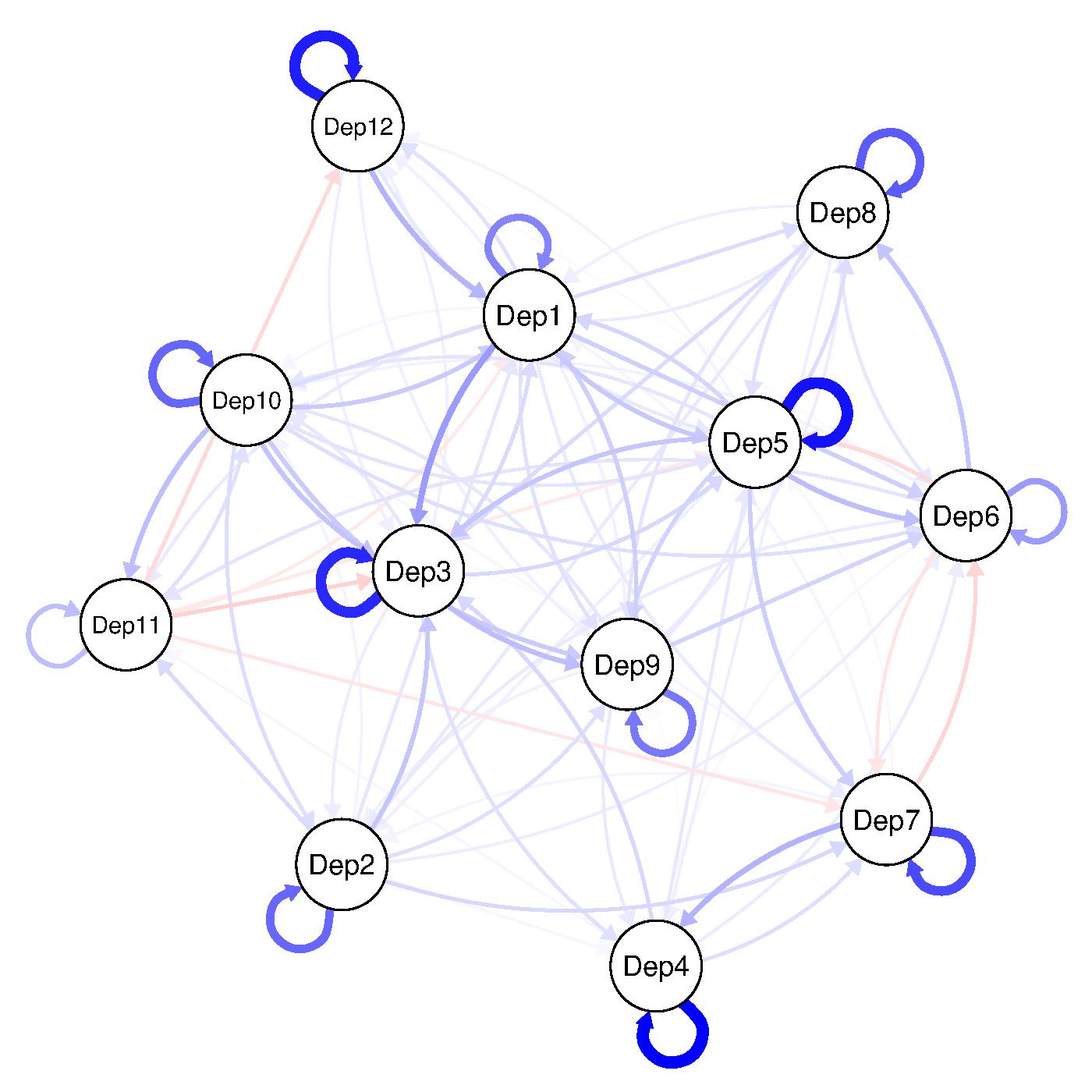


1. People without diabetes


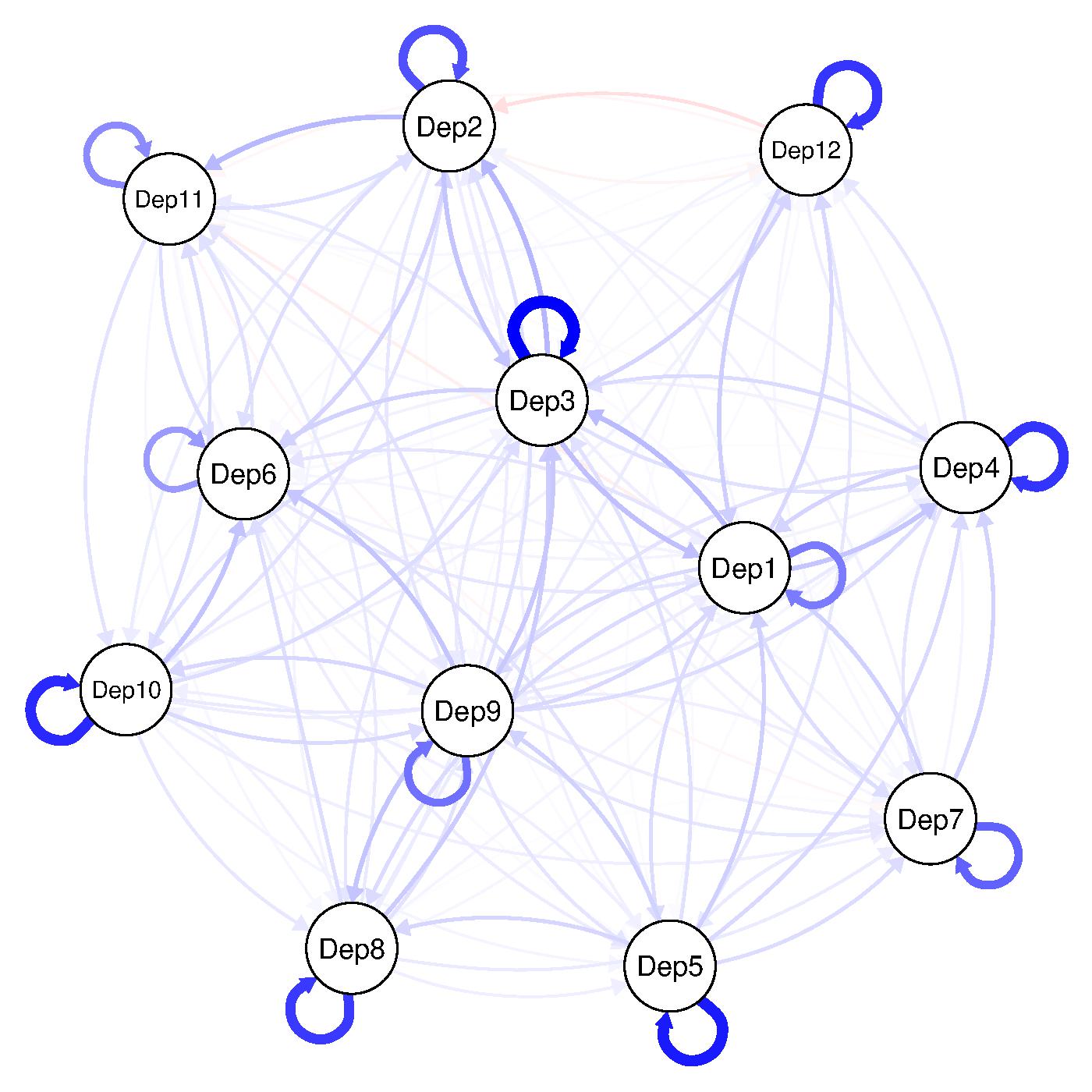


Figure S4. Bootstrapped edge weight confidence intervals for the longitudinal of network of depressive symptoms. Dep1 = Depression; Dep2 = Pessimism; Dep3 = Suicidality; Dep4 = Guilt; Dep5 = Sleep; Dep6 = Interest; Dep7 =Irritability; Dep8 =Appetite; Dep9 =Fatigue; Dep10= Concentration; Dep11 = Enjoyment; Dep12= Tearfulness.

1. People with diabetes


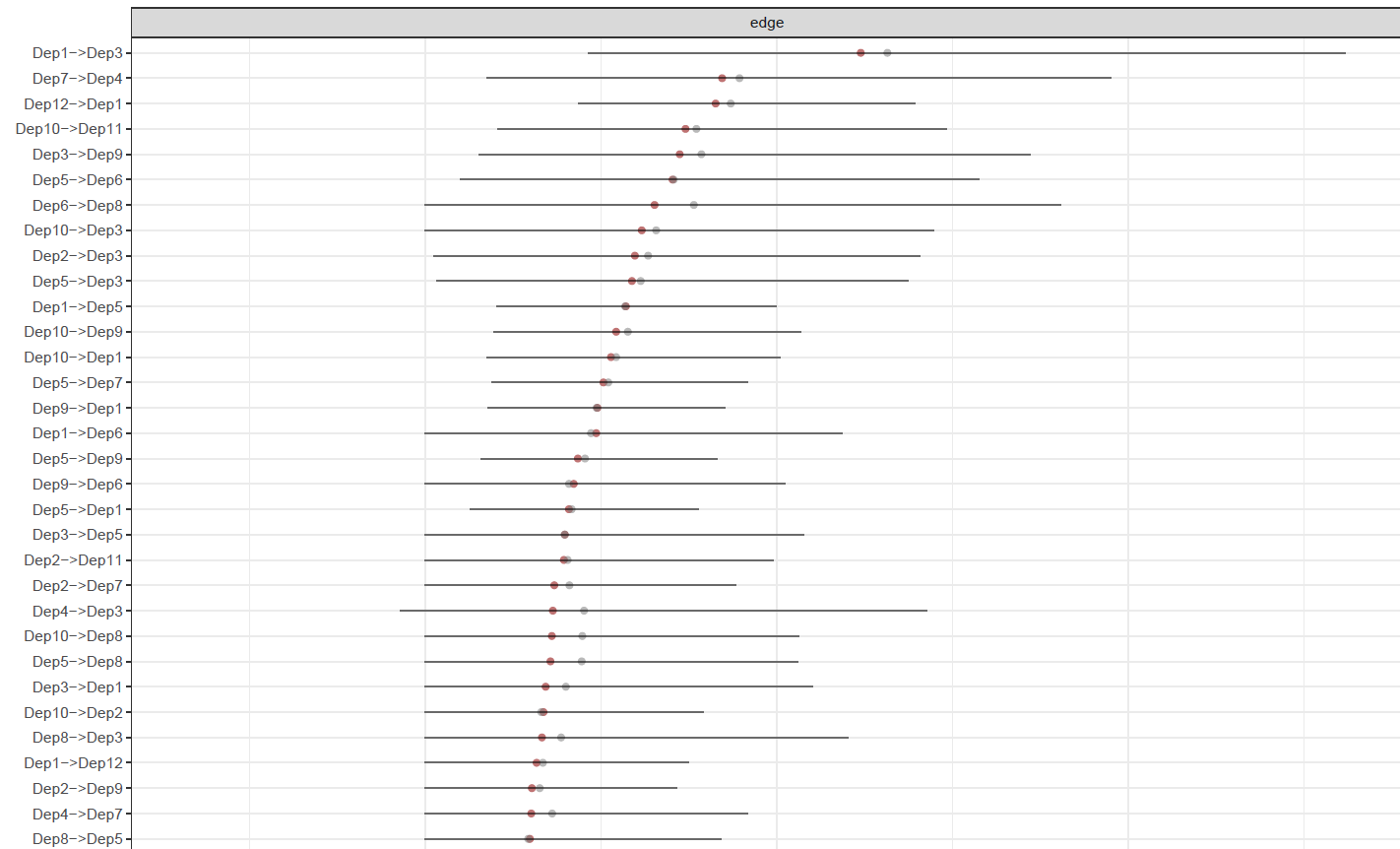

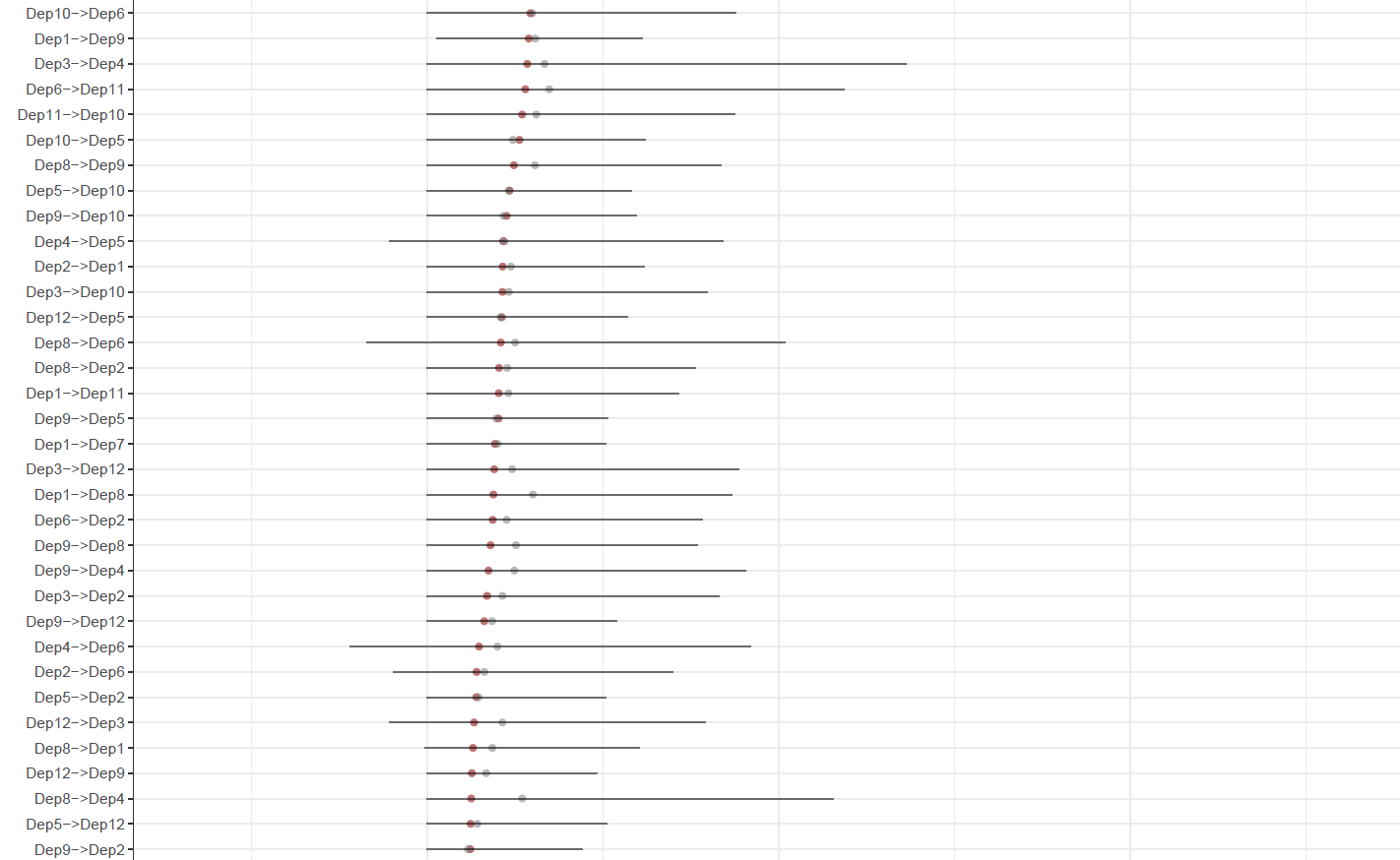

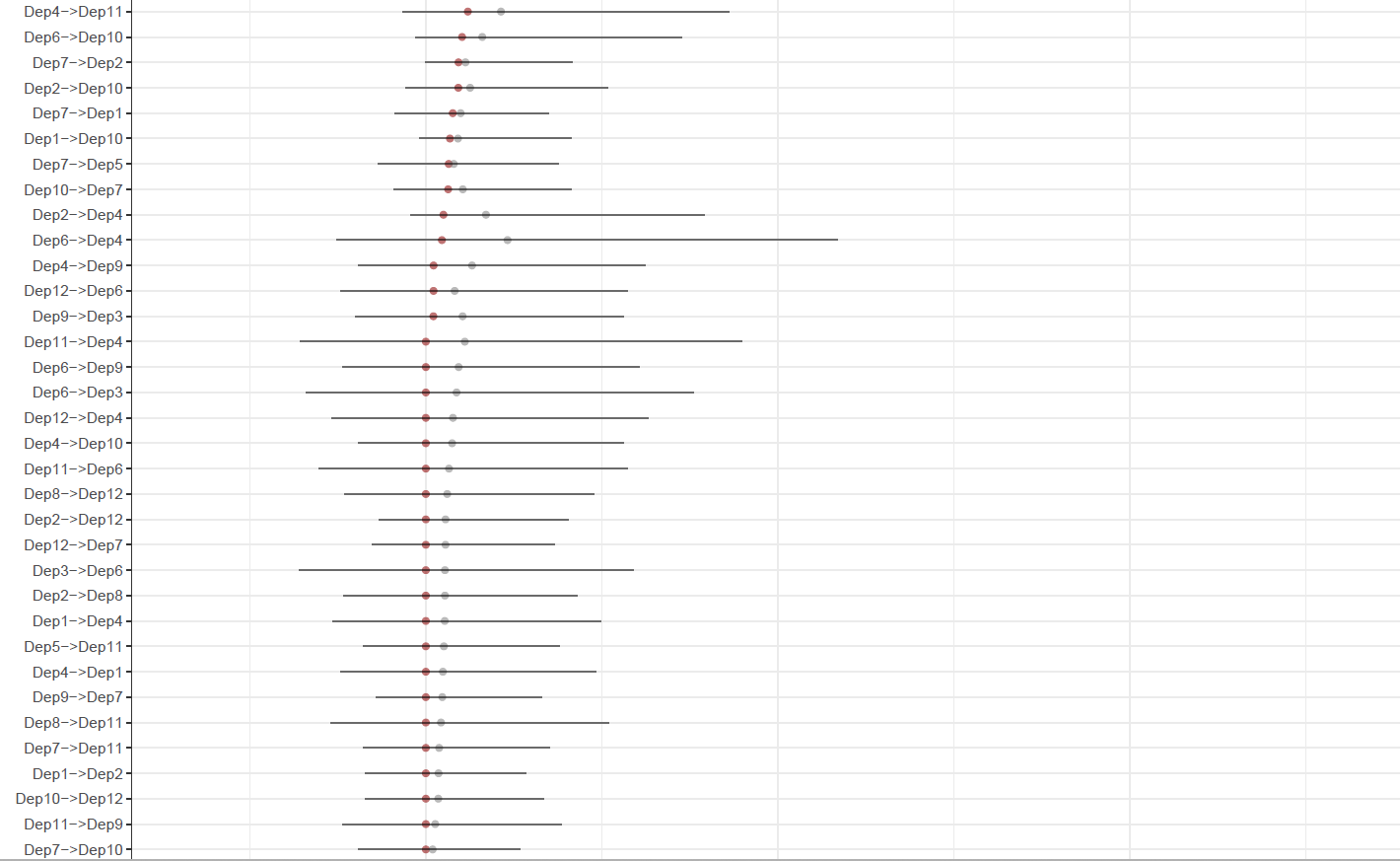

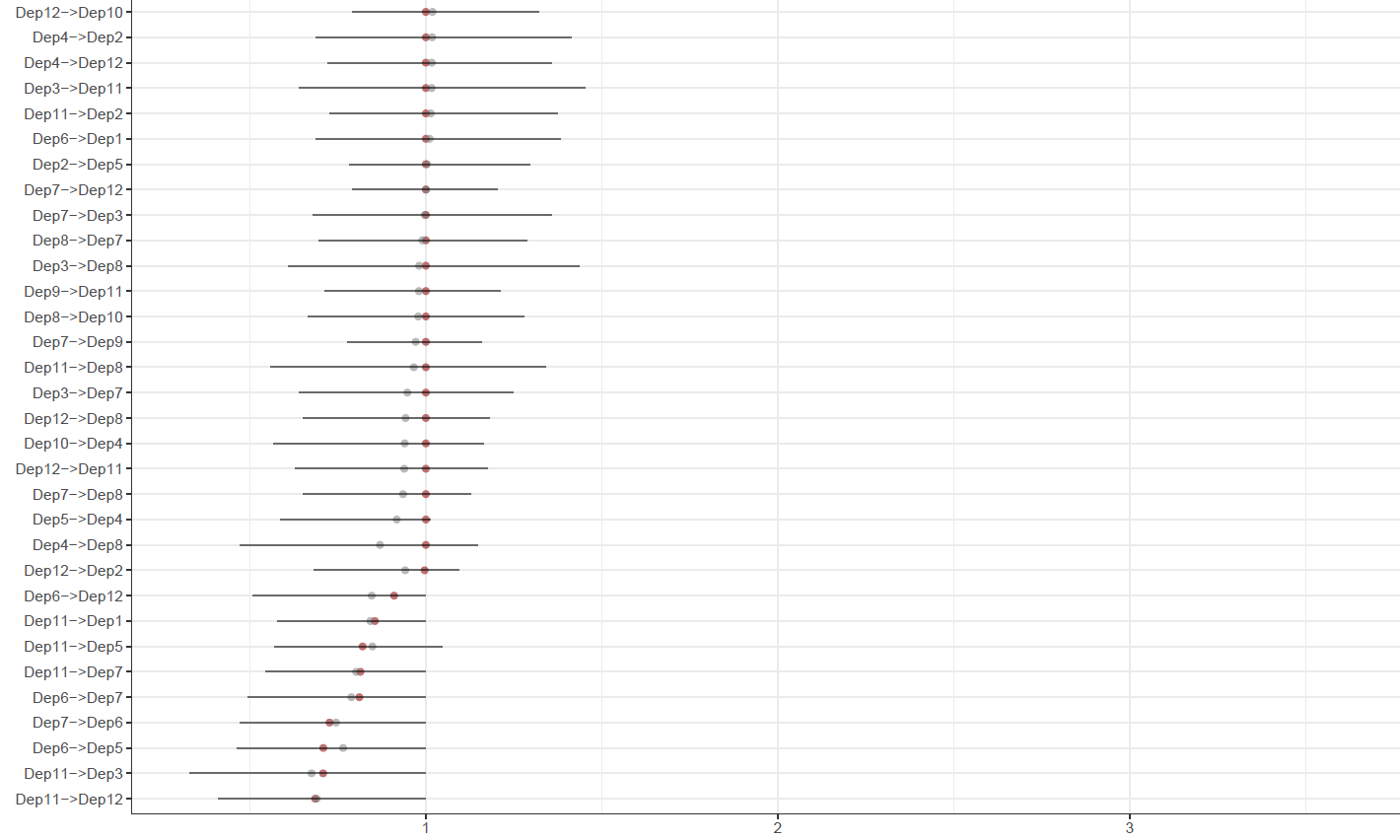


1. People with diabetes


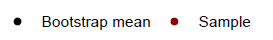


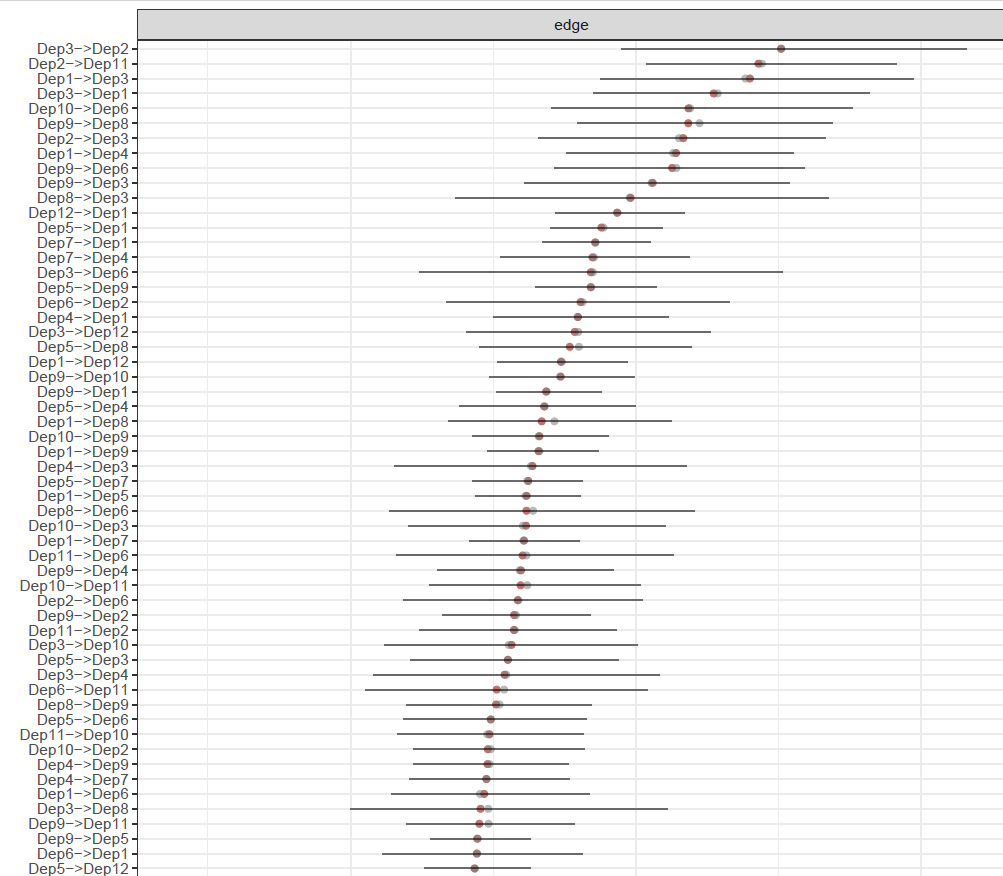

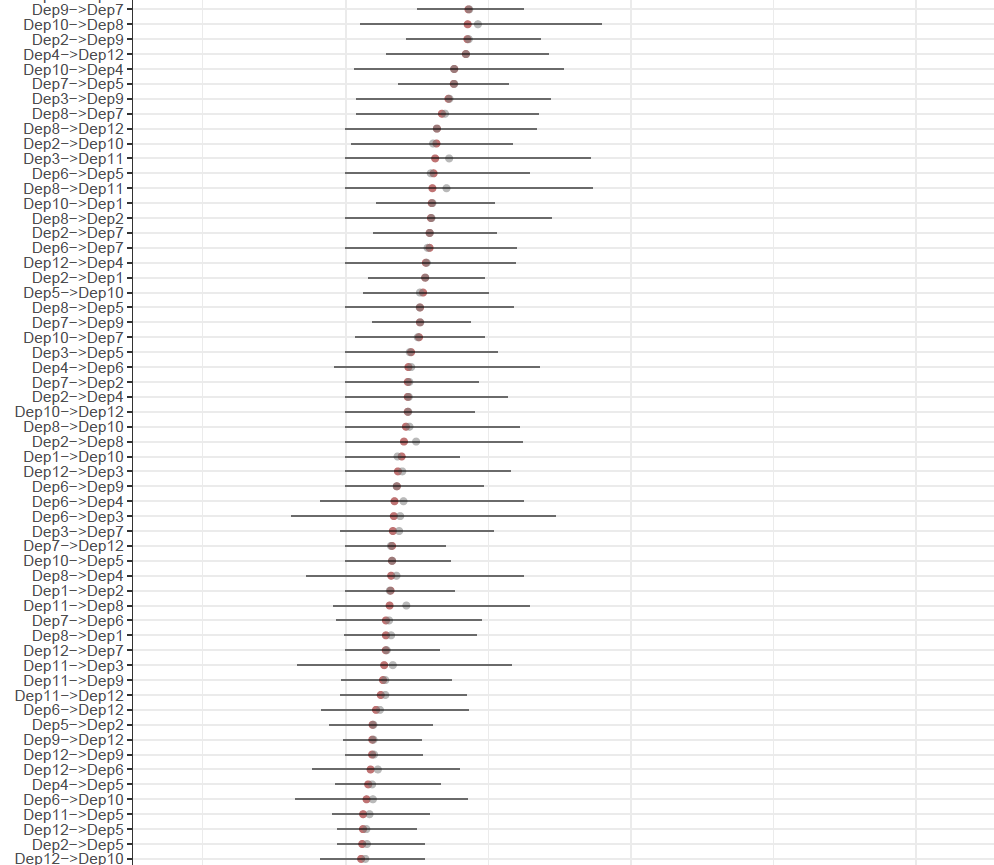


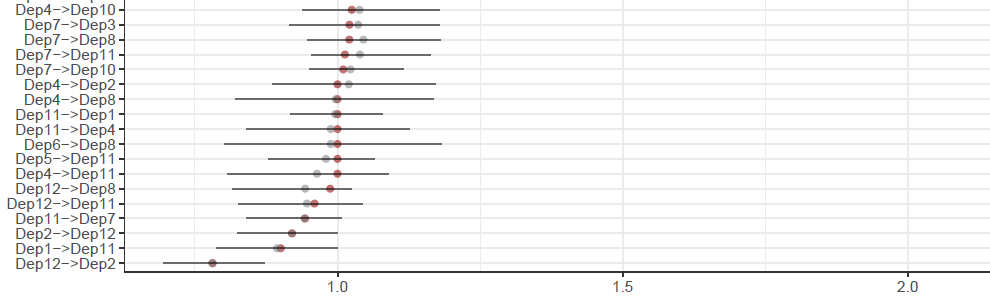


Figure S5: Average correlations between strength and expected influence statistics of the diabetes/not diabetes groups and sub-samples with different amount of cases-dropped for longitudinal depressive symptom network for people with (a) and without (b) diabetes

1. People with diabetes


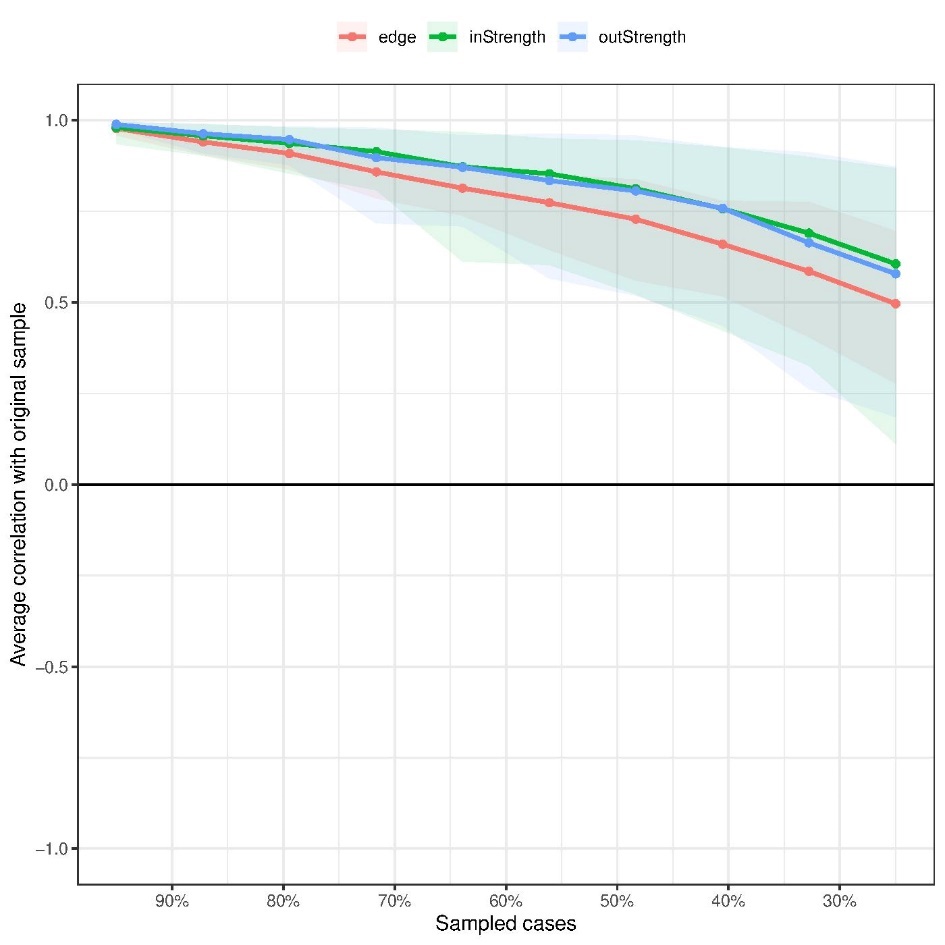


1. People without diabetes


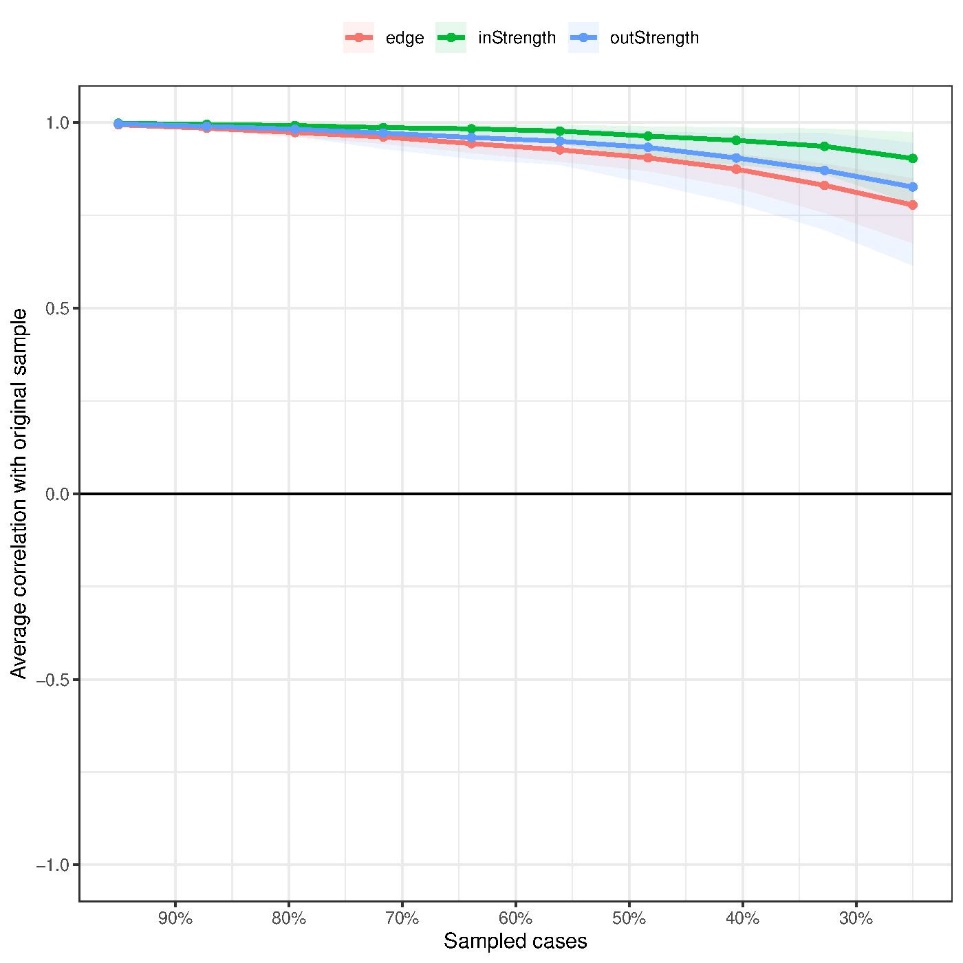


**CLPN of Depressive Symptom and health-risk behaviours**

Table S3: Edge weights from Cross-lagged panel network models of depressive symptoms and health behaviours between wave 4 and wave 5 for people with diabetes

|  | S | P | D | F | Dep1 | Dep2 | Dep3 | Dep4 | Dep5 | Dep6 | Dep7 | Dep8 | Dep9 | Dep10 | Dep11 | Dep12 |
| --- | --- | --- | --- | --- | --- | --- | --- | --- | --- | --- | --- | --- | --- | --- | --- | --- |
| S > | 4.56 |  | 0.31 | 0.13 |  |  | 0.25 |  | -0.14 | -0.02 |  | 0.07 |  |  |  |  |
| P > |  | 2.51 |  |  | 0.06 | 0.34 |  | -0.38 |  | 0.83 |  | 0.07 | 0.23 | 0.55 | 0.68 |  |
| D > | 0.47 |  | 1.51 |  | -0.03 |  | 0.1 |  |  |  | 0.05 |  | -0.09 |  | 0.22 | -0.16 |
| F > | 0.06 |  | 0.03 | 1.86 |  |  | 0.01 |  | -0.03 |  | 0.13 |  | 0.23 |  |  | -0.07 |
| Dep1 > | 0.22 |  |  |  | 0.99 |  | **0.8** |  | 0.44 | 0.34 | 0.18 | 0.15 | 0.26 | 0.05 | 0.18 | 0.25 |
| Dep2 > | 0.01 |  |  |  | 0.13 | 1.22 | 0.47 | 0.08 |  | 0.01 | 0.3 |  | 0.24 | 0.02 | 0.28 |  |
| Dep3 > |  |  | -0.02 |  | 0.24 | 0.11 | 1.76 | 0.29 | 0.27 |  |  |  | 0.52 | 0.11 |  | 0.12 |
| Dep4 > |  |  |  |  |  |  | 0.3 | 2.08 | 0.13 | 0.09 | 0.26 |  | 0.05 |  | 0.15 |  |
| Dep5 > | -0.12 | 0.11 |  |  | 0.32 | 0.13 | 0.46 |  | 1.92 | 0.51 | 0.4 | 0.29 | 0.36 | 0.2 |  | 0.09 |
| Dep6 > |  |  |  | 0.1 |  | 0.14 |  | 0.1 | -0.18 | 0.72 | -0.2 | 0.47 |  | 0.04 | 0.17 |  |
| Dep7 > | 0.16 |  | 0.11 |  | 0.05 | 0.08 |  | 0.61 | 0.04 | -0.19 | 1.48 |  |  |  |  |  |
| Dep8 > |  |  | -0.13 |  | 0.07 | 0.15 | 0.27 | 0.13 | 0.2 | 0.09 |  | 1.34 | 0.22 |  |  |  |
| Dep9 > |  | 0.38 |  |  | 0.37 | 0.09 | 0.01 | 0.19 | 0.16 | 0.27 |  | 0.15 | 1.09 | 0.16 |  | 0.12 |
| Dep10 > |  |  |  |  | 0.37 | 0.28 | 0.46 |  | 0.17 | 0.19 | 0.05 | 0.28 | 0.44 | 1.25 | 0.55 | -0.3 |
| Dep11 > | -0.21 | 0.33 |  |  | -0.05 |  | -0.32 |  | -0.1 |  | -0.2 |  |  | 0.15 | 0.51 | 1.84 |
| Dep12 > | -0.03 | 0.01 | -0.24 |  | 0.56 |  | 0.13 |  | 0.16 |  |  |  | 0.14 |  |  |  |
| Notes: Variables on the left column indicate the predictor node. Variables on the top row indicate the outcome nodes. Squares left blank indicate the edge weight was not present. Dep1 = Depression; Dep2 = Pessimism; Dep3 = Suicidality; Dep4 = Guilt; Dep5 = Sleep; Dep6 = Interest; Dep7 =Irritability; Dep8 =Appetite; Dep9 =Fatigue; Dep10= Concentration; Dep11 = Enjoyment; Dep12= Tearfulness; S = present smoker; P = Physically inactivity; F = low fruit/veg consumption; D = drank heavily at least once in last 3 months. | | | | | | | | | | | | | | | | |

Table S4: Edge weights from Cross-lagged panel network models of depressive symptoms and health behaviours between wave 4 and wave 5 for people without diabetes

|  | S | P | D | F | Dep1 | Dep2 | Dep3 | Dep4 | Dep5 | Dep6 | Dep7 | Dep8 | Dep9 | Dep10 | Dep11 | Dep12 |
| --- | --- | --- | --- | --- | --- | --- | --- | --- | --- | --- | --- | --- | --- | --- | --- | --- |
| S > | 4.67 | 0.25 | 0.33 | 0.5 | 0.02 | 0.14 | 0.24 | 0.22 | -0,06 | 0.25 | 0.03 | 0.18 | 0.08 | -0.13 | 0.17 | -0.06 |
| P > | 0.34 | 2.39 | -0.07 | 0.17 | 0.17 | 0.56 | 0.58 | -0.22 | 0.04 | 0.36 | 0.17 | 0.45 | 0.5 | 0.43 | 0.46 | 0.07 |
| D > | 0.19 | -0.26 | 1.67 | 0.26 | -0.12 | -0.03 |  | -0.05 | 0.02 |  | 0.09 |  | -0.06 | 0.05 |  | -0.15 |
| F > | 0.39 | 0.16 | 0.21 | 1.78 | 0.05 | 0.13 |  | 0.12 |  | 0.19 | 0.08 |  | 0.11 | 0.04 | 0.17 | -0.1 |
| Dep1 > | -0.08 |  | -0.05 |  | 1.03 | 0.08 | 0.53 | 0.45 | 0.27 | 0.21 | 0.27 | 0.27 | 0.28 | 0.1 | -0.12 | 0.31 |
| Dep2 > | 0.09 | 0.56 | 0.02 | 0.13 | 0.12 | 1.33 | 0.31 | 0.11 | 0.03 | 0.23 | 0.13 | 0.04 | 0.17 | 0.13 | 0.51 | -0.09 |
| Dep3 > | 0.02 | 0.37 | -0.14 | -0.02 | 0.48 | 0.55 | 1.98 | 0.24 | 0.11 | 0.34 | 0.08 | 0.18 | 0.15 | 0.24 | 0.14 | 0.34 |
| Dep4 > | -0.01 | -0.01 | 0.07 | -0.03 | 0.34 | 0.02 | 0.28 | 1.59 | 0.04 | 0.12 | 0.22 |  | 0.23 | 0.04 |  | 0.2 |
| Dep5 > | -0.04 |  |  | 0.06 | 0.36 | 0.04 | 0.23 | 0.3 | 1.78 | 0.22 | 0.27 | 0.31 | 0.35 | 0.12 |  | 0.19 |
| Dep6 > | 0.14 |  | -0.03 |  | 0.2 | 0.32 | 0.06 | 0.08 | 0.15 | 0.77 | 0.13 |  | 0.08 | 0.03 | 0.21 | 0.07 |
| Dep7 > | 0.19 | -0.2 | 0.1 | -0.03 | 0.36 | 0.11 | 0.02 | 0.35 | 0.18 | 0.07 | 1.23 |  | 0.13 | 0.02 | 0.02 | 0.09 |
| Dep8 > | 0.09 | 0.26 | -0.06 | 0.06 | 0.06 | 0.08 | 0.33 | 0.07 | 0.13 | 0.21 | 0.14 | 1.51 | 0.19 | 0.09 | 0.09 | 0.16 |
| Dep9 > | 0.05 | 0.56 | 0.04 | 0.16 | 0.29 | 0.23 | 0.39 | 0.26 | 0.2 | 0.43 | 0.19 | 0.43 | 1.09 | 0.3 | 0.19 | 0.04 |
| Dep10 > | -0.07 | 0.16 | -0.11 | -0.05 | 0.14 | 0.21 | 0.25 | 0.19 | 0.08 | 0.47 | 0.12 | 0.19 | 0.28 | 1.67 | 0.26 | 0.11 |
| Dep11 > |  | 0.31 | 0.08 |  |  | 0.21 |  |  | 0.03 | 0.22 | -0.08 |  | 0.04 | 0.2 | 0.87 | 0.07 |
| Dep12 > |  |  | -0.18 | -0.21 | 0.39 | -0.25 | 0.08 | 0.15 | 0.03 | 0.06 | 0.08 |  | 0.06 | 0.03 | -0.03 | 1.56 |
| Notes: Variables on the left column indicate the predictor node. Variables on the top row indicate the outcome nodes. Squares left blank indicate the edge weight was not present. Dep1 = Depression; Dep2 = Pessimism; Dep3 = Suicidality; Dep4 = Guilt; Dep5 = Sleep; Dep6 = Interest; Dep7 =Irritability; Dep8 =Appetite; Dep9 =Fatigue; Dep10= Concentration; Dep11 = Enjoyment; Dep12= Tearfulness; S = present smoker; P = Physically inactivity; F = low fruit/veg consumption; D = drank heavily at least once in last 3 months. | | | | | | | | | | | | | | | | |

Figure S6: Depressive symptom and health-risk behaviour longitudinal network with all edges (i.e., not limited to only those >.24 in edge weight) for people with (a) and without (a) diabetes

1. With diabetes


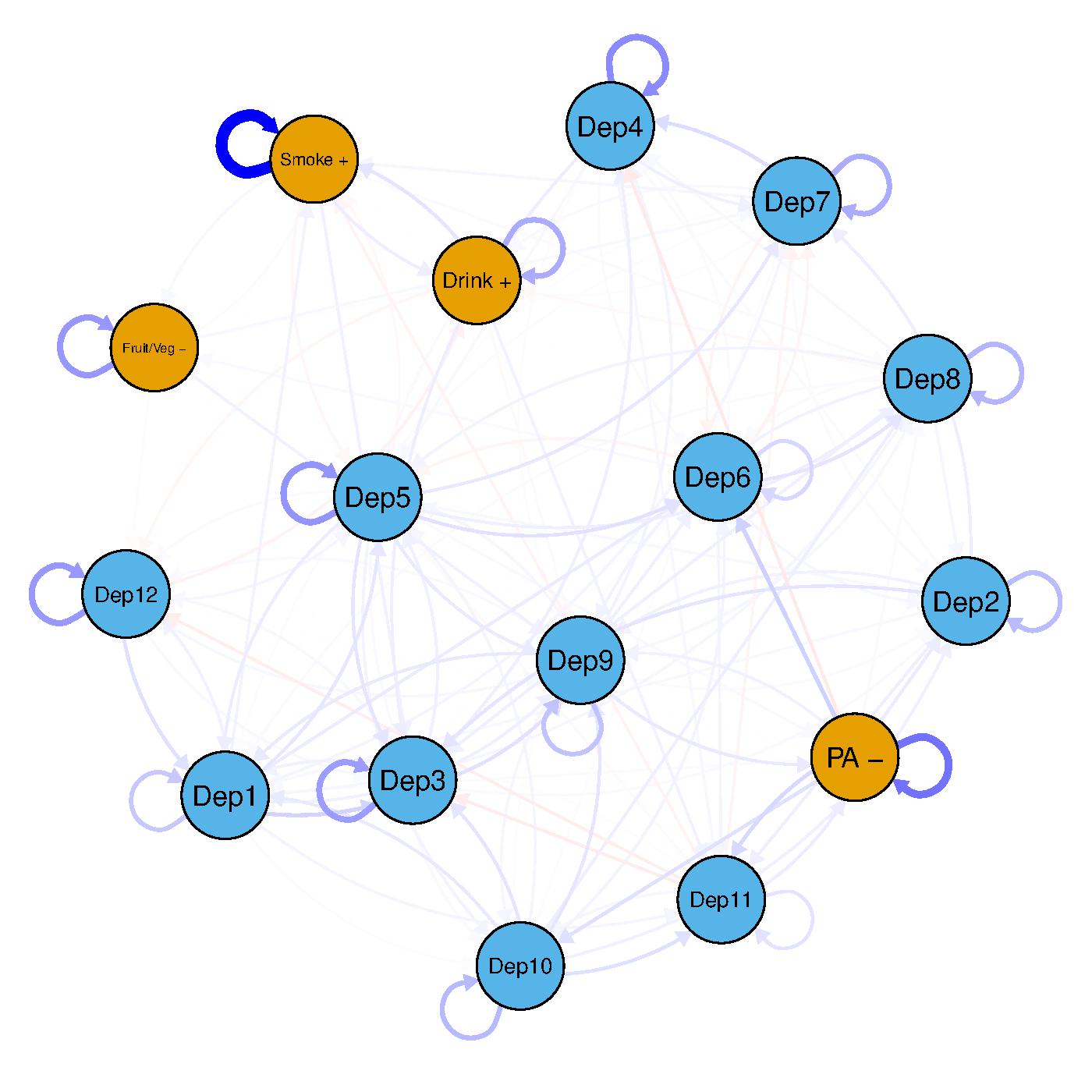


1. Without diabetes


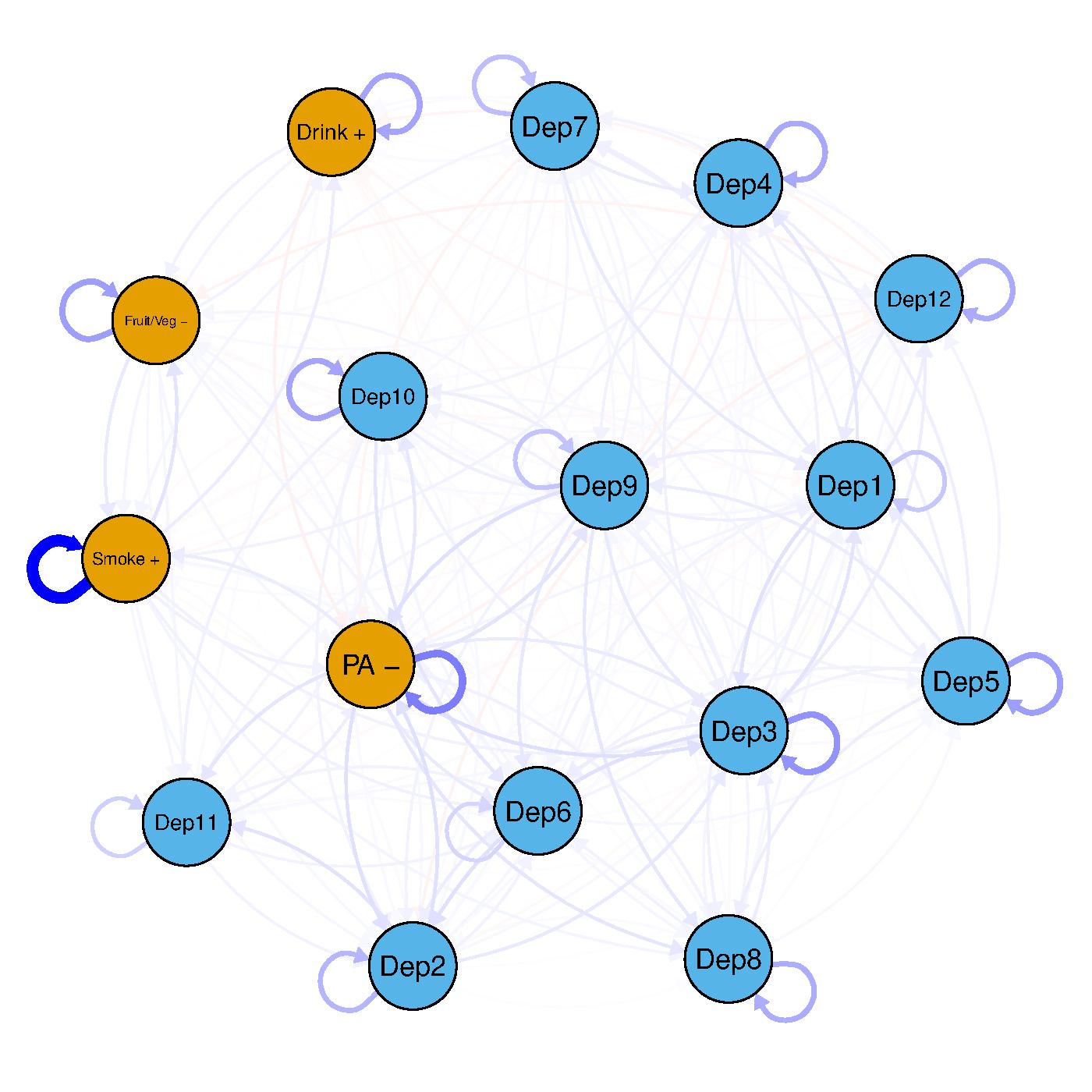


Figure S7. Bootstrapped edge weight confidence intervals for the longitudinal of combined network

1. People with diabetes


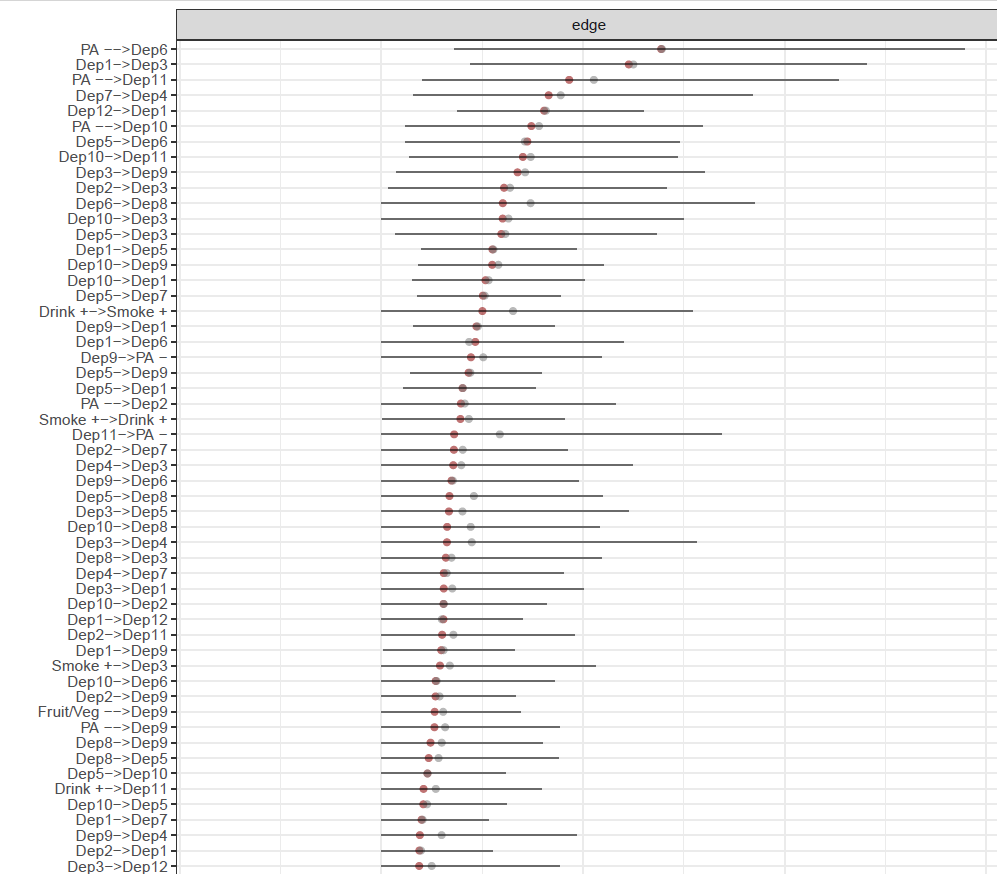

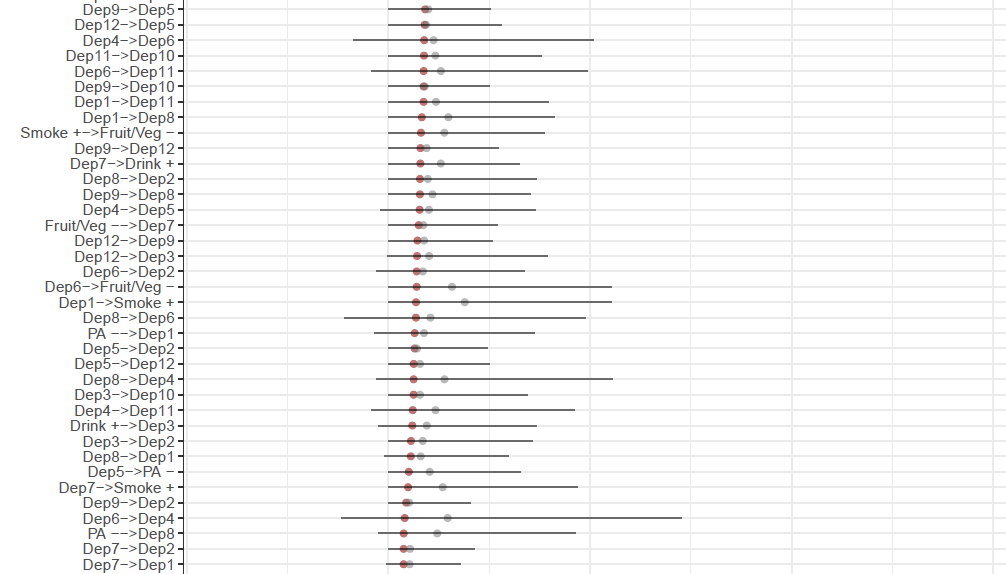

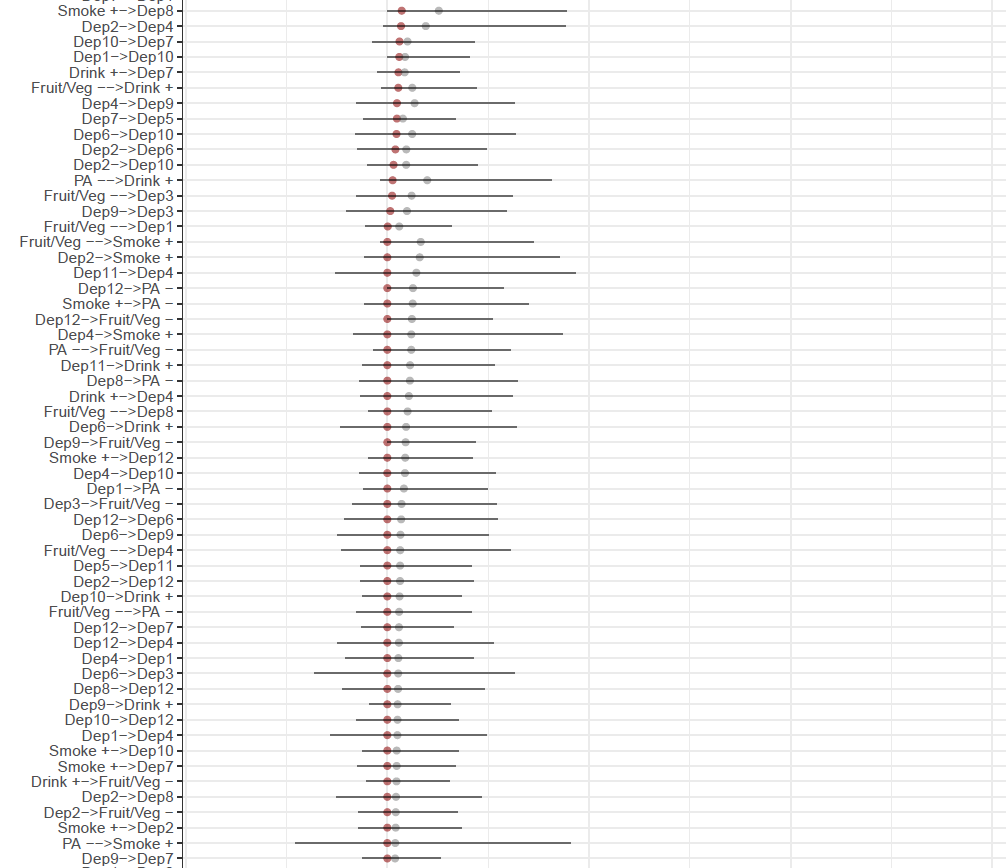

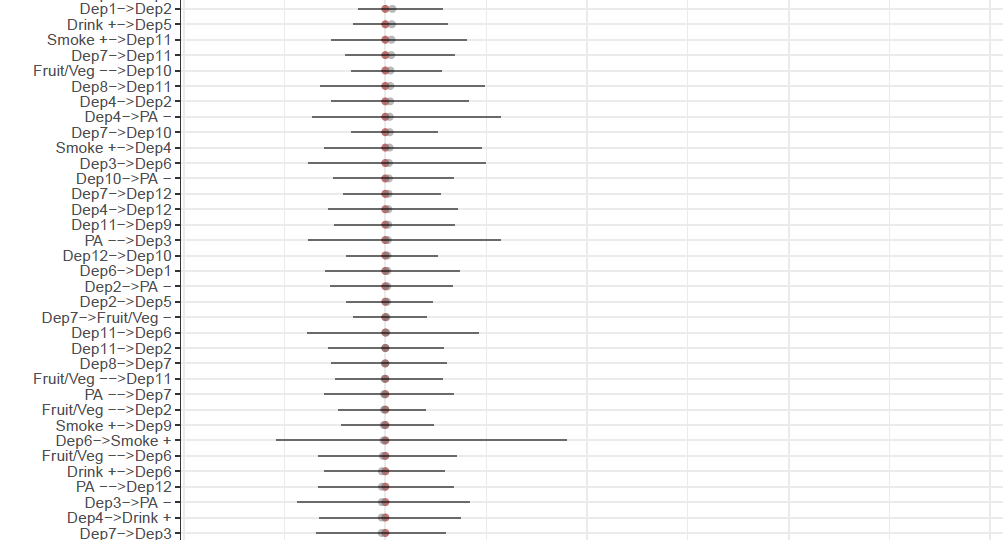

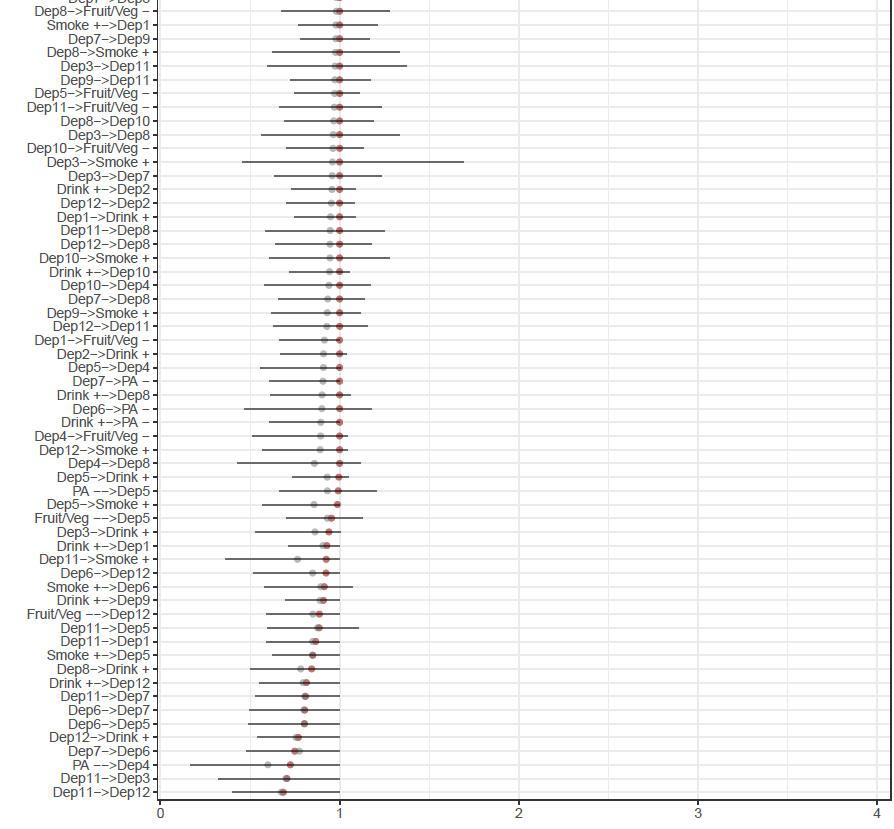


1. People without diabetes


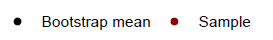


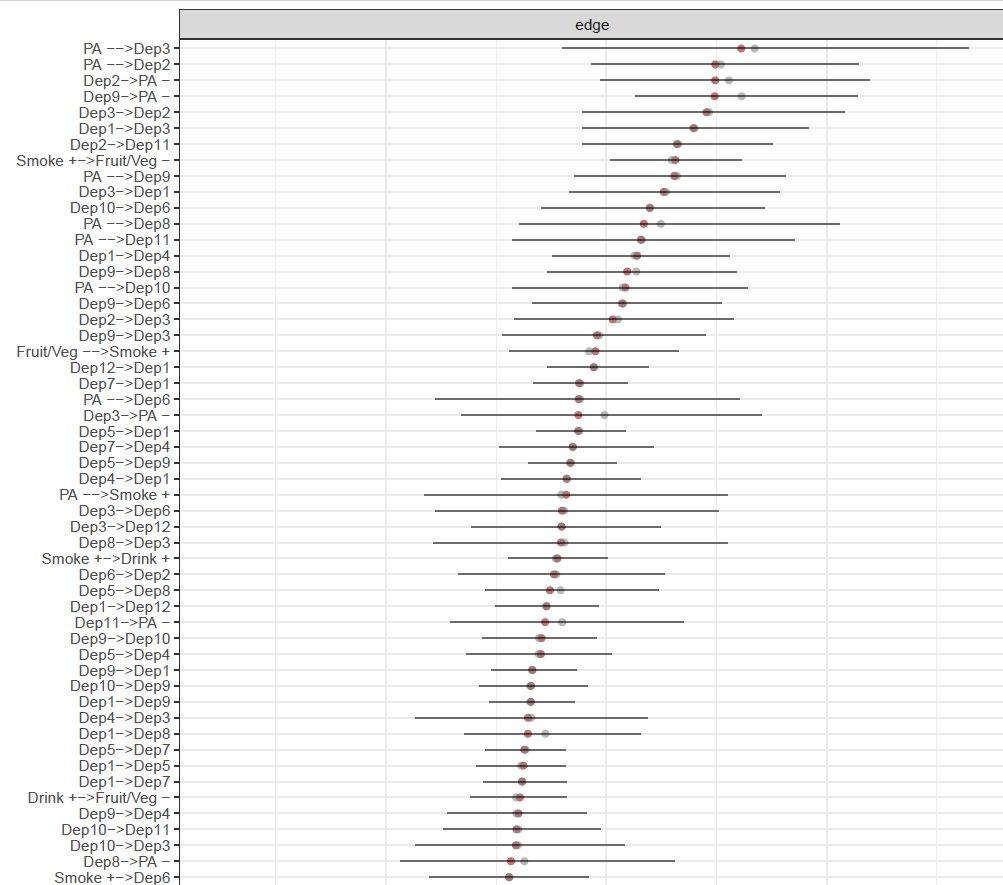

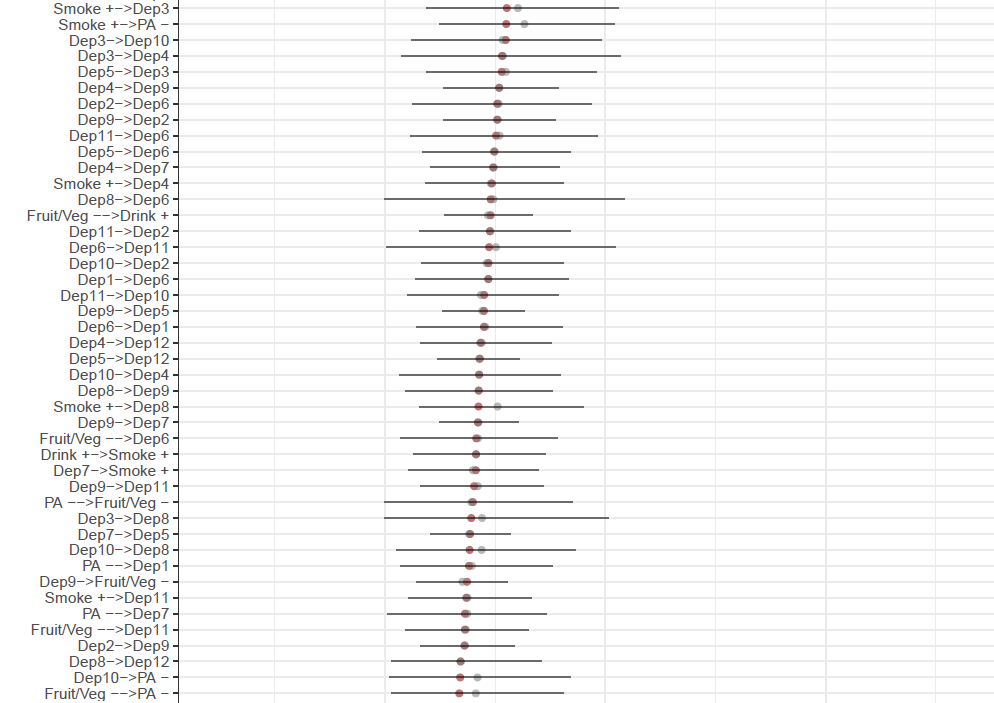


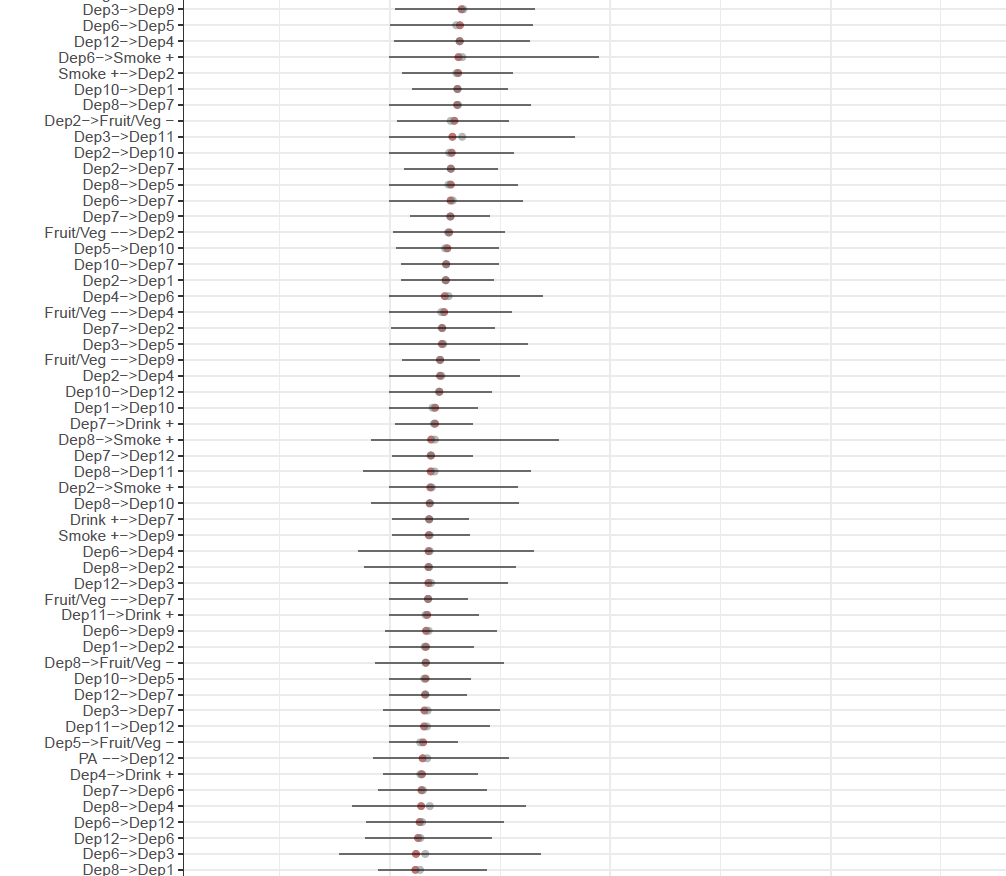

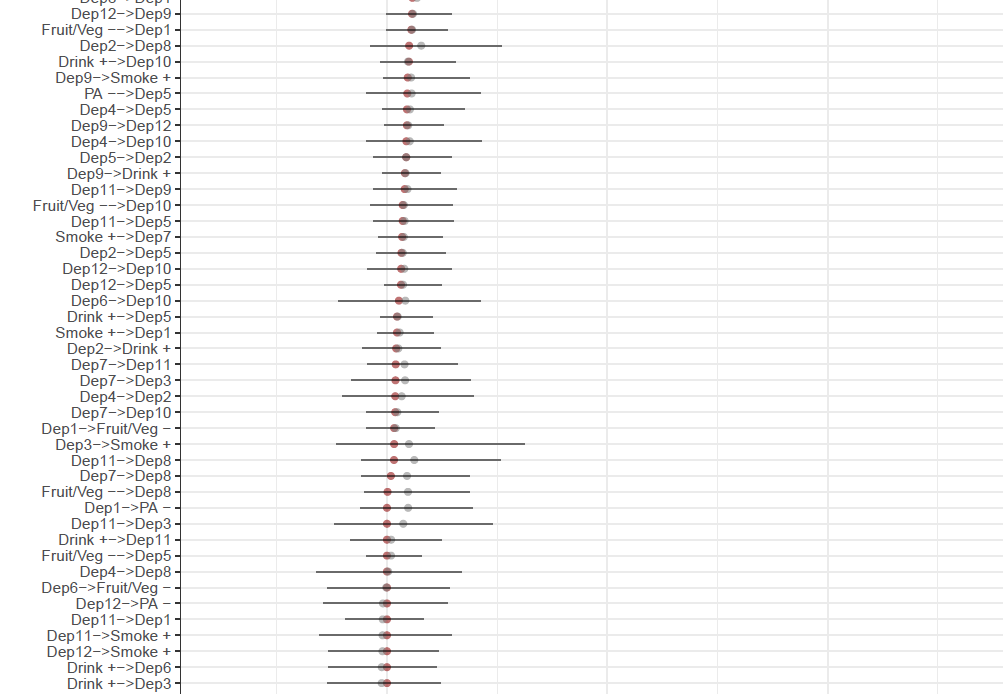

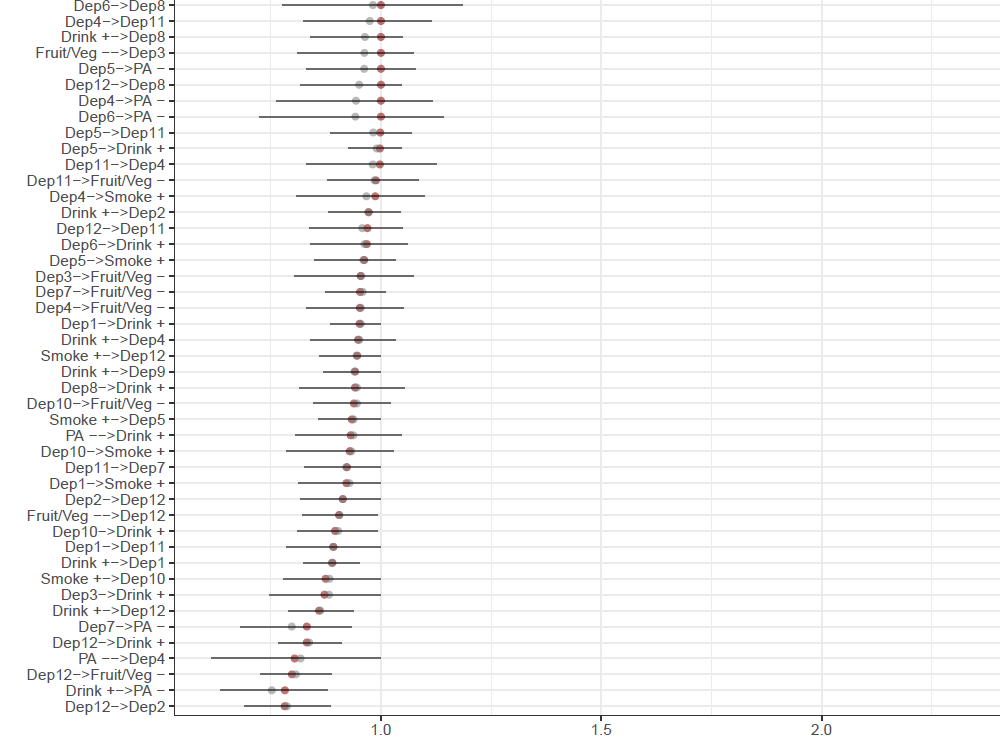


Figure S8: Average correlations between strength and expected influence statistics of the diabetes/not diabetes groups and sub-samples with different amount of cases-dropped for longitudinal depressive symptom and health-risk behaviour network for people with (a) and without (b) diabetes

1. People with diabetes


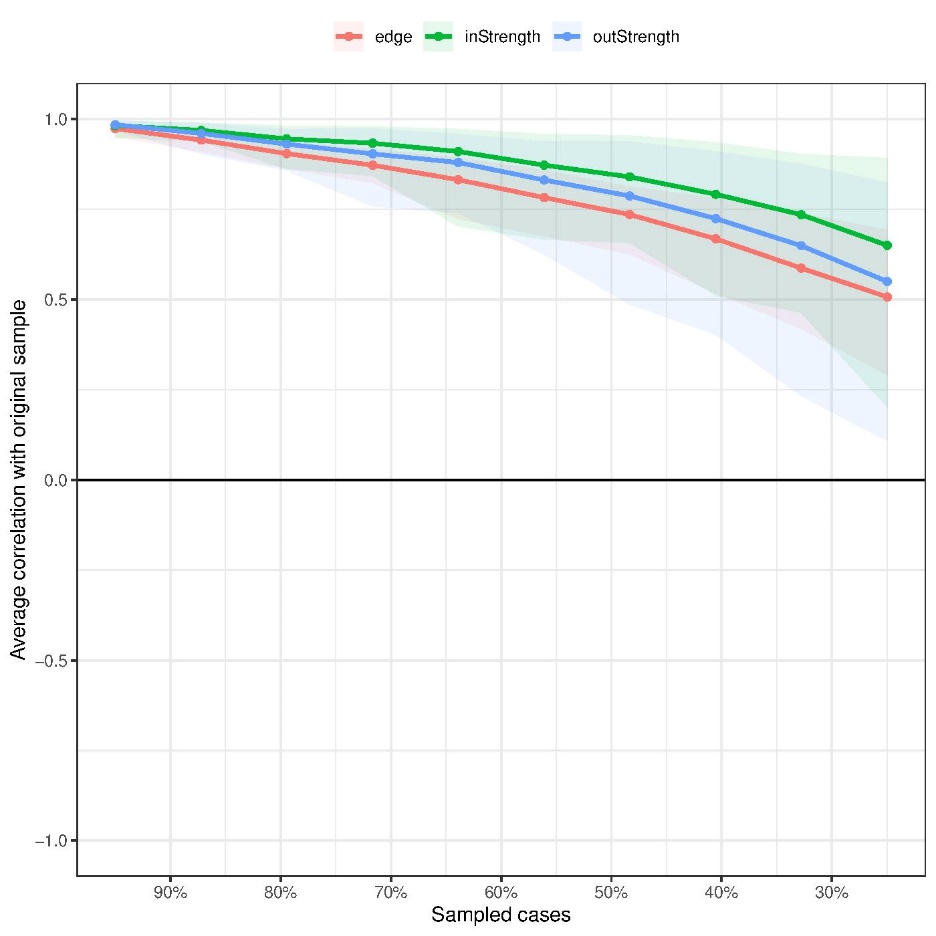


1. People without diabetes


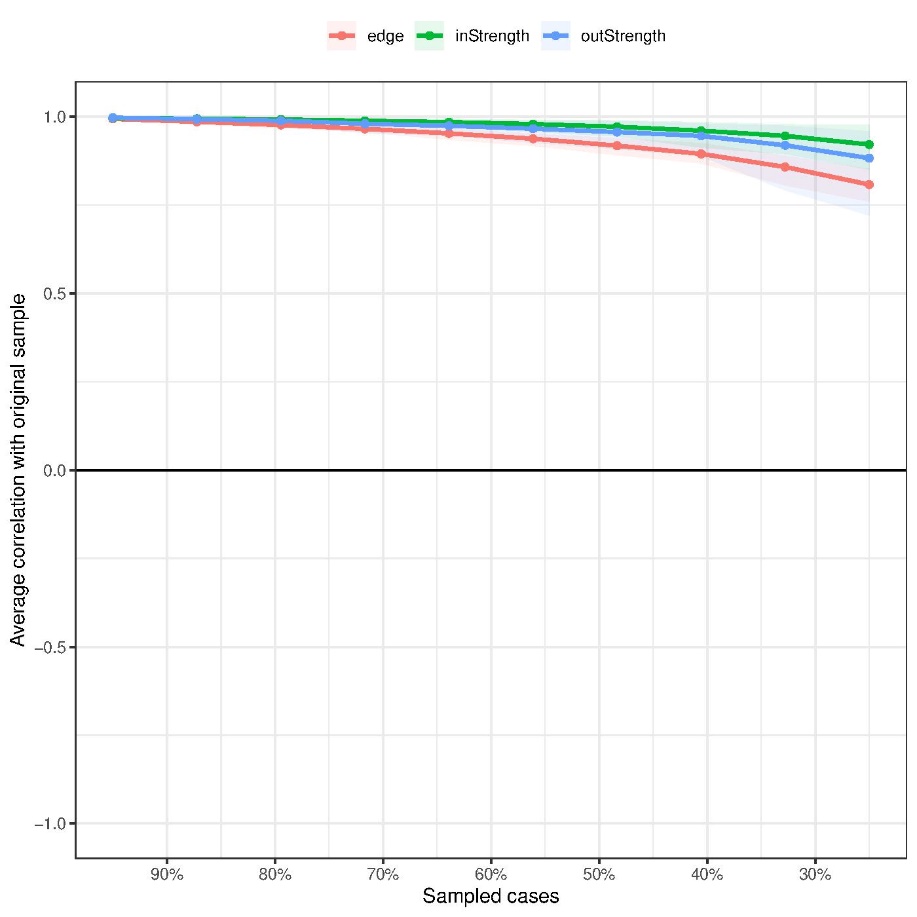


Table S5: Network stability indices for all cross-sectional and longitudinal networks for people with and without diabetes

|  | Diabetes | Without diabetes |
| --- | --- | --- |
| Cross-sectional health-risk behaviour network | Strength: 0.05  Expected Influence: 0.05 | Strength: 0.75  Expected Influence: 0.75 |
| Cross-sectional depressive symptom network | Strength: 0.517  Expected Influence: 0.439 | Strength: 0.75  Expected Influence: 0.75 |
| Cross-sectional health-risk behaviour and depressive symptom network | Strength: 0.595  Expected Influence: 0.595  Bridge Strength: 0.128  Bridge Expected Influence: 0.128 | Strength: 0.75  Expected Influence: 0.75  Bridge Strength: 0.75  Bridge expected influence: 0.75 |
| Longitudinal depressive symptom network | In-Strength: 0.361  Out-Strength: 0.361 | In-Strength: 0.75  Out-Strength: 0.672 |
| Longitudinal health-risk behaviour and depressive symptom network | In-Strength: 0.517  Out-Strength: 0.361 | In-Strength: 0.75  Out-Strength: 0.75 |
|  |  |  |
| Additional analysis |  |  |
| Cross-sectional health-risk behaviour and depressive symptom network | Strength: 0.75  Expected Influence: 0.75  Bridge Expected Influence: 0.672  Bridge Strength: 0.672 | Strength: 0.75  Expected Influence: 0.75  Bridge Expected Influence: 0.75  Bridge Strength: 0.75 |
| Note: CS-coefficient should be over 0.25 to be interpretable, over 0.5 to be highly stable. | | |

**Section D: Cross-sectional networks additional results**

Figure S9: Cross-sectional networks of health behaviours for people with (a) and without diabetes (b). Circles represent the variables of interest. Lines between nodes represent the associations between them. Blue denotes a positive association, red denotes a positive. The thicker the link the stronger the association. Network was the same with and without a threshold limiting edges to >.24 in edge weight, so only one network is presented. Smoke + = present smoker; PA - = Physically inactivity; Fruit/veg -; doesn’t eat fruit/veg daily; Drink + = drank heavily at least once in last 3 months.

1. People with diabetes


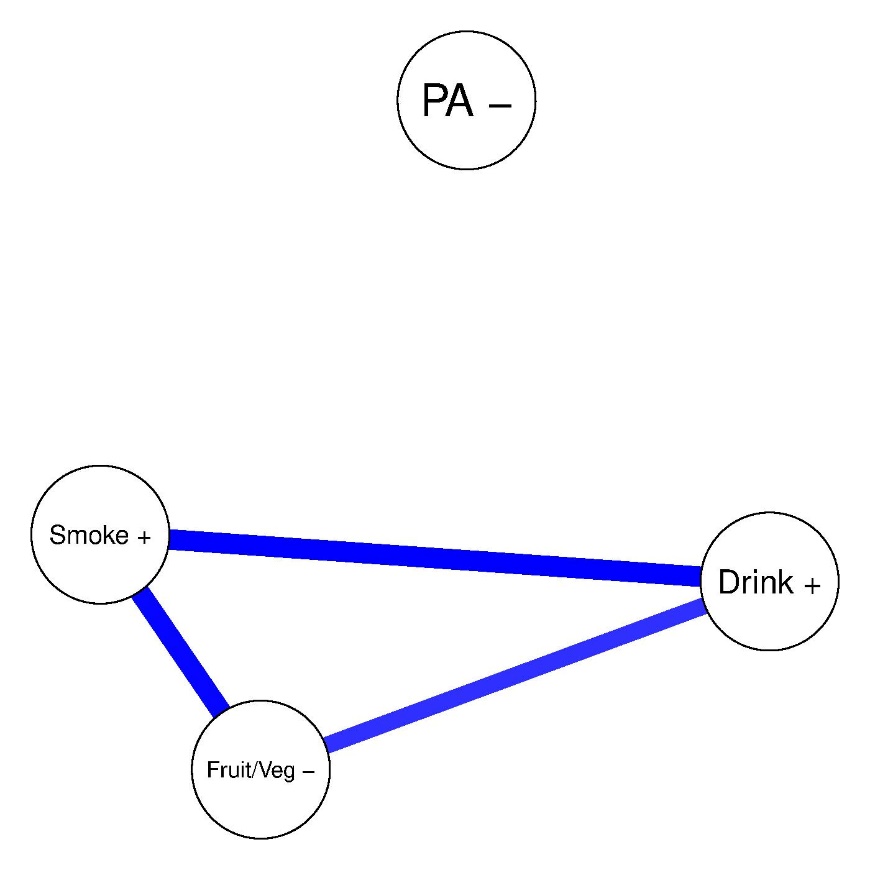


1. People without diabetes


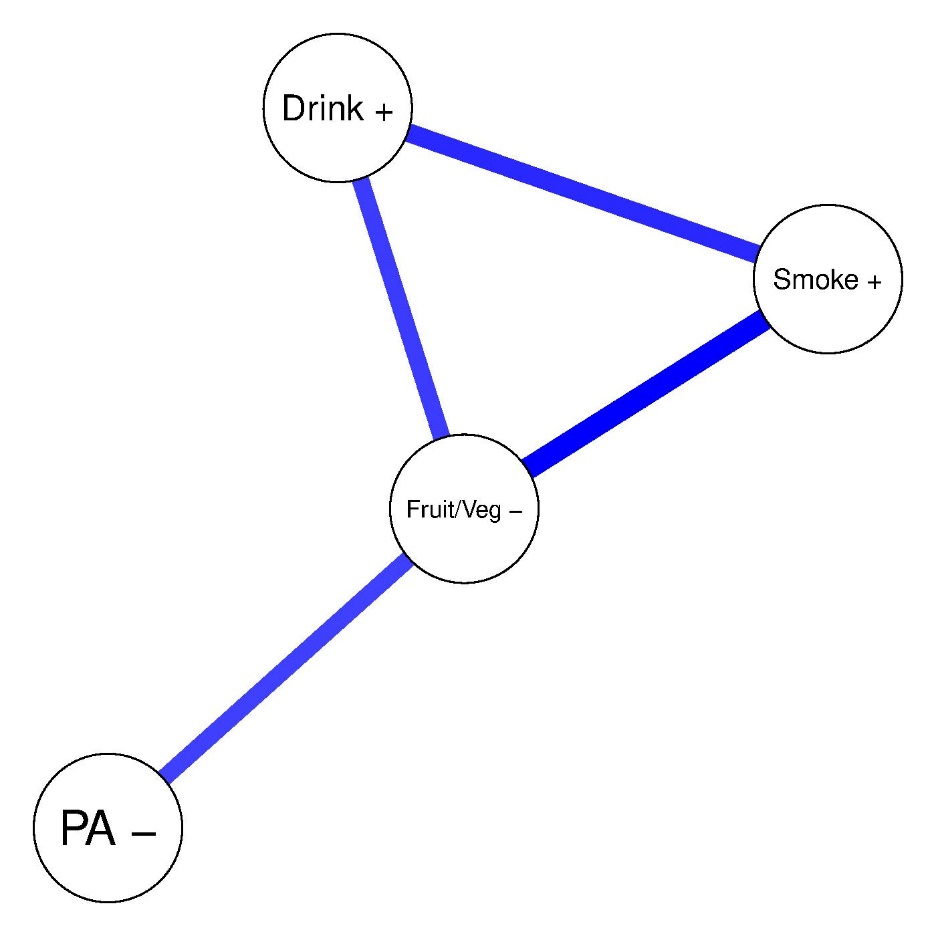


Figure S10: Cross-sectional networks of expected influence and strength for health behaviour network for people with (a) and without (b) diabetes. Strength presented as z-scores. Smoke + = present smoker; PA - = Physically inactivity; Fruit/veg -; doesn’t eat fruit/veg daily; Drink + = drank heavily at least once in last 3 months.

1. People with diabetes


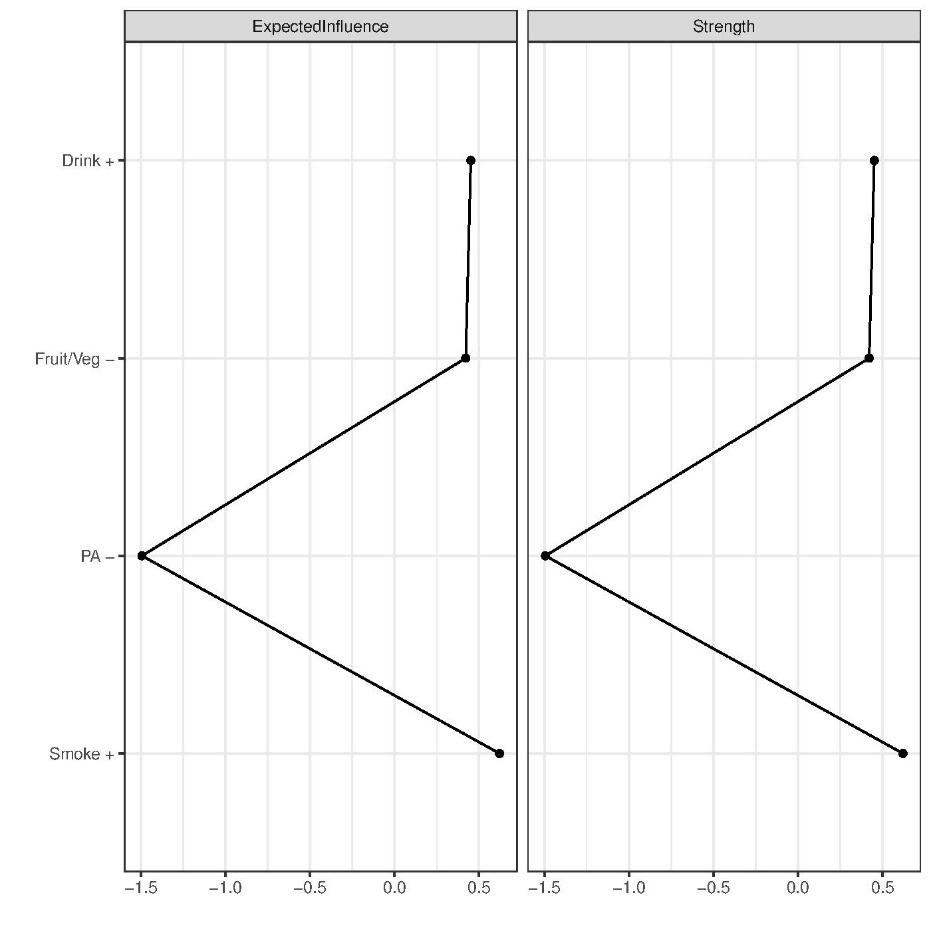


1. People without diabetes


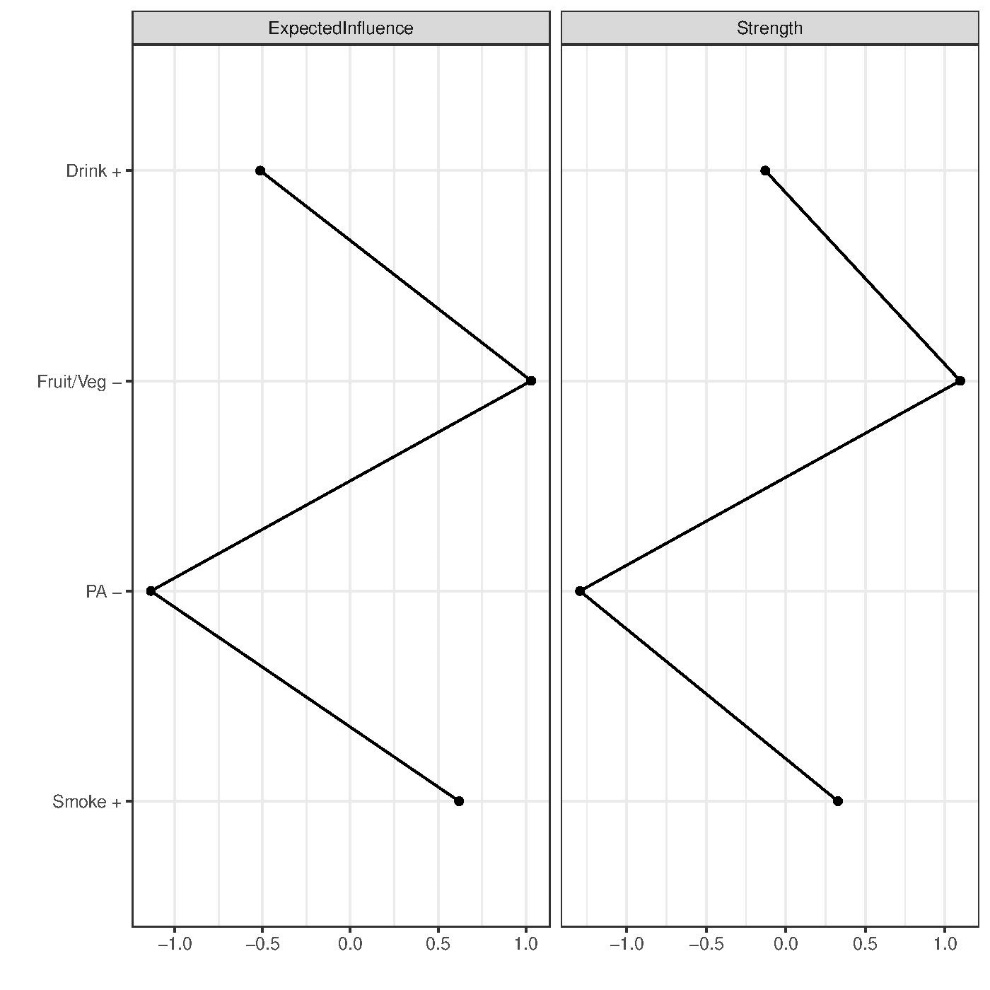


Figure S11: Bootstrapped edge weight confidence intervals for the cross-sectional network of health risk behaviours for people with (a) and without (b) diabetes. Edge weights for each edge (connection between nodes) with 95% Confidence Intervals. Smoke + = present smoker; PA - = Physically inactivity; Fruit/veg -; doesn’t eat fruit/veg daily; Drink + = drank heavily at least once in last 3 months.

1. People with diabetes


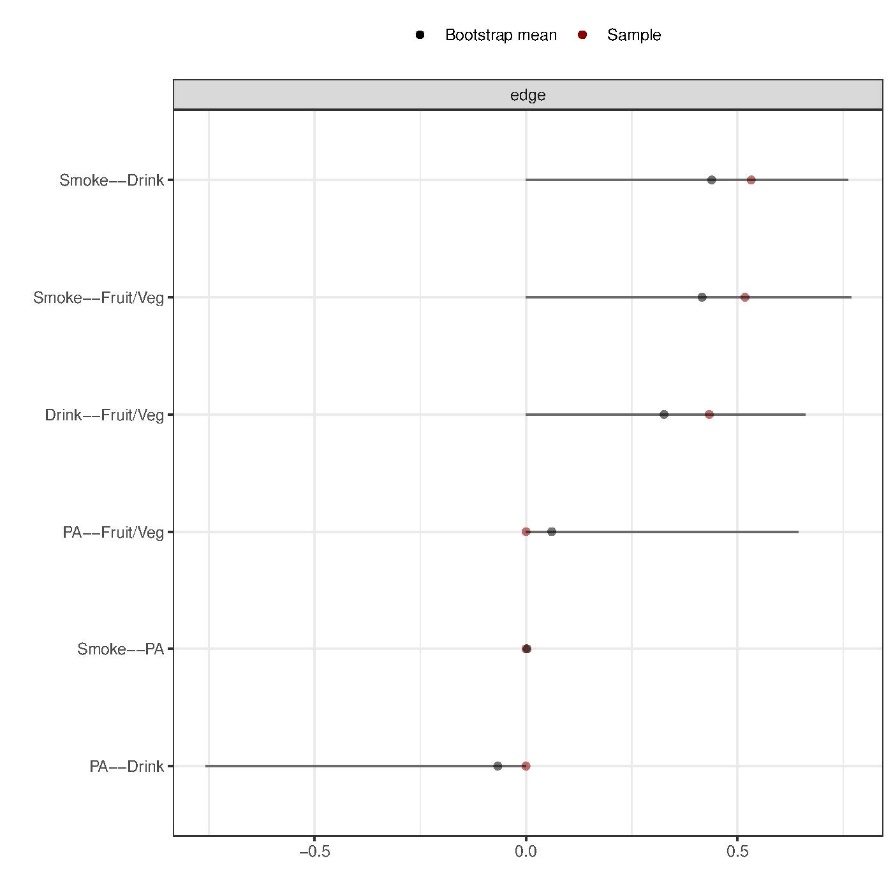


1. People without diabetes


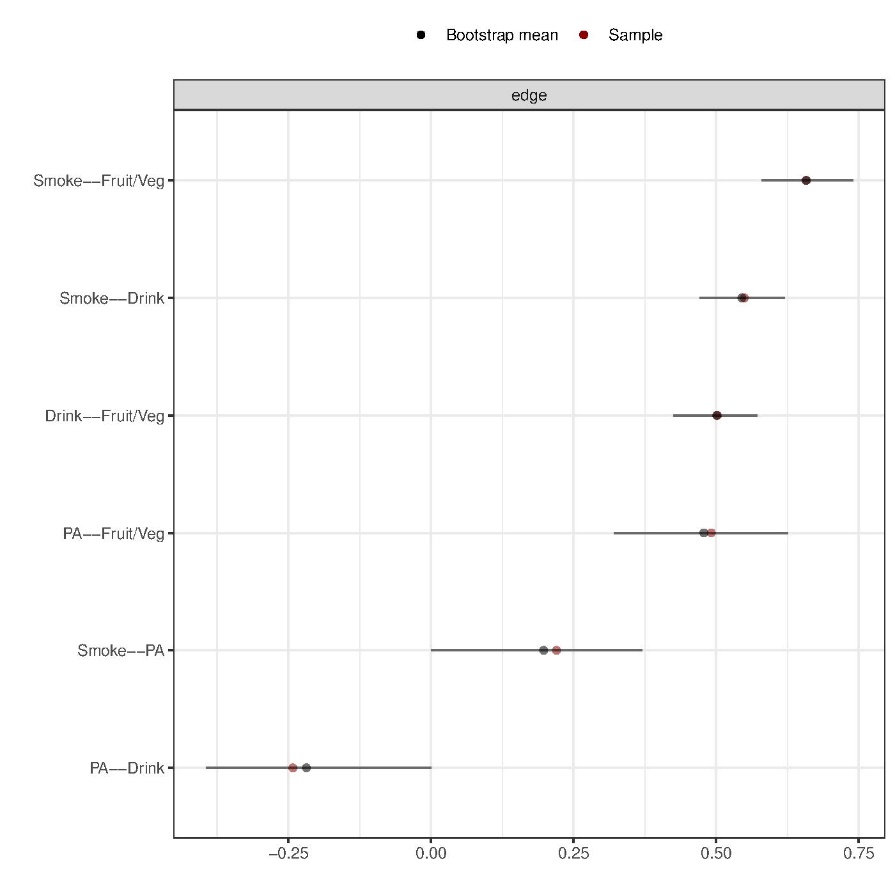


Figure S12: Average correlations between strength and expected influence statistics of the study samples and sub-samples with different amounts of cases dropped for cross-sectional behaviour network for people with (a) and without (b) diabetes

1. People with diabetes


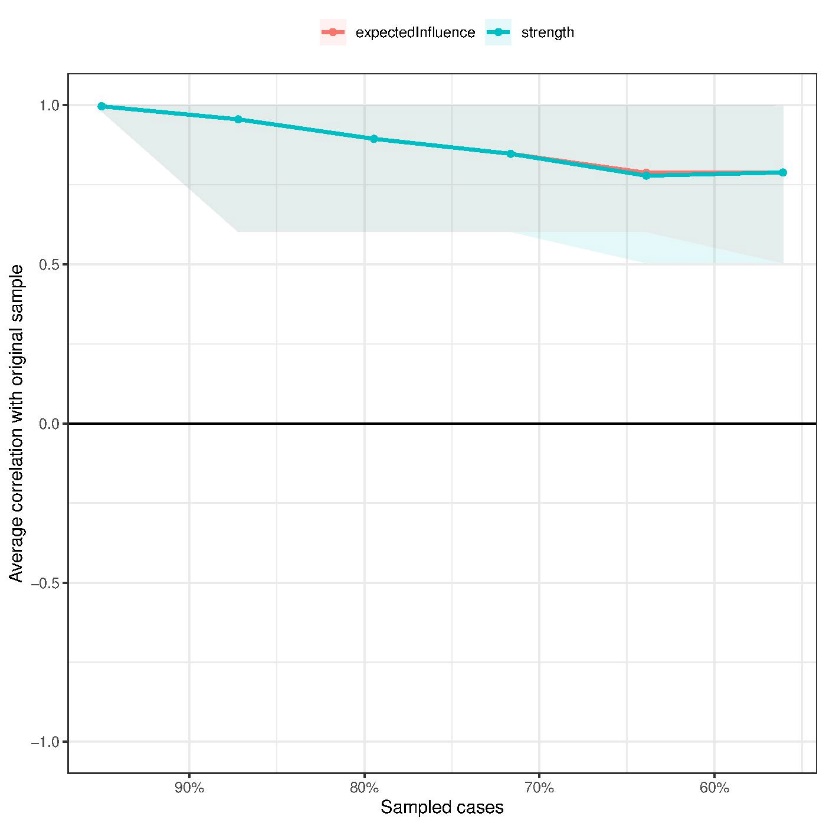


1. People without diabetes


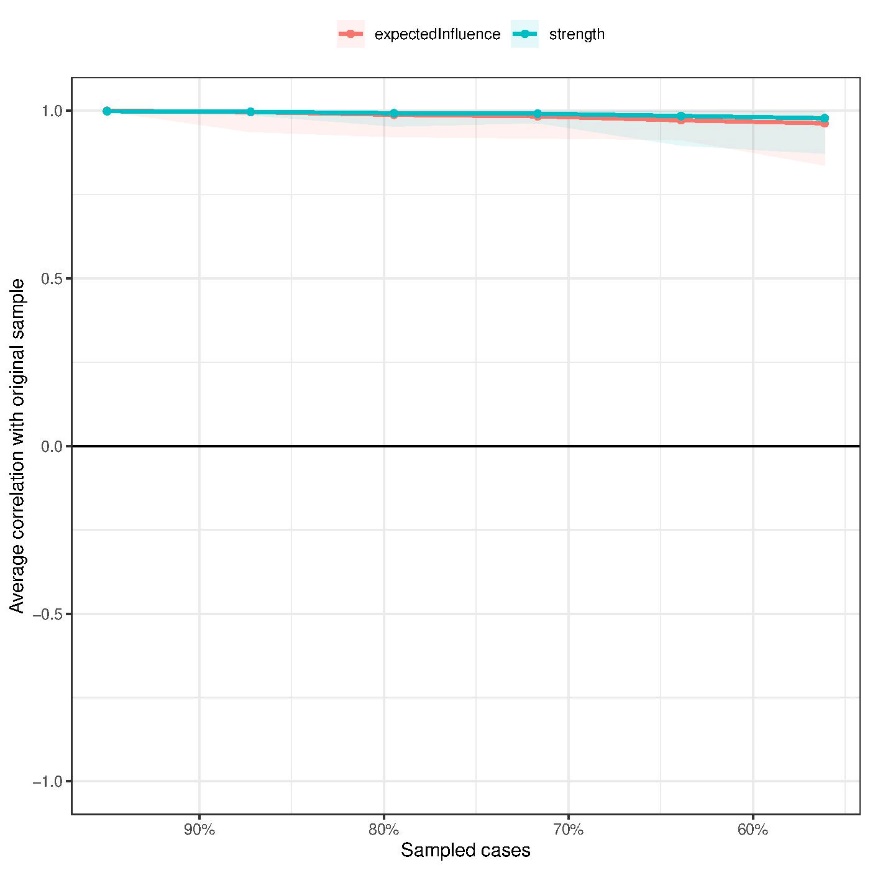


Figure S13: Cross-sectional networks of depressive symptoms for people with (a) and without diabetes (b) with threshold limiting edges to >.24 in edge weight. Circles represent the variables of interest. Lines between nodes represent the associations between them. Blue denotes a positive association, red denotes a positive. The thicker the link the stronger the association. Dep1 = Depression; Dep2 = Pessimism; Dep3 = Suicidality; Dep4 = Guilt; Dep5 = Sleep; Dep6 = Interest; Dep7 =Irritability; Dep8 =Appetite; Dep9 =Fatigue; Dep10= Concentration; Dep11 = Enjoyment; Dep12= Tearfulness

1. People with diabetes


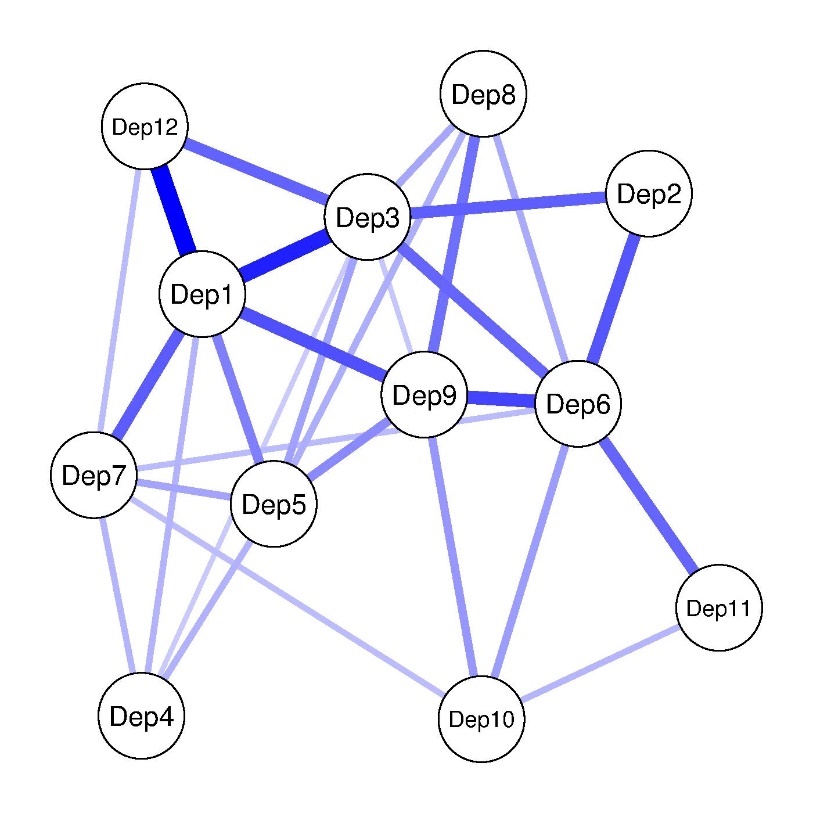


1. People without diabetes


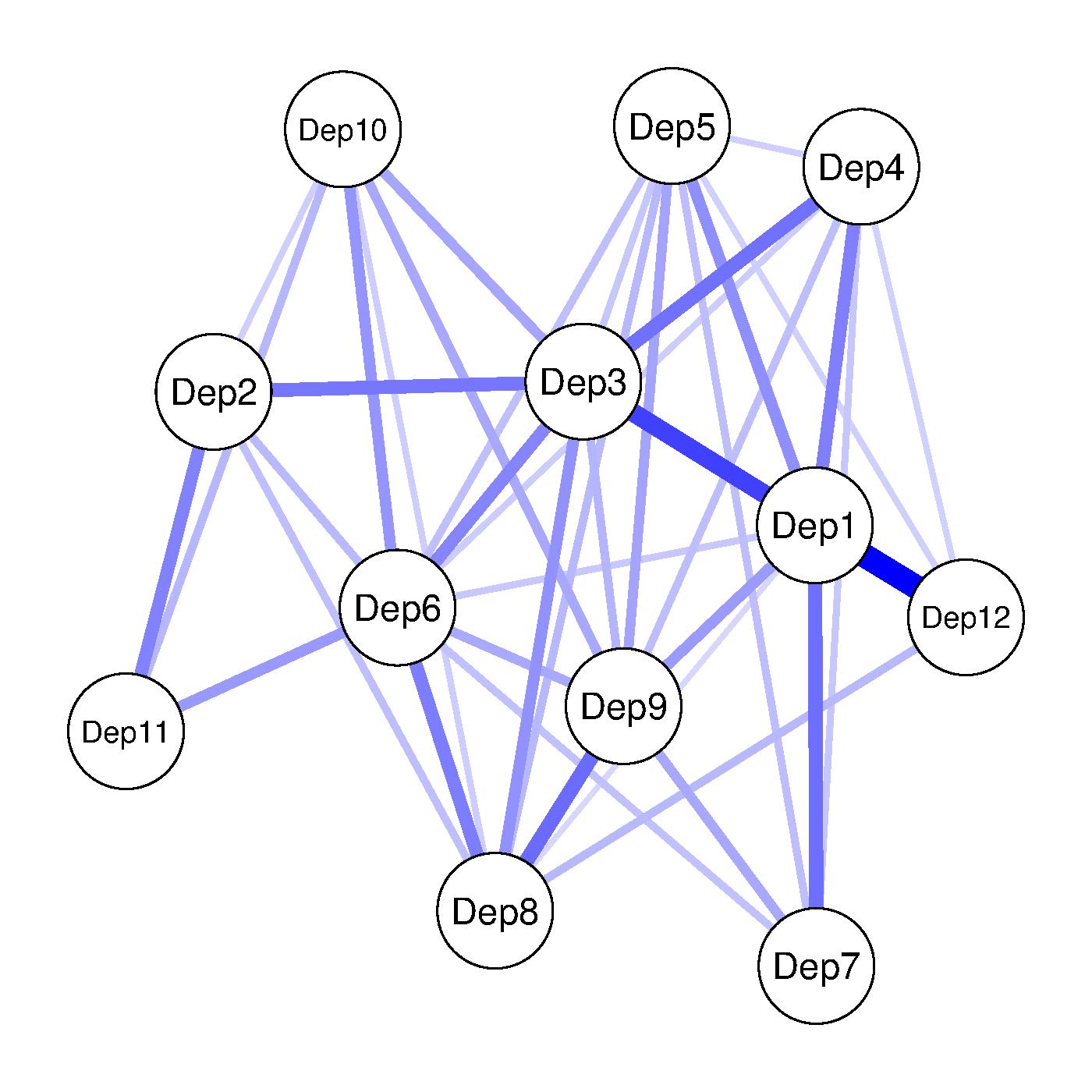


Figure S14: Cross-sectional networks of depressive symptoms for people with (a) and without diabetes (b) with all edges (i.e., threshold not set to >.24 in edge weight). Circles represent the variables of interest. Lines between nodes represent the associations between them. Blue denotes a positive association, red denotes a positive. The thicker the link the stronger the association. Dep1 = Depression; Dep2 = Pessimism; Dep3 = Suicidality; Dep4 = Guilt; Dep5 = Sleep; Dep6 = Interest; Dep7 =Irritability; Dep8 =Appetite; Dep9 =Fatigue; Dep10= Concentration; Dep11 = Enjoyment; Dep12= Tearfulness

1. People with diabetes


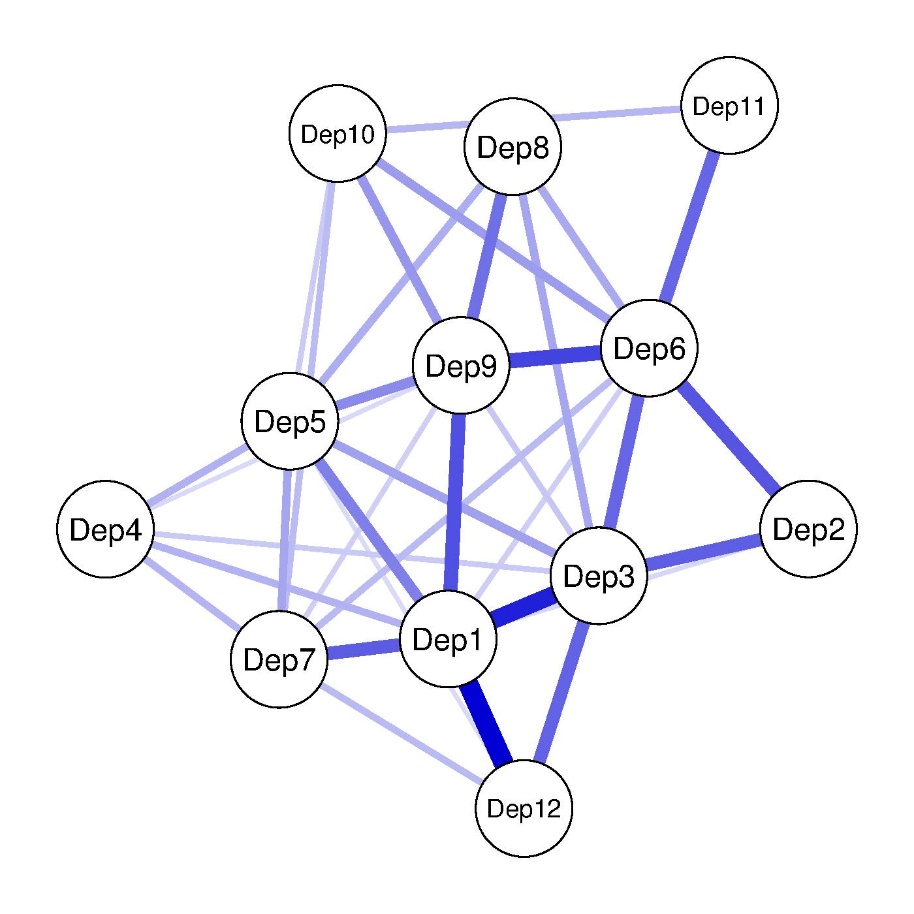


1. People without diabetes


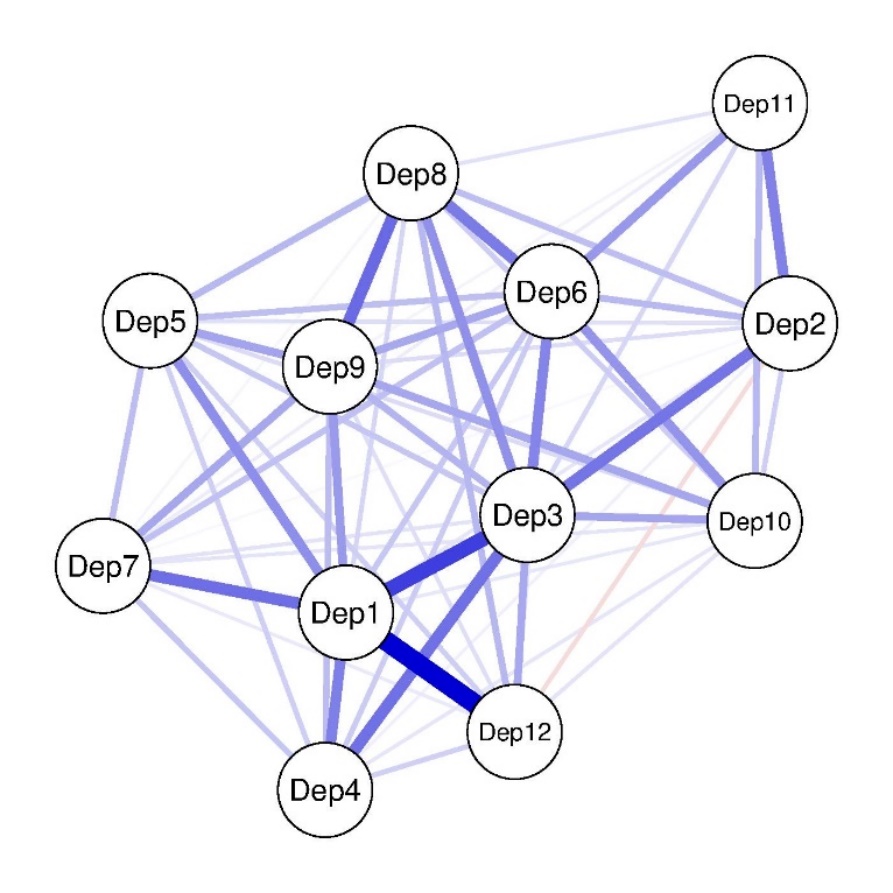


Figure S15: Cross-sectional networks of expected influence and strength for depressive symptom network for people with (a) and without (b) diabetes. Strength presented as z-scores. Dep1 = Depression; Dep2 = Pessimism; Dep3 = Suicidality; Dep4 = Guilt; Dep5 = Sleep; Dep6 = Interest; Dep7 =Irritability; Dep8 =Appetite; Dep9 =Fatigue; Dep10= Concentration; Dep11 = Enjoyment; Dep12= Tearfulness

1. People with diabetes


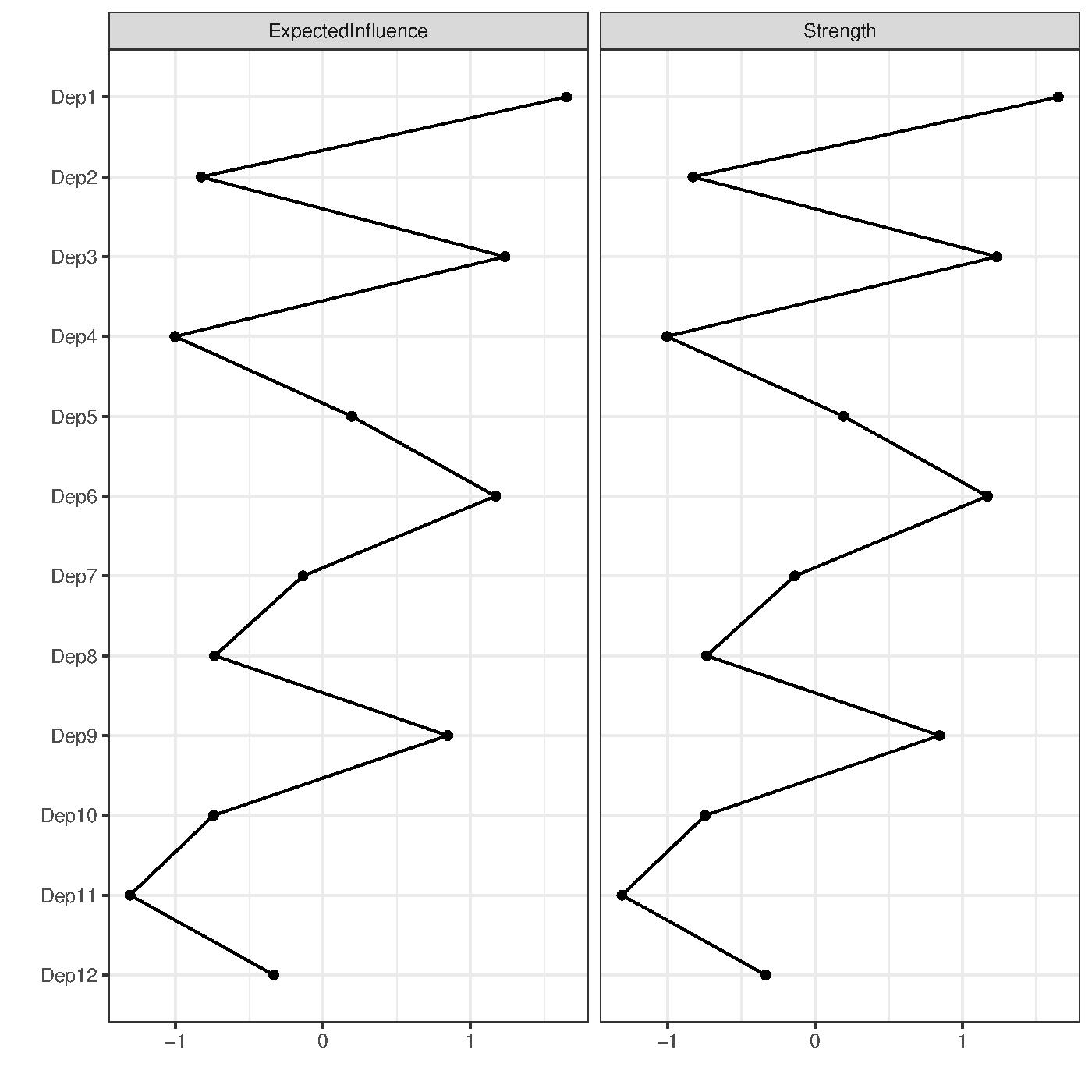


1. People without diabetes


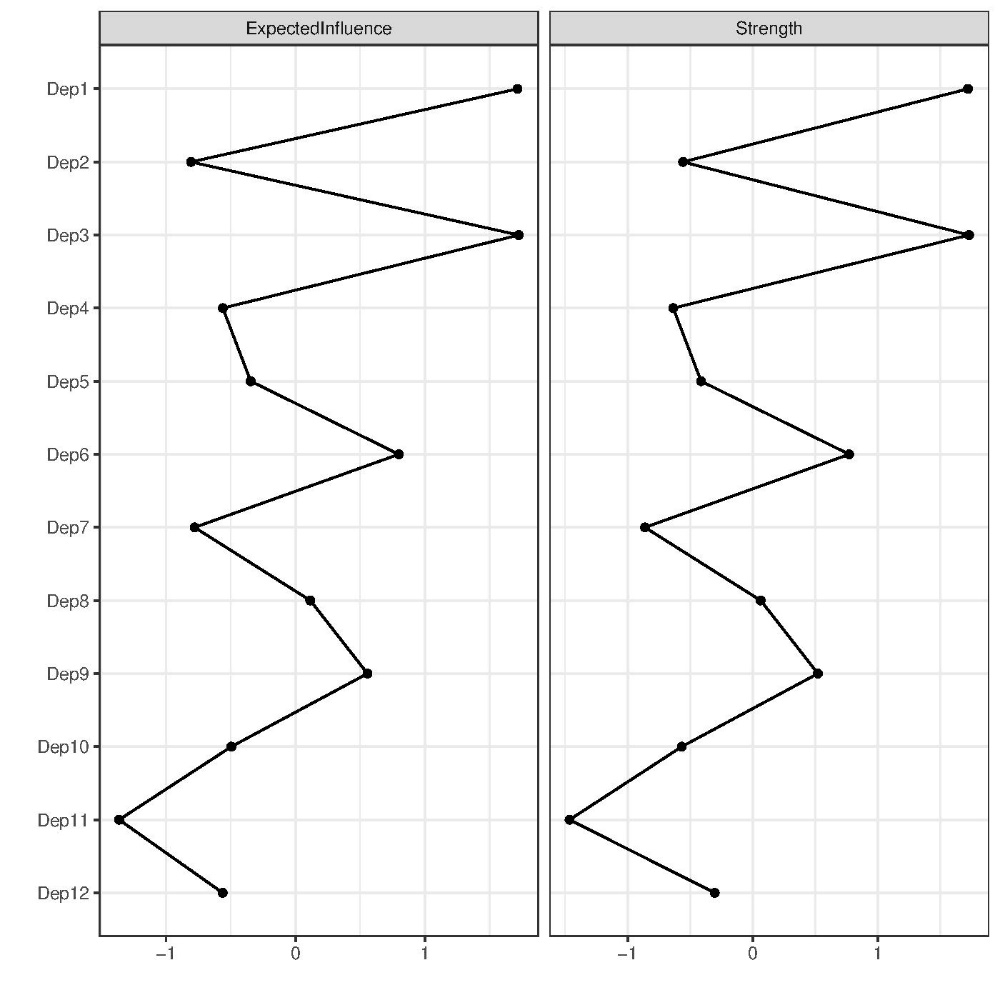


Figure S16: Bootstrapped edge weight confidence intervals for the cross-sectional network of depressive symptom for people with (a) and without (b) diabetes. Edge weights for each edge (connection between nodes) with 95% Confidence Intervals. Dep1 = Depression; Dep2 = Pessimism; Dep3 = Suicidality; Dep4 = Guilt; Dep5 = Sleep; Dep6 = Interest; Dep7 =Irritability; Dep8 =Appetite; Dep9 =Fatigue; Dep10= Concentration; Dep11 = Enjoyment; Dep12= Tearfulness

1. People with diabetes
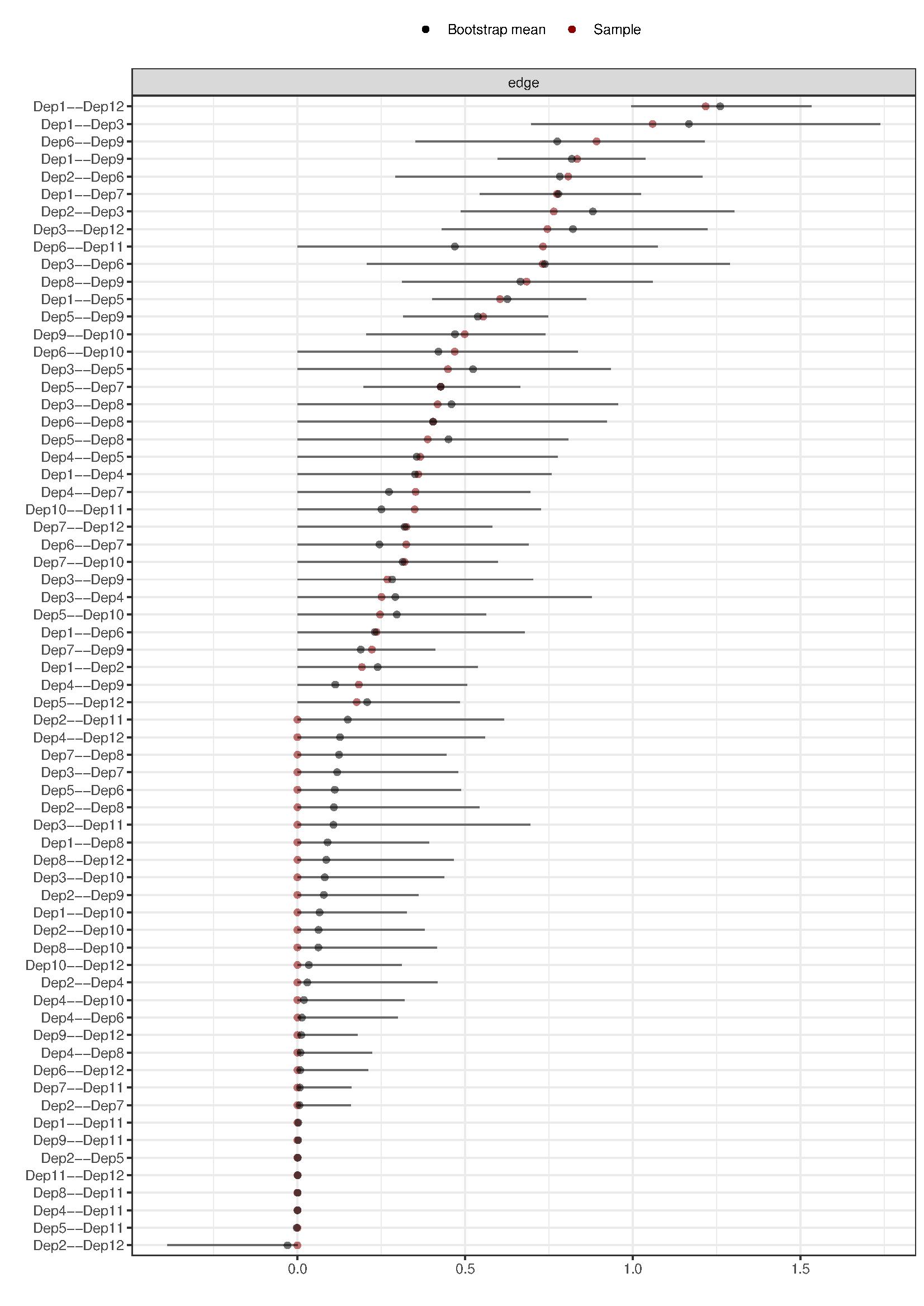

2. People without diabetes


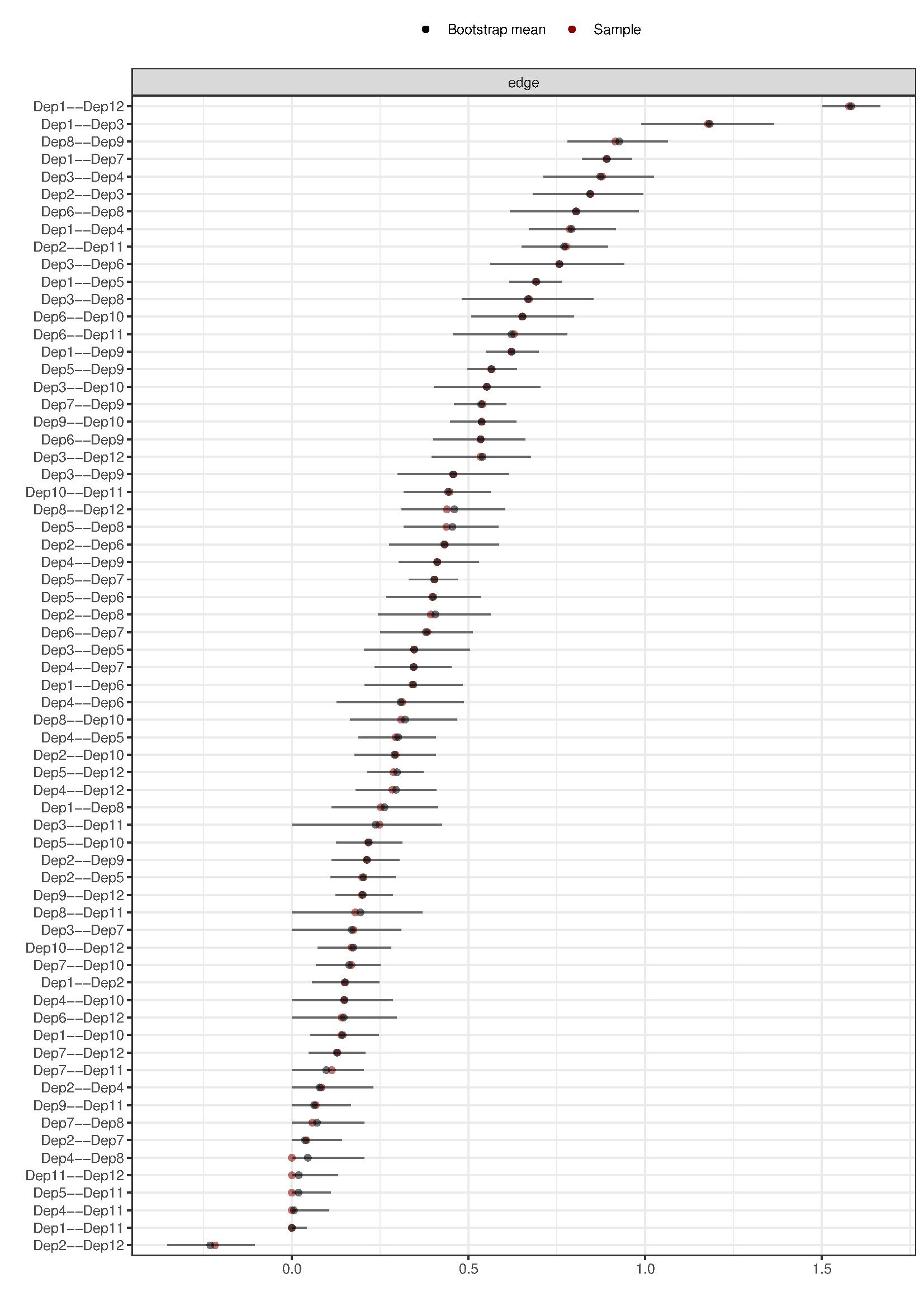


Figure S17: Average correlations between strength and expected influence statistics of the study samples and sub-samples with different amounts of cases dropped for depressive symptom network for people with (a) and without (b) diabetes

1. People with diabetes


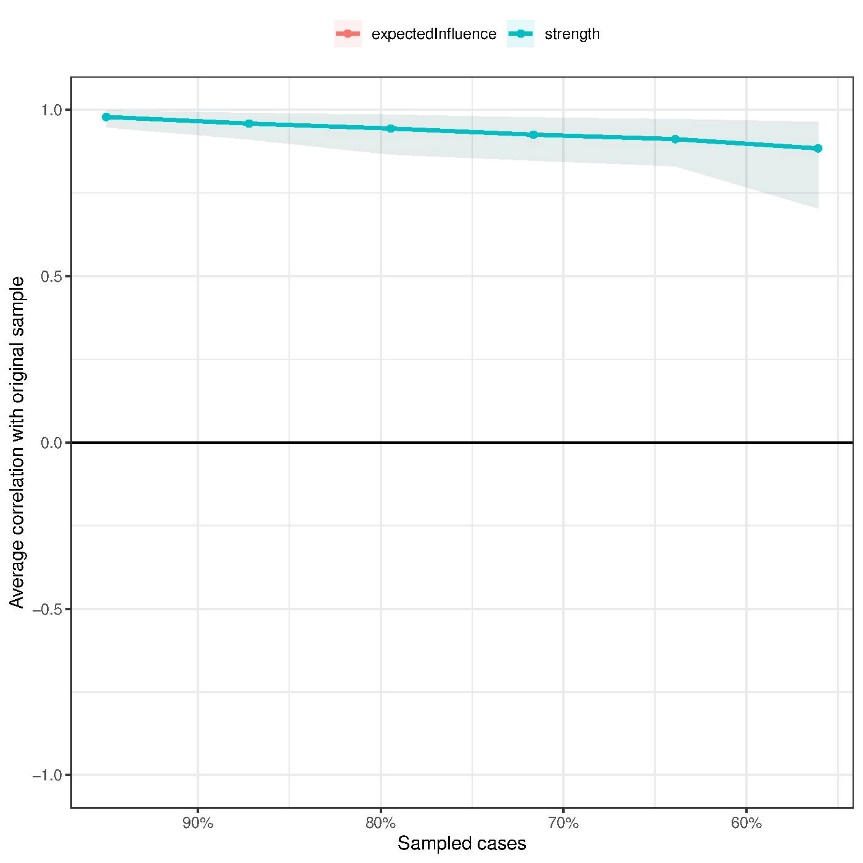


1. People without diabetes


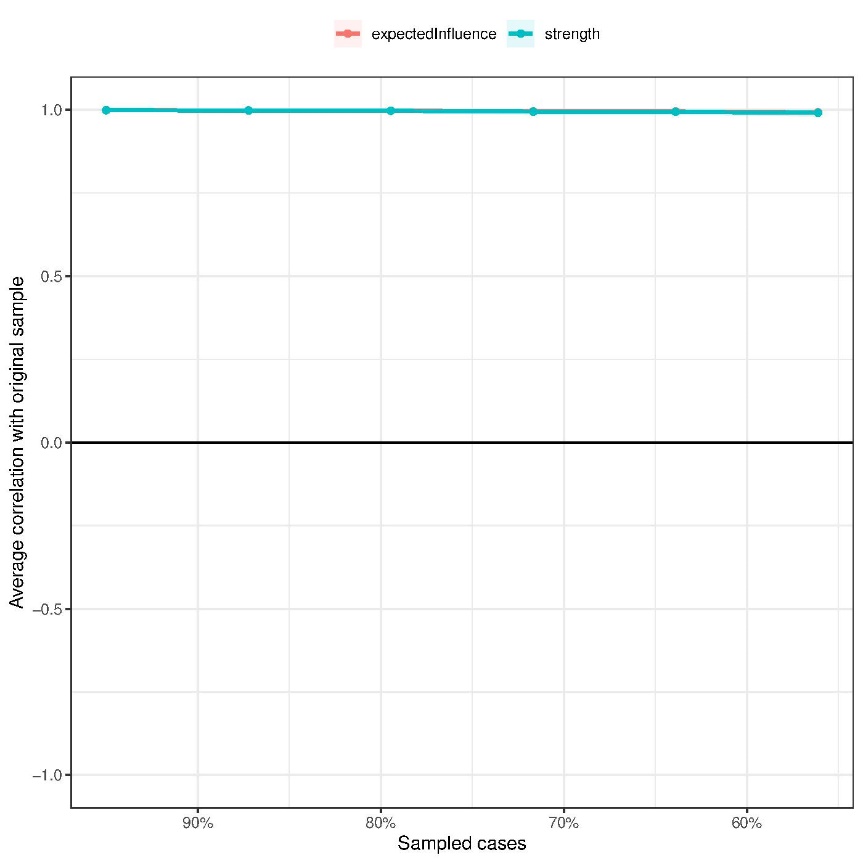


Figure S18: Cross-sectional network of depressive symptoms and health-risk behaviours in people with and without diabetes with all edges (i.e., threshold not set at >.24 in edge weight)

1. People with diabetes


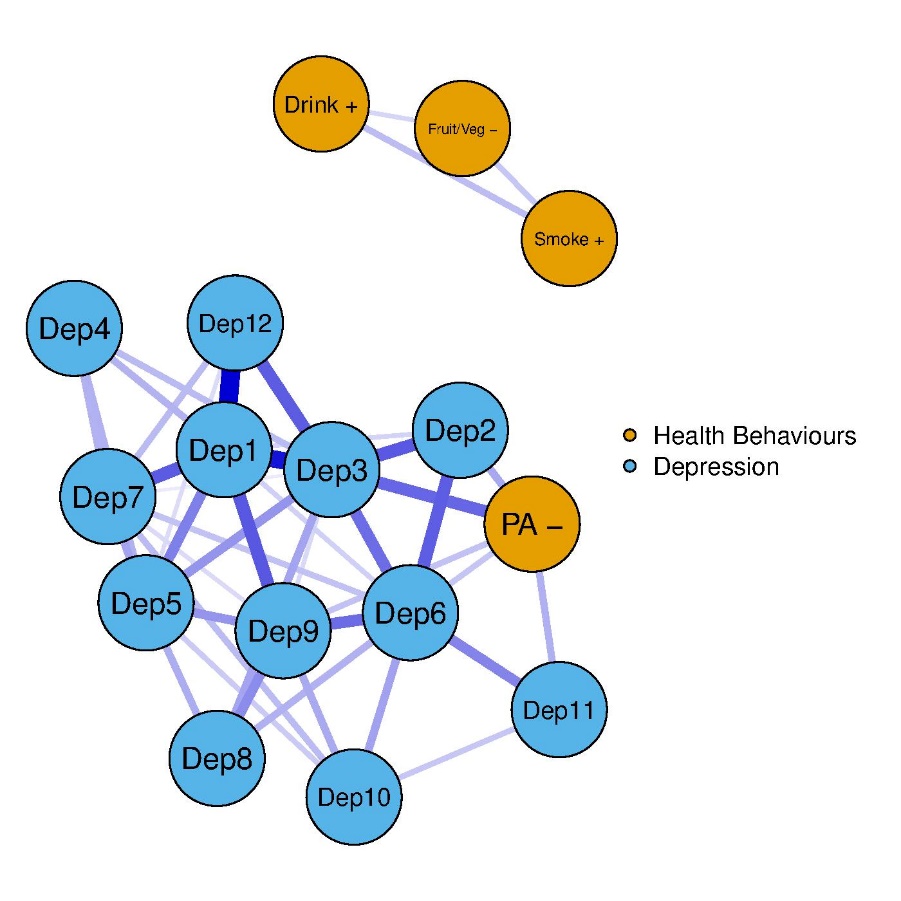


1. People without diabetes


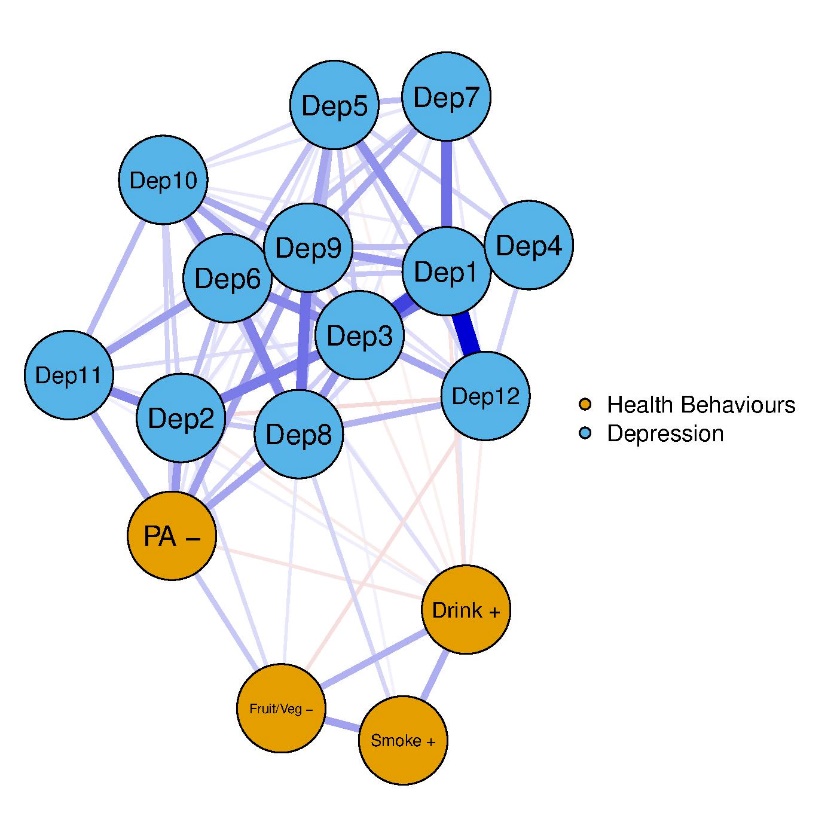


Figure S19: Bootstrapped edge weight confidence intervals for the cross-sectional network of depressive symptom and health behaviours for people with (a) and without (b) diabetes. Edge weights for each edge (connection between nodes) with 95% Confidence Intervals. Dep1 = Depression; Dep2 = Pessimism; Dep3 = Suicidality; Dep4 = Guilt; Dep5 = Sleep; Dep6 = Interest; Dep7 =Irritability; Dep8 =Appetite; Dep9 =Fatigue; Dep10= Concentration; Dep11 = Enjoyment; Dep12= Tearfulness

1. People with diabetes


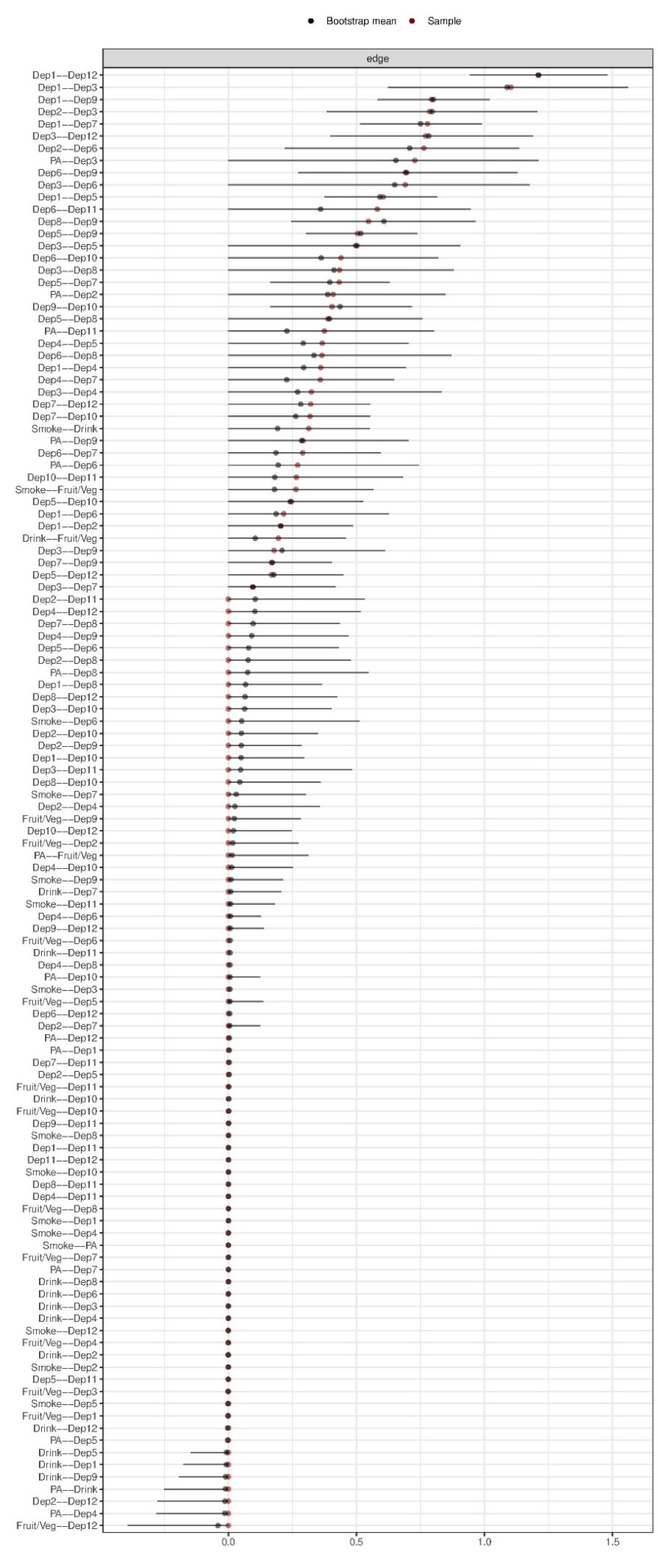


1. People without diabetes


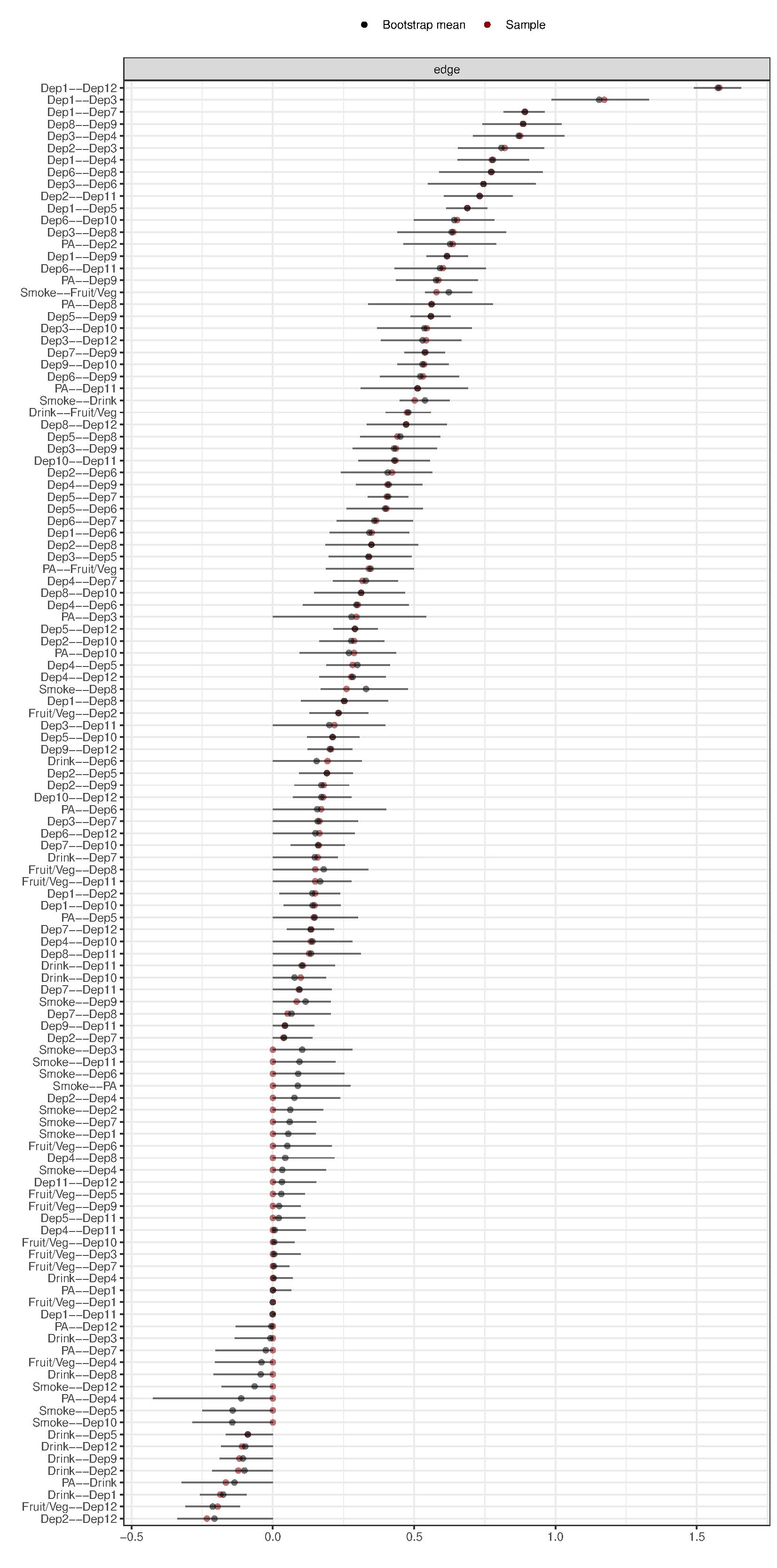


Figure S20: Average correlations between strength and expected influence statistics of the study samples and sub-samples with different amounts of cases dropped for depressive symptom and health-risk behaviour network for people with (a) and without (b) diabetes

1. People with diabetes


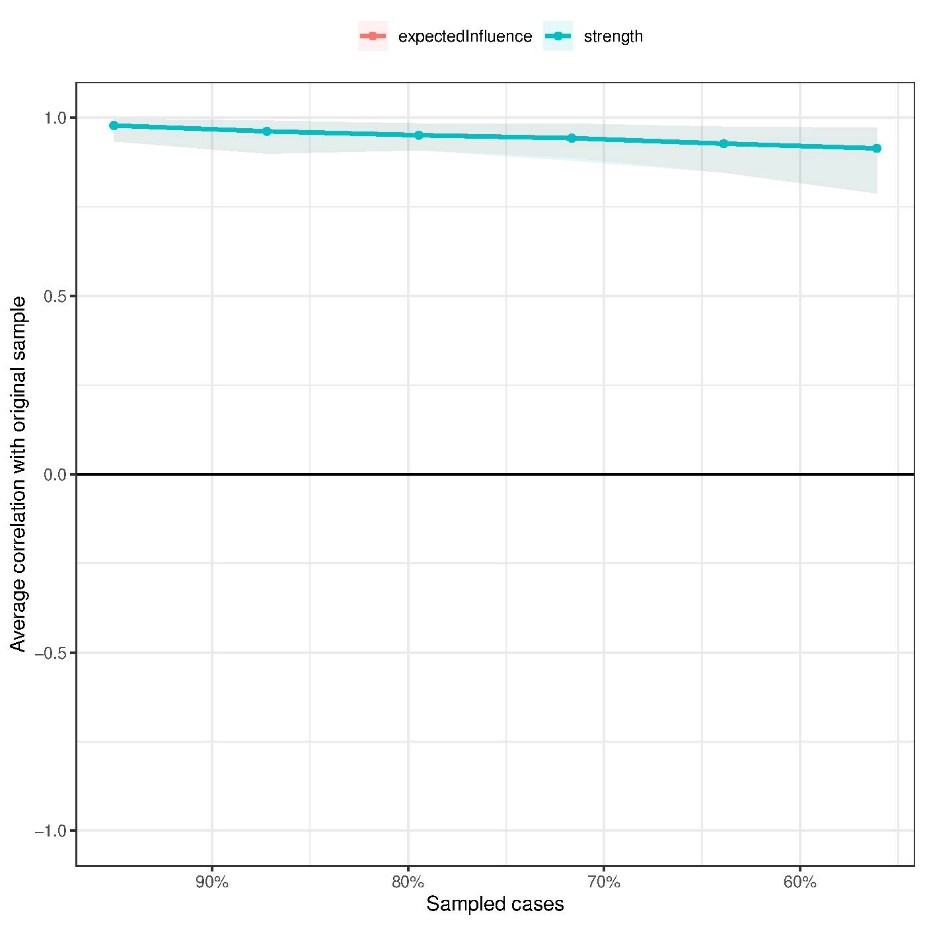


1. People without diabetes


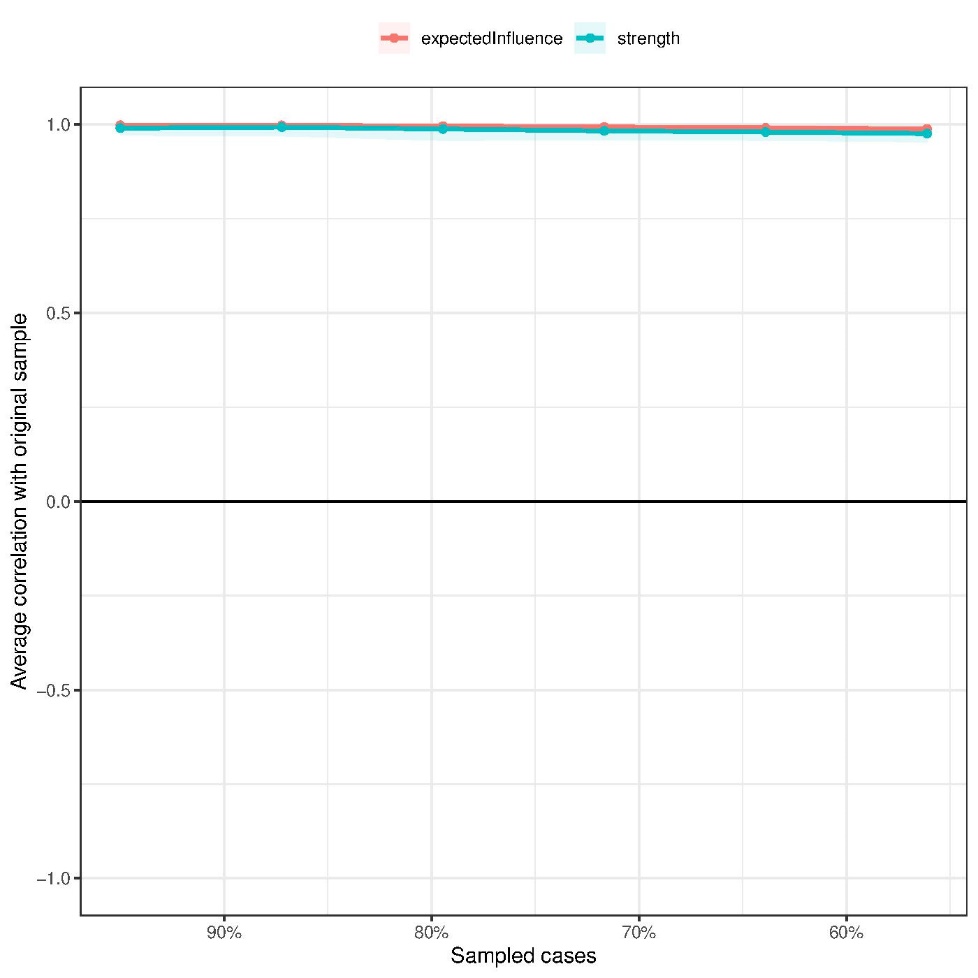


Figure S21: Bridge strength and bridge expected influence from cross-sectional combined networks (original main study sample). Not stable for people with diabetes. Dep1 = Depression; Dep2 = Pessimism; Dep3 = Suicidality; Dep4 = Guilt; Dep5 = Sleep; Dep6 = Interest; Dep7 =Irritability; Dep8 =Appetite; Dep9 =Fatigue; Dep10= Concentration; Dep11 = Enjoyment; Dep12= Tearfulness; Smoke + = present smoker; PA - = Physically inactivity; Fruit/veg -; doesn’t eat fruit/veg daily; Drink + = drank heavily at least once in last 3 months.

1. People with diabetes


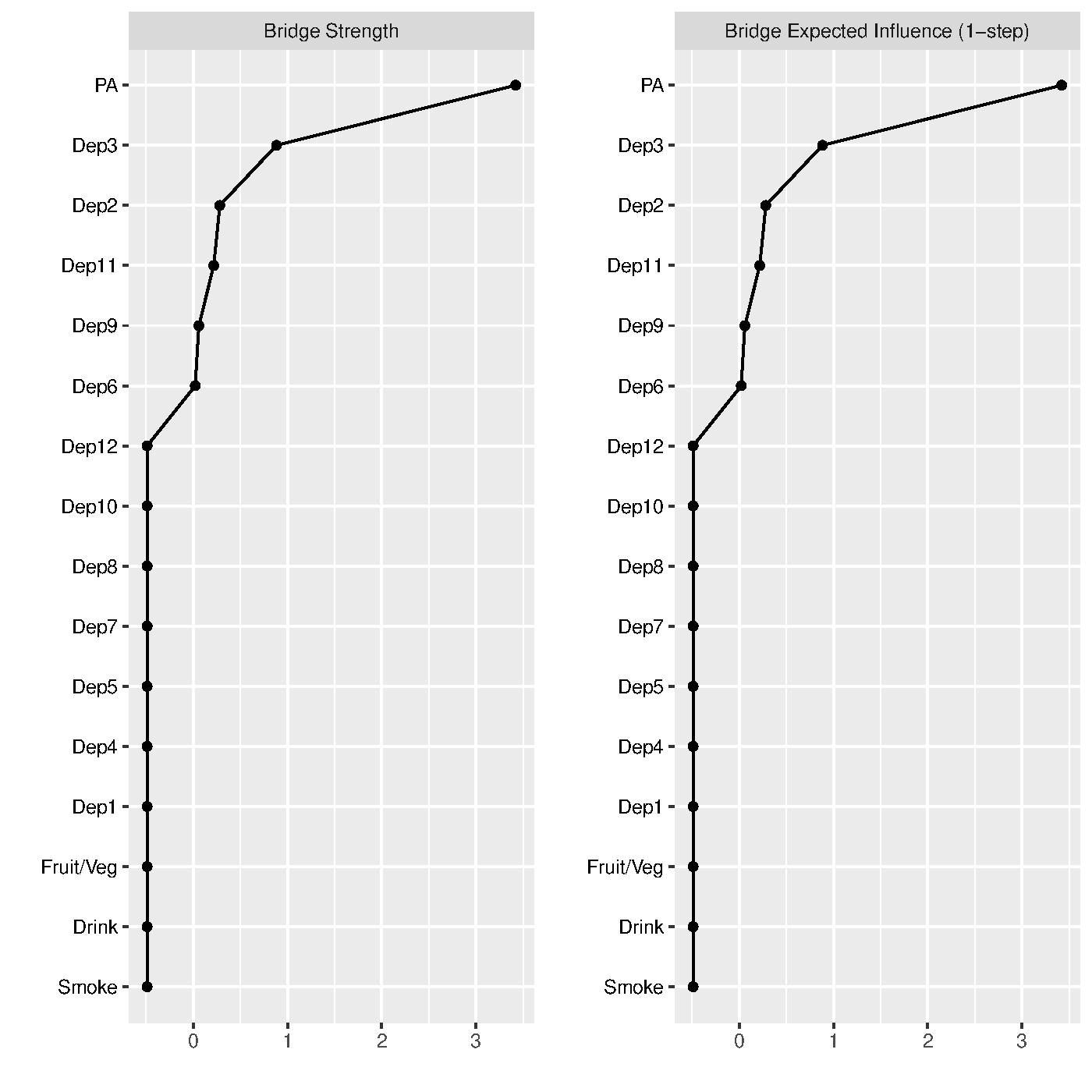


1. People without diabetes


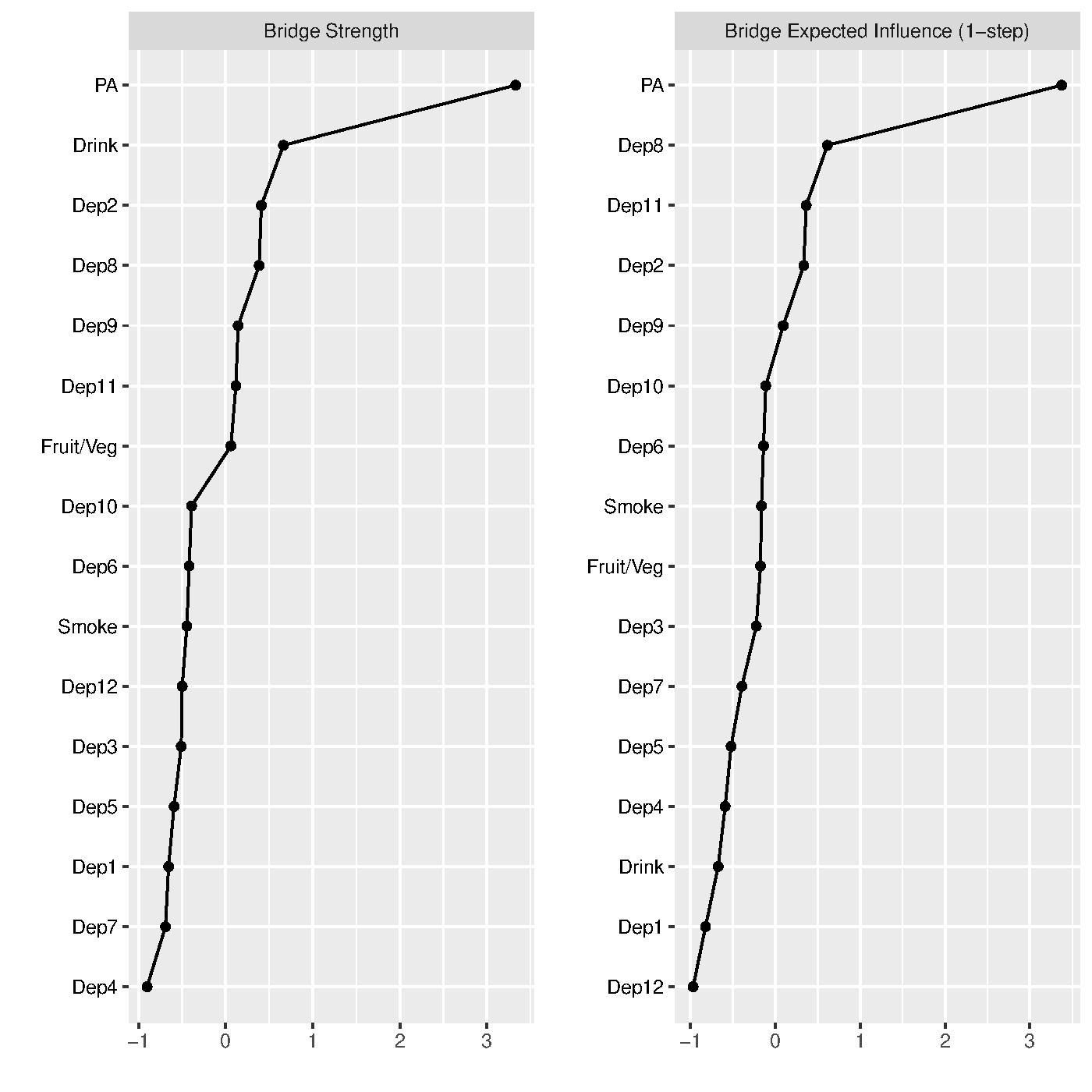


Table S6: Sample characteristics for larger additional analysis, with those included in the main analysis (e.g., present at both wave 4 and wave 5) and those not included in the main study analysis (e.g., those not present in wave 5) compared.

|  | **Sub-samples of larger additional analysis** | |  | **Larger additional analysis** |
| --- | --- | --- | --- | --- |
|  | Main study sample  N (%)/M (SD) | Those in larger sample but not in main study sample  N (%)/M (SD) | Difference Test * | N (%)/M (SD) |
| N | 22,510 (51.6%) | 21,142 (48.4%) |  | 43,652 |
| Age | 63.9 (9.3) | 65.2 (10.5) | <0.001 | 64.5 (10) |
| Male or female |  |  |  |  |
| Male | 11,112 (49.4%) | 9,046 (42.8%) | <0.001 | 20,158 (46.2%) |
| Female | 11,398 (50.6%) | 12,096 (57.2%) |  | 23,494 (53.8%) |
| Marital status |  |  |  |  |
| Married and living together with spouse | 9,423 (68.2%) | 11,079 (67.6%) | <0.001 | 20,502 (67.8%) |
| Registered partnership | 236 (1.7%) | 258 (1.6%) |  | 494 (1.6%) |
| Married, living separated from spouse | 198 (1.4%) | 202 (1.2%) |  | 400 (1.3%) |
| Never married | 871 (6.3%) | 903 (5.5%) |  | 1,774 (5.9%) |
| Divorced | 1,533 (11.1%) | 1,401 (8.5%) |  | 2,934 (9.7%) |
| Widowed | 1,565 (11.3%) | 2,549 (15.6%) |  | 4,114 (13.6%) |
| Years education | 11.2 (4.4) | 10.1 (4.3) | <0.001 | 10.6 (4.4) |
| Number of limitations with activities of daily living (adl) |  |  |  |  |
| 0 | 20,995 (93.3%) | 18,596 (88.0%) | <0.001 | 39,591 (90.7%) |
| 1 | 1,035 (4.6%) | 1,350 (6.4%) |  | 2,385 (5.5%) |
| 2 | 254 (1.1%) | 566 (2.7%) |  | 820 (1.9%) |
| 3 | 132 (0.6%) | 252 (1.2%) |  | 384 (0.9%) |
| 4 | 33 (0.1%) | 162 (0.8%) |  | 195 (0.4%) |
| 5 | 29 (0.1%) | 119 (0.6%) |  | 148 (0.3%) |
| 6 | 28 (0.1%) | 90 (0.4%) |  | 118 (0.3%) |
| BMI categories |  |  |  |  |
| Below 18.5 - underweight | 211 (1.0%) | 235 (1.2%) | <0.001 | 446 (1.1%) |
| 18.5-24.9 - normal | 8,517 (39.1%) | 6,820 (34.0%) |  | 15,337 (36.7%) |
| 25-29.9 - overweight | 9,106 (41.8%) | 8,286 (41.4%) |  | 17,392 (41.6%) |
| 30 and above - obese | 3,964 (18.2%) | 4,693 (23.4%) |  | 8,657 (20.7%) |
| Self-perceived health - us version |  |  |  |  |
| Excellent | 2,261 (10.0%) | 1,136 (5.4%) | <0.001 | 3,397 (7.8%) |
| Very good | 5,004 (22.2%) | 3,047 (14.4%) |  | 8,051 (18.4%) |
| Good | 8,868 (39.4%) | 7,322 (34.6%) |  | 16,190 (37.1%) |
| Fair | 5,198 (23.1%) | 6,681 (31.6%) |  | 11,879 (27.2%) |
| Poor | 1,174 (5.2%) | 2,950 (14.0%) |  | 4,124 (9.4%) |
| EURO-D caseness (wave 4) |  |  |  |  |
| No | 17,688 (78.6%) | 14,444 (68.3%) | <0.001 | 32,132 (73.6%) |
| Yes | 4,822 (21.4%) | 6,698 (31.7%) |  | 11,520 (26.4%) |
| Smoking status (wave 4) |  |  |  |  |
| Not present smoker | 17,988 (79.9%) | 17,177 (81.2%) | <0.001 | 35,165 (80.6%) |
| Present smoker | 4,522 (20.1%) | 3,965 (18.8%) |  | 8,487 (19.4%) |
| Physical inactivity (wave 4) |  |  |  |  |
| Other | 21,347 (94.8%) | 18,369 (86.9%) | <0.001 | 39,716 (91.0%) |
| Never vigorous nor moderate physical activity | 1,163 (5.2%) | 2,773 (13.1%) |  | 3,936 (9.0%) |
| How often serving of fruits or vegetables |  |  |  |  |
| eats daily | 17,716 (78.7%) | 15,704 (74.3%) | <0.001 | 33,420 (76.6%) |
| eat less than every day | 4,794 (21.3%) | 5,438 (25.7%) |  | 10,232 (23.4%) |
| How often six or more drinks last 3 months |  |  |  |  |
| Never drinks more than six drinks | 16,691 (74.1%) | 17,130 (81.0%) | <0.001 | 33,821 (77.5%) |
| Drank >6 drinks at least once in last 3 months | 5,819 (25.9%) | 4,012 (19.0%) |  | 9,831 (22.5%) |
| Notes. BMI =Body mass index; EURO-D = Euro depression scale; Equality between groups was tested using Pearson χ2 for categorical variables and linear regression for continuous variables. | | | | |
